# All atom insights into the impact of crowded environments on protein stability by NMR spectroscopy

**DOI:** 10.1038/s41467-020-19616-w

**Published:** 2020-11-13

**Authors:** Birgit Köhn, Michael Kovermann

**Affiliations:** 1 Department of Chemistry, University of Konstanz, Universitätsstrasse. 10, 78457 Konstanz, Germany; 2 Konstanz Research School Chemical Biology KoRS-CB, University of Konstanz, Universitätsstrasse. 10, 78457 Konstanz, Germany

**Keywords:** Biophysical chemistry, NMR spectroscopy

## Abstract

The high density of macromolecules affecting proteins due to volume exclusion has been discussed in theory but numerous in vivo experiments cannot be sufficiently understood taking only pure entropic stabilization into account. Here, we show that the thermodynamic stability of a beta barrel protein increases equally at all atomic levels comparing crowded environments with dilute conditions by applying multidimensional high-resolution NMR spectroscopy in a systematic manner. Different crowding agents evoke a pure stabilization cooperatively and do not disturb the surface or integrity of the protein fold. The here developed methodology provides a solid base that can be easily expanded to incorporate e.g. binding partners to recognize functional consequences of crowded conditions. Our results are relevant to research projects targeting soluble proteins in vivo as it can be anticipated that their thermodynamic stability increase comparably and has consequently to be taken into account to coherently understand intracellular processes.

## Abstract (web summary)

The precise effects of crowding on protein folding have been difficult to establish. Here the authors apply multidimensional high-resolution NMR spectroscopy to provide insight on the local impact of macromolecular crowding on the thermodynamic stability of proteins.

## Introduction

All essential processes of life occur within the cellular environment and are therein crowded by high concentrations of macromolecules, such as proteins and nucleic acids. For the cellular lumen of *E. coli*, a volume fraction of 20–40% of macromolecules has been reported thus contributing to an overall concentration of up to 400 g/L of macromolecules which are present in a living cell^1,2^. Consequently, this dense environment has to be considered in the study of protein stability, dynamics, and function since the wealth of various macromolecules affects local concentrations of proteins and reactants^3,4^, potentially hinders molecular diffusion^5,6^ and has multiple, highly complex effects on molecular interactions in the cytosol^7–9^. In this respect, it has indeed been shown that experimental results that are exclusively based on in vitro investigations of proteins, which have been isolated from their cellular environment are possibly misleading in conclusions drawn about their inherent stability, and conformational dynamics that are relevant for performing intracellular interactions. Thus it has been reported that macromolecular crowding (MC) has pronounced effects in numerous cases as it was found e.g. for FlgM, an intrinsically disordered protein, that it gains a distinct structure upon crowding^10^ or for the enzyme Pin1, that it is able to form unexpected complexes in a crowded environment^11^. Further, protein kinetics^12^ and dynamics^13,14^, enzymatic reaction rate constants^15^, ribonucleic acid folding stability^16^, ribozyme activity^17^, and the cytoskeletal properties itself^18^ were shown to be altered under MC. Moreover, the association of proteins is influenced by MC differing strongly from in vitro results in respect to aggregation^19^ and amyloid fibril formation^20,21^. Regarding protein folding and stability it was found that MC leads to a narrower folding funnel concerning the native state^22^, to a change in solvent accessibility^23^ or to a compaction of intrinsically disordered proteins^24^ or an unfolded protein ensemble^25^.

**Fig. 1:**
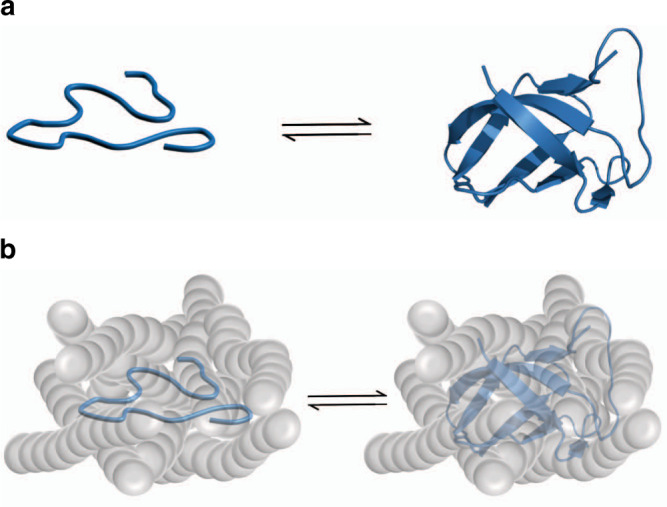
Probing folding-to-unfolding equilibrium of *Bs*CspB at different conditions. The application of dilute conditions is shown in **a** whereas the presence of macromolecular crowding agents is highlighted in **b** by using ellipsoids colored in gray. The PDB ID 1NMG has been used to illustrate the protein backbone of *Bs*CspB representing the native state (right). One structure indicating the unfolded protein ensemble of *Bs*CspB has been modeled (left).

Despite those advances, a consistent universal model of the general influence of MC on the thermodynamic stability and conformational space proteins naturally occupy is missing. To fill this gap the field of MC advances top-down approaches. Here, protein signals are recorded in whole cells or in cellular lysates before degradation of reporter proteins takes place due to the cellular proteostasis machinery that is inherently present in vivo. Those measurements yield indeed fundamental results^26,27^ but are limited in spatial resolution and are not easily reproducible since the composition of cells, and their cellular extract is highly diverse and strongly depends on individual culturing conditions. Therefore, we have chosen here a bottom-up approach to investigate effects of MC on protein stability using precisely defined conditions. This setup presents no restrictions concerning experimental time due to the prevention of potential degradation of the sample under investigation. One can achieve the highest spectral resolution possible in order to monitor effects of MC at a residue and even an atomistic level on the protein under study. Here, we present an analysis of the thermodynamic stability of the cold shock protein B from *Bacillus subtilis*, *Bs*CspB, at atomic resolution by probing almost all ^15^N and ^13^C bound protons. *Bs*CspB as a prominent model protein representing two-state folding (Fig. 1) has been chosen here as it has been structurally well characterized by using X-ray crystallography and high-resolution NMR spectroscopy before^28,29^, and its thermodynamic stability has been thoroughly investigated under dilute conditions applying multiple experimental techniques^30–33^. Here, the experimentally obtained data have been monitored by conducting chemically induced unfolding of *Bs*CspB in absence and presence of MC applying high-resolution heteronuclear multidimensional NMR spectroscopy. We have acquired both two-dimensional heteronuclear ^1^H-^15^N as well as ^1^H-^13^C HSQC spectra of *Bs*CspB, respectively, varying in the concentration of urea ranging between *c*^urea^ = 0 M and *c*^urea^ = 6.3 M. The globular sugar polymer dextran having 20 kDa (Dex20) and the smaller, less polar polyethylene glycol of 1 kDa molecular weight (PEG1) have been used as crowding agents. This strategic choice is based on a recent finding obtained by applying a combination of fluorescence, circular dichroism and NMR spectroscopy showing that PEG and Dex20 molecules have a pronounced effect on the overall thermodynamic stability of *Bs*CspB^23^. It could be shown that the presence of equally concentrated solutions containing PEG1, PEG8, PEG35, or Dex20 molecules impacts the overall thermodynamic stability of *Bs*CspB quantitatively in the same manner regardless of differences in molecular weight and polarity. Selecting PEG1 and Dex20 as done here enables to perform highly-resolved NMR spectroscopic experiments on a protein, which is significantly larger than the used crowding molecules regarding PEG1 on the one hand or smaller regarding Dex20 on the other hand.

Beyond that the present study aims to expand the current knowledge regarding the impact the crowded environment possesses on protein thermodynamic stability by mapping NMR derived data at single residue resolution to resonances originating from the main chain, and even from side chain methylene and methyl groups. This procedure enables to obtain a profound understanding of crowding effects on a protein at an atomic level.

## Results

### Unfolding of *Bs*CspB under dilute conditions and in crowded environments monitoring ^1^H signals collectively

**Fig. 2:**
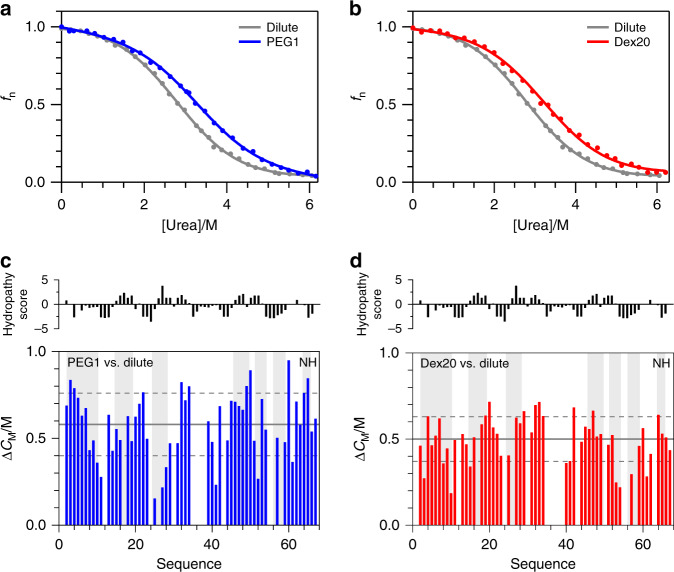
Thermodynamic stability of *Bs*CspB monitored via following folding-to-unfolding transitions induced by urea. **a**, **b** Analysis of aliphatic protons representing the native state, *f*_n_, observed by one-dimensional ^1^H NMR spectroscopy in absence of MC (colored in gray), in presence of *c* = 120 g/L PEG1 (colored in blue, **a**), and *c* = 120 g/L Dex20 (colored in red, **b**). **c**, **d** Difference in the transition midpoint, Δ*C*_M_, of amide protons observed in two-dimensional ^1^H-^15^N HSQC NMR spectra comparing dilute conditions with the presence of *c* = 120 g/L PEG1 (colored in blue, **c**) or *c* = 120 g/L Dex20 (colored in red, **d**). Beta sheet regions according to PDB ID 1NMG are indicated by using a background colored in gray. The average of Δ*C*_M_ is shown by the horizontal line (continuous mode) whereas the mean plus or minus one standard deviation of Δ*C*_M_ is represented by two additional horizontal lines (dotted mode). The hydropathy score^76^ concerning *Bs*CspB has been shown on top by using the online tool ProtScale available at the Bioinformatics Resource Portal ExPASy^77^.

First, unfolding of *Bs*CspB induced by an increasing concentration of urea has been monitored by applying one-dimensional ^1^H NMR spectroscopy (Supplementary Figs. 1a and 2). Under dilute conditions the midpoint of this transition, *C*_M_, has been determined to *C*_M_ = 2.7 M urea (Fig. 2a, b) using aliphatic protons comprising *Bs*CspB. This is in accordance to the result obtained by fluorescence spectroscopy, where a transition midpoint of *C*_M_ = 2.5 M urea was found^23^. As expected, addition of *c* = 120 g/L PEG1 to the buffer leads to a significant increase in thermodynamic stability of *Bs*CspB represented by a shift in *C*_M_ of about 0.6 M urea resulting in *C*_M_ = 3.3 M urea (Fig. 2a and Supplementary Table 1). This increase in thermodynamic stability of *Bs*CspB is repeatedly observed for the addition of *c* = 120 g/L Dex20 (Fig. 2b and Supplementary Table 1). Notably, the gain in thermodynamic stability which has been observed here by using one-dimensional proton NMR spectroscopy is qualitatively in line with probing intrinsic fluorescence and circular dichroism following the unfolding of *Bs*CspB^23^. Focusing on the change in free energy by unfolding, ${\Delta}G_{\text{N} \leftrightarrow \text{U}}^0$, we determined ${\Delta}G_{\text{N} \leftrightarrow \text{U}}^0$ = 8.4 kJ/mol under dilute conditions, compared to 9.7 kJ/mol in the presence of 120 g/L Dex20 and 9.8 kJ/mol in the presence of 120 g/L PEG1 (Supplementary Table 1). This difference in the change of free energy of unfolding, ${\Delta}{\Delta}G_{\text{N} \leftrightarrow \text{U}}^0$, of 1.3 kJ/mol for Dex20 and 1.4 kJ/mol for PEG1, resembles the range of change in ${\Delta}G_{\text{N} \leftrightarrow \text{U}}^0$ that would be anticipated from considerations of the excluded volume theory, a crowding model that has been intensively discussed in literature since Minton and Wilf coined the term of macromolecular crowding^34^. Following a pure excluded volume model as discussed by Christiansen et al.^35^, there is an expected gain in stability, ΔΔ*G*_N↔U_, of about 3.5 kJ/mol by addition of 120 g/L Dex20, which is in the range of our results considering 3.6 kJ/mol for 100 g/L Dex20 measured by fluorescence spectroscopy^23^, and 1.3 kJ/mol for 120 g/L Dex20 measured by one-dimensional proton NMR spectroscopy. Comparing different spectroscopic techniques though, the absolute values of ${\Delta}{\Delta}G_{\text{N} \leftrightarrow \text{U}}^0$ slightly differ, which is inherent to the methodology as the applied techniques differ in the structural features they monitor. Here, we focus on the evaluation of atomically resolved NMR spectroscopic data monitoring the transition region of protein unfolding and do not include data extending to a concentration of 0 M urea, where the absolute value of ${\Delta}G_{\text{N} \leftrightarrow \text{U}}^0$ can be precisely estimated. Consequently, we choose the change of the midpoint of protein unfolding, Δ*C*_M_(urea), caused by the addition of crowding agents for the detailed discussion of our results.

### Unfolding of *Bs*CspB under dilute conditions and in crowded environments monitoring individual HN signals

In a next step, we have acquired two-dimensional heteronuclear ^1^H-^15^N HSQC NMR spectra of *Bs*CspB dependent on the concentration of urea in order to significantly increase the spectral resolution (Supplementary Fig. 3). In accordance with the analysis done for the acquisition of one-dimensional ^1^H NMR data (Fig. 2a, b), folding-to-unfolding transitions of amide protons comprising *Bs*CspB at a residue-by-residue level show a shift of *C*_M_ to a higher concentration of urea, when comparing crowded with non-crowded conditions (Supplementary Fig. 4a–c). The analysis of individual folding-to-unfolding transitions of *Bs*CspB was performed by using linear extrapolation of experimental data^36,37^ (see “Methods” section), since the approach for data analysis introduced by Santoro and Bolen^38^ is not indicated here as the baseline for native *Bs*CspB is poorly defined in folding-to-unfolding transitions (Supplementary Fig. 4a–c). It has been shown before that poorly defined baselines potentially falsify the thermodynamic analysis^39^. Thus, analyzing the transition region ranging between *c*^urea^ = 2 M and *c*^urea^ = 4 M for individual amino acids comprising *Bs*CspB omits efficiently the impact of the native baseline on the overall thermodynamic stability ${\Delta}G_{\text{N} \leftrightarrow \text{U}}^0$ and the folding cooperativity *m* (Supplementary Fig. 4d–f). In the present study, 53 amide proton folding-to-unfolding transitions characterizing *Bs*CspB could be entirely monitored in the absence of crowding agents yielding a sufficient height of cross-peaks corresponding to nonoverlapping, nonproline residues that have been unambiguously assigned (Supplementary Fig. 1b). In the presence of *c* = 120 g/L PEG1 or Dex20, 48 out of 53 individual folding-to-unfolding transitions could be monitored. The analysis of these folding-to-unfolding transitions by linear extrapolation has been performed for individual residues taking the three data sets for dilute conditions, *c* = 120 g/L PEG1 and *c* = 120 g/L Dex20 into account sharing the folding cooperativity *m* as global residue specific parameter during the fitting procedure (Supplementary Fig. 4d–f). The sum of all individual folding-to-unfolding transitions observed for NH cross-peaks gives rise to a mean normalized transition for each of the three different conditions, which have been probed here (Supplementary Fig. 5 and Supplementary Table 1). Data analysis by linear extrapolation results in *C*_M_ = 1.9 M for dilute conditions, $C_{\mathrm{M}}^{{\mathrm{dilute}}}$, and an increase by about Δ*C*_M_ = 0.5 M or Δ*C*_M_ = 0.6 M urea upon addition of *c* = 120 g/L Dex20 or PEG1, respectively (Supplementary Table 1). Again, this increase in thermodynamic stability of *Bs*CspB by about Δ*C*_M_ = 0.5 M urea in presence of MC is in accordance with folding-to-unfolding transitions based on the analysis of aliphatic protons applying one-dimensional ^1^H NMR spectroscopy (Fig. 2a, b). Note that the absolute value of *C*_M_ differs by about 0.7 M urea when comparing $C_{\mathrm{M}}^{{\mathrm{dilute}}}$ of one-dimensional ^1^H NMR analysis with mean $C_{\mathrm{M}}^{{\mathrm{dilute}}}$ obtained for native state amide protons derived from the analysis of two-dimensional ^1^H-^15^N HSQC spectra. This deviation can be attributed to the fact that in one-dimensional NMR spectra the change in the unfolded ensemble of *Bs*CspB is accounted for by integrating signals representing both native state as well as unfolded protein ensemble, respectively. In contrast, the analysis of folding-to-unfolding transitions of amide proton signals in two-dimensional HSQC NMR spectra focusses on the loss of the population of the native state only omitting information about the unfolded protein ensemble. In this respect, a shift of *C*_M_ to higher values can be seen when analyzing the increase in the population of the unfolded protein ensemble induced by an increasing concentration of urea monitored by two-dimensional ^1^H-^15^N HSQC spectra (Supplementary Fig. 6). Quantitatively, the mean in *C*_M_ is shifted to $C_{\mathrm{M}}^{{\mathrm{dilute}}}$ = 4.2 M monitoring 16 transitions that could be unambiguously assigned and analyzed throughout the course of protein unfolding (Supplementary Fig. 1c). The mean transition midpoint for the folding-to-unfolding transition of *Bs*CspB which is calculated by using folded as well as unfolded amide proton signals separately can be therefore specified with about 3.0 M urea, which is comparable to $C_{\mathrm{M}}^{{\mathrm{dilute}}}$ = 2.7 M obtained for one-dimensional ^1^H NMR analysis. Analyzing the unfolded protein ensemble directly, there is also a stabilization effect due to the presence of MC agents evident. This thermodynamic stabilization can be determined to about Δ*C*_M_ = 0.2 M and Δ*C*_M_ = 0.7 M for *c* = 120 g/L PEG1 or Dex20, respectively (Supplementary Fig. 6e, f and Supplementary Table 1). The difference in stabilization induced by the two different MC agents as seen here is not reflected in the analysis of the native state amide proton folding-to-unfolding transitions. Moreover, the standard deviations of the mean transition midpoints derived from the unfolded protein ensemble reach an overlapping range. Therefore, we must conclude that this deviation seen in the unfolded ensemble is not a difference in the extent of stabilization of the native state exerted by the two MC agents, but rather reflects a limit of accuracy in the determination of the stabilization by evaluation of the amide proton signals of the unfolded ensemble. Similar observations have been illuminated by protein folding studies of e.g., the protein barstar in comparing NMR spectroscopic data with fluorescence detected transitions^40,41^. Note that an additionally performed concentration dependent analysis of the folding-to-unfolding transition of *Bs*CspB conducted under dilute conditions does not show a significant difference in *C*_M_ (Supplementary Fig. 7). In addition, another control has shown that using either height or volume of cross-peaks for the analysis of individual folding-to-unfolding transitions leads to similar results in Δ*C*_M_ (Supplementary Fig. 8). Therefore, all cross-peaks acquired in two-dimensional heteronuclear NMR spectra in this study have been analyzed using peak height enabling a reliable readout of data even if cross-peaks are in close spectral proximity.

Monitoring folding-to-unfolding transitions based on NH cross-peaks further facilitates structurally resolved mapping of the individual thermodynamic stability at a residue-by-residue level under both dilute and MC conditions. First, mapping of individual folding-to-unfolding transition midpoints under dilute conditions to the primary sequence (Supplementary Fig. 9a) indicates that *Bs*CspB does not possess regions, which are characterized by a significant local increase or decrease in thermodynamic stability compared to the average confirming a two-state folding character of this protein. There are only few residues possessing *C*_M_^dilute^ values which do not lie within the range covered by the mean and one standard deviation. These are specifically Asn10, Ile18, Asp25, Phe27, Leu41, and Ala61 that possess a $C_{\mathrm{M}}^{{\mathrm{dilute}}}$ > 2.2 M whereas Gly4, Lys5, Ala32, Ile33, Lys39, Gln45, Ala46, and Glu50 own $C_{\mathrm{M}}^{{\mathrm{dilute}}}$ < 1.6 M (Supplementary Table 2). Mapping those residues on the structure of *Bs*CspB (Supplementary Fig. 10a) underlines that there is no structural relation present that would connect residues of raised or lowered stability. Next, addition of MC agents increases the residue specific thermodynamic stability equally using either PEG1 or Dex20 (Fig. 2c, d). Again, only single, structurally separated residues show a moderate difference to the range of *C*_M_ which is spanned by the mean and one standard deviation. Specifically, amino acids Gly4, Asn10, Ala32, Ile33, Leu41, and Ala61 mirror deviations larger than the mean plus one standard deviation in *C*_M_ (Supplementary Fig. 10b, c). However, these residues have been already identified analyzing *C*_M_^dilute^. The general thermodynamic stabilization by about Δ*C*_M_ = 0.5 M in the presence of either *c* = 120 g/L PEG1 or *c* = 120 g/L Dex20 is distributed over the entire primary sequence and is indifferent of surface or core regions, inherent properties of amino acids or the presence of secondary structural elements. This observation is in contrast to studies which have suggested that the effects of MC agents rely on the exposed surface area of the protein^42,43^, on a specific protein composition such as hydropathy or on the electrostatics of certain exposed residues^44,45^ due to direct interactions between MC agents and the protein under study. The results presented here underscore—at least for *Bs*CspB—the general character of thermodynamic stabilization when PEG1 or Dex20 molecules are present at a concentration of about *c* = 120 g/L. This increase in thermodynamic stability is independent of direct binding or specific chemical interactions between the MC agent and defined sites in the protein, which can be seen from the secondary structure and hydropathy notation presented in Fig. 2c, d. To sum up, the detailed analysis of amide proton signals does not provide evidences for the presence of local hot spots responsible for thermodynamic stabilization of *Bs*CspB and the extent of stabilization as induced by Dex20 and PEG1 is highly similar.

### Unfolding of *Bs*CspB under dilute conditions and in crowded environments monitoring individual CH, CH_2_, and CH_3_ signals

**Fig. 3:**
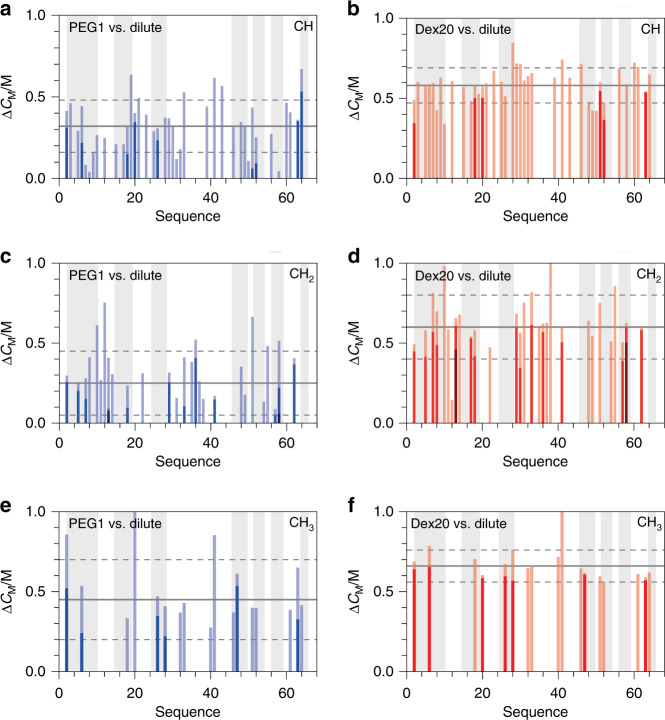
Thermodynamic stability of main chain and side chain atoms comprising *Bs*CspB monitored via following folding-to-unfolding transitions induced by urea. **a**–**f** Difference in the transition midpoint, Δ*C*_M_, of CH protons (**a**, **b**), CH_2_ protons (**c**, **d**), and CH_3_ protons (**e**, **f**) observed in two-dimensional ^1^H-^13^C HSQC NMR spectra comparing dilute conditions with the presence of *c* = 120 g/L PEG1 (colored in blue) or *c* = 120 g/L Dex20 (colored in red). Beta sheet regions according to PDB ID 1NMG are indicated by using a background colored in gray. The mean of Δ*C*_M_ is shown by the horizontal line (continuous mode) whereas the mean plus or minus one standard deviation of Δ*C*_M_ is represented by two additional horizontal lines (dotted mode). The presence of more than a single CH, CH_2_ or CH_3_ group per residue is highlighted by applying dark and bright mode of red or blue color, respectively. For the assignment of cross-peaks in two-dimensional ^1^H-^13^C HSQC NMR spectra see Supplementary Fig. 8.

The analysis of folding-to-unfolding transitions of resonance signals comprising *Bs*CspB has been further expanded by the acquisition of two-dimensional heteronuclear ^1^H-^13^C HSQC spectra in dependence on the concentration of urea (Supplementary Figs. 11 and 12). All folding-to-unfolding transitions of cross-peaks representing CH, CH_2_, and CH_3_ groups have been analyzed by linear extrapolation using both dilute and two MC conditions sharing the folding cooperativity *m* as global residue specific parameter during the fitting procedure (Supplementary Fig. 13a–f and Supplementary Tables 3–5). First, carbon bound protons comprising *Bs*CspB show an overall thermodynamic stability of about $C_{\mathrm{M}}^{{\mathrm{dilute}}}$ = 2.1 M urea which is in accordance with $C_{\mathrm{M}}^{{\mathrm{dilute}}}$ analyzed for cross-peaks representing NH groups (Supplementary Table 1 and Supplementary Fig. 9a, c). Secondly, for almost all amino acids, the residue specific value of $C_{\mathrm{M}}^{{\mathrm{dilute}}}$ determined for CH correlations lies within a range covered by one standard deviation of the mean. Thus, there are no structural regions present in *Bs*CspB which possess a thermodynamic stability that is significantly increased or decreased (Fig. 3a, b). Consequently, the two state folding character of *Bs*CspB^30^ monitored in equilibrium by using high-resolution NMR spectroscopy here is underlined by showing a well-defined midpoint of the folding-to-unfolding transitions for both individual NH and individual CH correlations. The few residues of *Bs*CspB whose methine groups show a deviation in $C_{\mathrm{M}}^{{\mathrm{dilute}}}$ from the mean value plus one standard deviation are Lys7, Ile18*, His29, Ile33*, Leu41*, Ala46, Ile51, Pro58, Val63, and Thr64. This list comprises three residues which have been identified before analyzing $C_{\mathrm{M}}^{{\mathrm{dilute}}}$ using NH cross-peaks and have been labeled by using “*” here. Thus, the independent and profound analysis of both NH and CH cross-peaks comprising *Bs*CspB dependent on the concentration of urea observed under dilute conditions does not illuminate a cluster of residues, which deviate in $C_{\mathrm{M}}^{{\mathrm{dilute}}}$ regarding the overall thermodynamic stability. Therefore, we conclude that deviations observed in $C_{\mathrm{M}}^{{\mathrm{dilute}}}$ are rather based on the very local flexibility and environment of nuclei comprising the amino acid under study.

Next, ^1^H-^13^C HSQC spectra of *Bs*CspB in presence of *c* = 120 g/L PEG1 or Dex20 have been acquired dependent on the concentration of urea. The addition of PEG1 or Dex20 is capable to increase the midpoint of folding-to-unfolding transitions of *Bs*CspB probed by individual CH signals on average to the same degree as seen for the analysis of NH signals (Supplementary Fig. 9b, d). Specifically, the presence of *c* = 120 g/L PEG1 leads to an increase in *C*_M_ of the folding-to-unfolding transition of *Bs*CspB of about 0.3 M urea whereas the same concentration of Dex20 increases the transition midpoint by about 0.6 M urea (Supplementary Table 1). Analyzing the folding-to-unfolding transitions of CH_2_ groups comprising *Bs*CspB leads quantitatively to the same result as seen for CH groups, illuminating an increase in *C*_M_ of 0.2 M urea in presence of *c* = 120 g/L PEG1, whereas the presence of the same amount of Dex20 increases *C*_M_ by about 0.6 M urea (Fig. 3c, d and Supplementary Table 4). The reliability of the experimental setup used for the determination of the thermodynamic stability of a protein at an atomic level outlined here is corroborated by the fact, that the increase in *C*_M_ caused by a crowded environment seen for one proton present in the CH_2_ group equals the extent of stabilization seen for the second proton in the same CH_2_ group. Even though the absolute values might be divergent, the gain in stability is precisely conserved (Supplementary Table 4). Last but not least, individual folding-to-unfolding transitions of CH_3_ groups comprising *Bs*CspB have been followed under dilute and MC conditions, respectively. In this regard, the midpoint *C*_M_ reporting on the unfolding of *Bs*CspB increases by about 0.5 M urea when *c* = 120 g/L PEG1 is present whereas *c* = 120 g/L Dex20 causes Δ*C*_M_ = 0.7 M urea (Fig. 3e, f and Supplementary Table 5). Note again that an increase in *C*_M_ observed for one methyl group comprising isoleucine, leucine or valine has been mirrored by the second methyl group present in the same residue (Supplementary Table 5).

### Analyzing potential interaction of urea with crowding agents and with *Bs*CspB being present in crowded environments

**Fig. 4:**
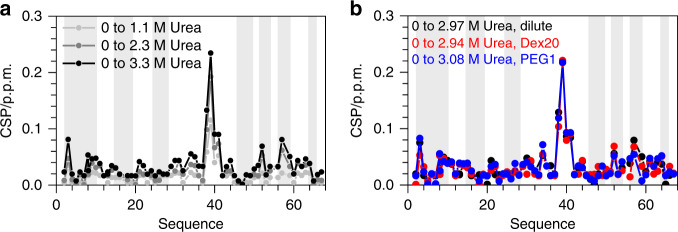
Chemical shift perturbations (CSPs) of NH cross-peaks comprising *Bs*CspB observed in two-dimensional ^1^H-^15^N HSQC NMR spectra induced by the presence of urea. **a** Changes in chemical shifts of ^1^H-^15^N cross-peaks comprising the native state of *Bs*CspB in presence of *c* = 1.1 M urea (light gray), *c* = 2.25 M urea (dark gray), and *c* = 3.26 M urea (black) compared to the absence of urea applying dilute conditions. **b** Changes in chemical shifts of ^1^H-^15^N cross-peaks comprising the native state of *Bs*CspB comparing *c* = 2.97 M with *c* = 0 M urea applying dilute conditions (colored in black), *c* = 2.94 M with *c* = 0 M urea using *c* = 120 g/L Dex20 (colored in red) and *c* = 3.08 M with *c* = 0 M urea using *c* = 120 g/L PEG1 (colored in blue). Beta sheet regions according to PDB ID 1NMG are indicated using a background colored in gray.

Further, we have analyzed the change in chemical shifts of NH cross-peaks present in ^1^H-^15^N HSQC NMR spectra to evaluate potential effects of urea on native *Bs*CspB which is supplemented with MC agents. Applying dilute conditions, the addition of urea to *Bs*CspB induces chemical shift perturbations (CSPs) of residues comprising predominantly the highly exposed loop ranging between Gly35 and Thr40 (Fig. 4a). The presence of crowding agents (*c* = 120 g/L PEG1 or Dex20) does neither qualitatively nor quantitatively alter the pattern of CSPs seen for *Bs*CspB under dilute conditions when comparing the absence with the presence of *c* = 3 M urea (Fig. 4b, Supplementary Fig. 14). Therefore, we conclude that the presence of crowding agents does not structurally perturb the folding-to-unfolding transitions of individual NH cross-peaks comprising *Bs*CspB, which have been induced by urea. In other words, the chemically induced unfolding in presence of PEG1 or Dex20 processes similar to the unfolding of *Bs*CspB under dilute conditions. Consequently, it can be reasonably anticipated that also urea-induced folding-to-unfolding transitions of CH, CH_2_, and CH_3_ cross-peaks are not structurally perturbed when comparing dilute with crowding conditions. Furthermore, one-dimensional proton NMR spectroscopy has been applied to monitor the potential impact of urea on crowding agents used in this study. Neither perturbations in chemical shifts nor modifications in line shape of resonance signals comprising PEG1 and Dex20 could be observed comparing *c*^urea^ = 0 M with *c*^urea^ = 6 M (Supplementary Fig. 15). This result indicates an inertness of PEG1 and Dex20 regarding urea even if present at a high concentration.

### Following hydrodynamic dimensions of *Bs*CspB upon unfolding

Finally, the experimental data set reporting on the folding-to-unfolding reaction of *Bs*CspB in absence and presence of crowding agents has been complemented by applying diffusion NMR methodology. The determination of the diffusion coefficient of *Bs*CspB dependent on the concentration of urea in absence and presence of *c* = 120 g/L Dex20 makes it possible to evaluate the folding-to-unfolding transition in terms of hydrodynamic dimensions, *r*_H_, taking native and unfolded conformations into account. First, applying dilute conditions cause a transition midpoint for the unfolding of *Bs*CspB of $C_{\mathrm{M}}^{{\mathrm{dilute}}}$ = 3.2 M urea taking averaged values of *r*_H_ into account (Supplementary Fig. 16). Analyzing the diffusion of *Bs*CspB in presence of *c* = 120 g/L Dex20 dependent on the concentration of urea increases *C*_M_ by about 0.5 M urea to *C*_M_ = 3.7 M (Supplementary Fig. 16). This increase in thermodynamic stability of *Bs*CspB obtained by analyzing the diffusion properties once *c* = 120 g/L Dex20 is present reproduces the results independently obtained by applying one-dimensional (Fig. 2b and Supplementary Table 1) and two-dimensional NMR spectroscopy (Fig. 2d and Supplementary Table 1). Notably, the hydrodynamic dimensions representing native and unfolded *Bs*CspB determined here possessing *r*_H,nativ_ = 15.7 Å and *r*_H,unf_ = 22.3 Å match very well with theoretically expected values of $r_{{\mathrm{H,nativ}}}^{{\mathrm{th}}} = 16.1\,{\mathrm{{\AA}}}$ and $r_{{\mathrm{H}},{\mathrm{unf}}}^{{\mathrm{th}}} = 24.3\,{\mathrm{{\AA}}}$ for a protein of this size^46^.

## Discussion

**Fig. 5:**
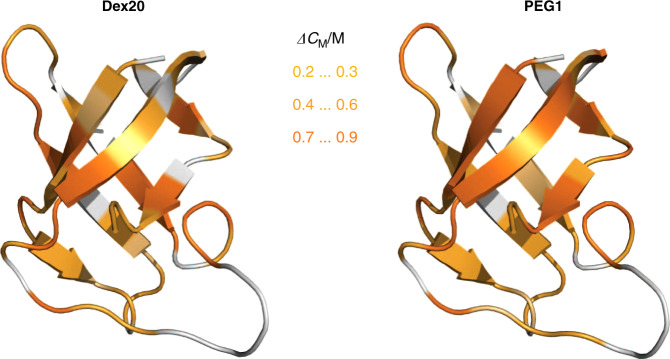
Increase of thermodynamic stability of *Bs*CspB due to the presence of *c* = 120 g/L MC. The increase in the transition midpoint, Δ*C*_M_, obtained for amide protons is mapped onto the three-dimensional structure of *Bs*CspB (PDB ID: 1NMG) applying Dex20 (left) or PEG1 (right). The following color code has been used: 0.2 M ≤ Δ*C*_M_ ≤ 0.3 M in light orange, 0.4 M ≤ Δ*C*_M_ ≤ 0.6 M in orange and 0.7 M ≤ Δ*C*_M_ ≤ 0.9 M in bright orange whereas residues colored in gray report on lack of experimental data. Numerical values for Δ*C*_M_ obtained under these experimental conditions can be found in Fig. 2c, d and Supplementary Table 2.

Applying NMR spectroscopy enabled the elucidation of the folding-to-unfolding transition of *Bs*CspB at highest resolution by observing cross-peaks belonging to backbone amide (Fig. 5), backbone carbon as well as side chain aliphatic protons dependent on the concentration of urea (Supplementary Fig. 17). Thereby, we have shown on all levels a cooperative effect in thermodynamic stabilization of *Bs*CspB due to adding of PEG1 or Dex20. We will discuss how this thermodynamic stabilization can be understood and focus for this reason on excluded volume theory, potential enthalpic contributions arising from crowding agents, potential changes in water structure or the hydration shell as well as potential effects due to the combination of different cosolutes.

Starting with Dex20, which can be modeled as a hard rod cosolute^47^, the thermodynamic stabilization of *Bs*CspB can be qualitatively understood by the excluded volume effect^47,48^. Dex20 acting as an inert macromolecule reduces the space available for *Bs*CspB in solution due to mutual exclusion and consequently *Bs*CspB favors the compact, native state compared to the extended unfolded ensemble^3^. Note that this purely entropic origin of the MC effect can be potentially accompanied by additional enthalpic contributions due to chemical interactions. Thus, it has been discussed that enthalpic contributions may add on to or counteract the entropic stabilization of the native state^27,47,49,50^. Focusing on PEG, the effects which have been reported for this cosolvent are highly diverse. Thus it has been shown that PEG can act as a denaturant^51^ leading to thermal destabilization^52^ whereas cosolute exclusion leads to purely entropic stabilization of protein complexes^53,54^. Moreover, early as well as recently performed experimental studies have reported both stabilizing and destabilizing effects of PEG, respectively^52,55^. Shortly speaking, PEG has shown so far to exert a stabilizing effect due to steric repulsion that could in some cases be diminished or overridden by specific or unspecific hydrophobic interactions with protein side chains supposedly depending on the properties of the protein under investigation^3,42,56^. There have been attempts to classify proteins by their hydrophobicity or primary sequence in order to predict negative or positive enthalpic contributions based on chemical interactions with the protein^43,57^. Quantitatively, the steric repulsion or excluded volume effect of PEG can be modeled in the same way as for Dex20 since PEG conformations in solution have been described as low density random coils^20^ that can be sufficiently modeled as hard particles^58^.

Secondly, our results may indicate that in the context of MC and cosolute properties protein stabilization caused by a crowded environment might take place indirectly by changes in the water structure namely in the hydrogen bonding network of the solution^59,60^. Consequently, a perturbation of the pure water hydrogen bonding network might favor or disfavor the hydration of either the native or the unfolded protein species, if the thermodynamic stabilization of the protein under study gets influenced by changes in the water structure. In the present study, it is unlikely that the addition of PEG significantly changes the hydrogen bonding network composed of water and urea as PEG does not comprise a wealth of hydroxy groups being able to interact with urea. In the case of dextran though, the amino groups comprising urea may accept protons not only from water but also to a significant amount from Dex20 molecules which can be seen in the difference in pH value probing a pure water-urea solution (pH = 8.4 at *c*^urea^ = 6 M) compared to a water-urea—Dex20 solution (pH = 7.4 at *c*^urea^ = 6 M and *c*^Dex20^ = 100 g/L). This decrease in pH value mirrors the reduced number of hydroxide ions which are set free once the amino groups comprising urea get protonated. In other words, urea potentially accepts protons from hydroxy groups in a solution which contains a polyol like Dex20. Thus, Dex20 is possibly involved in changes of the water structure and may influence the hydrogen bonding network. On the contrary, PEG does not perturb the water structure to this extent as the value of a solution containing water, urea and PEG1 (pH = 8.1 at *c*^urea^ = 6 M and *c*^PEG1^ = 100 g/L) does not significantly differ to a solution containing water and urea only (pH = 8.4 at *c*^urea^ = 6 M). Hence, we conclude that it is not very likely that PEG1 or Dex20 manipulate the structure of water and the hydration shell which is spanned around *Bs*CspB, as they inherently possess different effects on water structure, but influence the thermodynamic stability of *Bs*CspB to the same extent. We acknowledge that the extent of entropic repulsion—thus the size of the excluded volume caused by the presence of MC—depends on the thickness of the hydration shell. But for *Bs*CspB being dissolved in Dex20 or PEG1, the impact on water structure and the hydration shell, if even present, has a negligible effect on the thermodynamic stability as our results show.

Moving on, it has been shown that destabilizing agents such as urea and stabilizing cosolutes can interact with each other in a way that diminishes individual contributions of the cosolutes^61^. Does the denaturation strength and the mechanism urea possesses regarding protein unfolding change once MC agents are present? Mechanistically, urea denatures proteins by changes in the hydration shell accompanied by a direct contact with the protein surface^62,63^. A class of small molecules, so-called “protective osmolytes”, stabilizes proteins against unfolding caused by urea. In this way, TMAO, inositol, sugars or polyols in general^61,64,65^ represent potent protective osmolytes that show stabilizing effects which mainly originate from interactions with the protein backbone^66^. Is Dex20 possibly capable of interactions, which are typically seen for small polyols? Even though dextran is widely accepted as an inert MC agent, it has also been shown that it is able to interact with proteins and thereby contributes a non-negligible enthalpic component^42^. For the model protein *Bs*CspB studied here, the increase in thermodynamic stability seen for the protein backbone is the same for both crowding agents used in our experimental setup. Consequently, the stabilizing effects caused by the possibly affected hydrogen bonding network are either identical—which is unlikely as PEG1 and Dex20 differ significantly in polarity—or they are negligible. The present difference in polarity comparing PEG1 with Dex20 cannot be seen in an interaction with the protein backbone which gets inherently stabilized by hydrogen bonds. This also underlines that there is no direct interaction between amide protons and MC agents. One can assume that such an interaction would be dependent on the polarity of MC agents and would thus have to differ between PEG1 and Dex20. But, the folding-to-unfolding transitions which have been monitored here by applying one-dimensional NMR spectroscopy as well as by two-dimensional ^1^H-^15^N HSQCs report no differences in the thermodynamic stabilization of *Bs*CspB, which has been induced by addition of PEG1 or Dex20, two crowding agents that differ in their hydropathicity. The analysis of carbon bound protons using two-dimensional ^1^H-^13^C HSQC spectra shows minor differences in the increase of the thermodynamic stability induced by the addition of Dex20 compared to PEG1, as the residue averaged mean Δ*C*_M_ is 0.4 M urea for PEG1 but 0.6 M urea for Dex20 (Supplementary Table 1). This may indicate a potential influence of the difference in the polarity of Dex20 and PEG1, but could also result from possible destabilizing hydrophobic interactions of PEG1. To the best of our knowledge, any kind of destabilizing interactions having an impact on folding-to-unfolding transitions of CH groups must also be monitored for the atom, which is next to it which is in this case the amide proton. Since this destabilizing behavior is not present in ^1^H-^15^N HSQC derived transitions, we conclude that this minor difference in mean stabilization seen for carbon bound protons comparing the addition of Dex20 with the addition of PEG1 is negligible. However, what holds as a reason that an enthalpic contribution (if existent) for *Bs*CspB can only be of negligible or equal extent for PEG1 and Dex20 as no hydrophobic interactions can be detected whereas a couple of different proteins have shown such a contribution^67^? This feature is potentially based on the low hydrophobicity *Bs*CspB owns and its polyanionic net charge, estimated as −5.8 at pH 7. Note that it has been shown that the presence of charged residues increases the repulsive interaction of proteins with PEG, especially the presence of anionic charges enables to diminish hydrophobic interactions of proteins with PEG even more pronounced than cationic charges do^57^.

Taken all results together, we postulate that the increase in thermodynamic stability observed for *Bs*CspB here is purely entropically driven. We propose that the effect of excluded volume is dominantly responsible for the increase in thermodynamic stability of *Bs*CspB taking crowding agents covering a range of molecular weight between 1 to 20 kDa and concentrations of about 120 g/L into account. Interestingly, the pronounced difference in molecular weight of crowding agents used here does not change the magnitude of the stabilization seen for *Bs*CspB. This demonstrates that a crowded environment which is composed of molecules possessing a molecular weight of about 1 kDa only procures an excluded volume effect regarding a protein of about 7 kDa in molecular weight (further discussion in the Supplementary Information).

Motivated by the lack of highly resolved experimental data from systematic bottom-up studies that are required to complement the theoretical discussion of MC^50^, this work contributes essential insights into the excluded volume induced stabilization at highest resolution possible. Probing the impact a crowded environment has on the thermodynamic stability of *Bs*CspB at the level of ^1^H, ^15^N, and ^13^C one-dimensional and two-dimensional NMR spectroscopy enables to illuminate the dominance of entropic stabilization over enthalpic contributions at every level of resolution independent of the local site of detection. This finding narrows the gap between theoretical predictions and experimental proofs regarding the estimation of crowding effects on proteins.

It thereby underlines the high relevance of intracellular crowding, which affects essentially all cellular processes in vivo, and contributes to the evolution of a precise understanding of cellular protein action and interaction. Thus, a boost in following highly resolved in-cell studies enabling investigations performed also at atomic resolution can be proposed. Finally, we believe that due to the systematic experimental setup and comprehensive data analysis applied here a generalization of the results to proteins showing folding characteristics which are comparable to *Bs*CspB is feasible.

## Methods

### Protein expression and purification

Protein expression and purification occurred as described^30,68^ with minor modifications. Buffer exchange and sample concentration was carried out using centrifugal filters (Amicon, Merck). Protein concentration was determined using an absorbance spectrometer; ε^280nm^ = 5800 M^−1^ cm^−1^ ^30^, aliquots were shock frozen and stored at −80 °C. Expression of ^15^N and ^13^C labeled *Bs*CspB occurred in M9 minimal medium using ^15^N ammonium chloride (Cortecnet) and ^13^C glucose (Cambridge Isotope Laboratories) as nitrogen and carbon sources.

### Sample preparation

Samples were prepared from stock solutions of Urea (ultra pure, MP), sodium cacodylate (Alfa Aesar) and the following crowding agents: Dextran T20 (Dex20) of a molecular weight of 20 kDa (Pharmacosmos), polyethylene glycol (PEG) of a molecular weight of 1 kDa (Roth). Dilute conditions refer to 20 mM sodium cacodylate at pH 7 and *T* = 298 K.

### NMR measurements

1D ^1^H NMR spectra, 2D ^1^H-^15^N HSQC-TROSY and 2D ^1^H-^13^C HSQC spectra were recorded for a *Bs*CspB solution (95% H_2_O/5% D_2_O, 20 mM sodium cacodylate/HCl, pH 7.0, containing either no crowder, 120 g/L Dex20, or 120 g/L PEG1) on a Bruker 600 MHz spectrometer with a cryogenic probe head. Folding-to-unfolding transitions of *Bs*CspB have been monitored at *c*^*Bs*CspB^ = 1.09 mM (dilute and 120 g/L Dex20 condition), 0.8 mM (120 g/L PEG1 condition), 0.25 mM (diffusion measurement). An additional folding-to-unfolding transition of *Bs*CspB has been monitored under dilute conditions at c^*Bs*CspB^ = 0.03 mM. Water suppression occurred using presaturation and a Watergate pulse sequence^69^. Pseudo 2D diffusion spectra of a 250 µM *Bs*CspB solution (95% H_2_O/5% D_2_O, 20 mM sodium cacodylate/HCl; pH 7.0, with and without 120 g/L Dex20) were acquired on a Bruker 800 MHz spectrometer operated with a cryogenic probe head dependent on the applied gradient. Due to high molecular concentration of buffer molecules in crowded and denatured samples, further signal suppression was executed via presaturation of buffer specific frequencies.

### Data analysis

^1^H NMR transition data for chemical unfolding was evaluated using integrals of 1D spectra in the aliphatic region, readout occurred with the software TopSpin (Bruker). Integration of signals typical for the native conformation occurred from 0.59 to 0.14 ppm and for the increase in the unfolded population, signals from 0.697 to 1.064 ppm were integrated. See ref. ^23^ for details. For data regression a thermodynamic two state model was employed as described by Szyperski et al.^70^. For chemical unfolding, qualitative analysis of two-dimensional ^1^H-^15^N HSQC spectra and ^1^H-^13^C HSQC spectra occurred via readout of peak height comprising all NH and all nonoverlapping CH, CH_2_, and CH_3_ cross-peaks. The standard deviation of Δ*C*_M_, ΔΔ*C*_M_, has been calculated for *n* residues according to ${\Delta}{\Delta}C_{\mathrm{M}} = \sqrt {\frac{{\mathop {\sum }\nolimits_{i = 1}^{\mathrm{n}} (C_{\mathrm{M}}^{\mathrm{i}} - {\Delta}C_{\mathrm{M}})^2}}{{n - 1}}}$ in which $C_{\mathrm{M}}^{\mathrm{i}}$ represents the midpoint of residue specific folding-to-unfolding transition.

For diffusion analysis, pseudo-2D spectra were processed using TopSpin. For baseline correction and integration Mestrenova 14 (Mestrelab Research) was used. Qualitative analysis of 2D spectra focused on the change in ^1^H and ^15^N chemical shifts. Both axes were referenced using TMSP and ^15^NH_4_Cl reference probes in the corresponding buffer / crowder concentration. All two-dimensional HSQC spectra were processed with the software NMR Pipe^71^, readout of individual peak height and chemical shift occurred using the software NMRView^72^. Perturbations of chemical shifts, CSPs, reporting on NH cross-peaks as acquired in two-dimensional ^1^H-^15^N HSQC NMR spectra were calculated according to Grzesiek et al.^73^. All folding-to-unfolding transitions were analyzed using the software Origin. For data regression of global chemical unfolding (detection of one-dimensional NMR spectra) the model of Santoro and Bolen was used^38^. For data regression of individual chemical unfolding transitions comprising single HN, CH, CH_2_, and CH_3_ transitions, linear extrapolation of the transition region was used^37^. NMR diffusion experiments were performed using a pulse sequence comprising a stimulated echo assisted by bipolar gradients^74^, *G*, employing a diffusion time, Δ, of 120 ms and gradient length, *δ*, of 5 ms along the *z*-axis. Gradients were calibrated as described in the ref. ^75^. Three different gradient strengths have been repeated three times each to estimate the experimental error. Integrals for proton signals, *I*, were determined for *Bs*CspB in the spectral range between 9.55 and 6.50 ppm and used for calculation of the diffusion coefficient, *D*:

1 $$I\left( G \right)\,=\,I\left( 0 \right){\mathrm{exp}}( - G^2\gamma ^2\delta ^2D({\Delta} - \delta /3)),$$

where *γ* is the gyromagnetic ratio of protons. Hydrodynamic radii, *R*_H_, were calculated using the respective diffusion coefficients of TMSP in order to account for the microviscosity of the respective sample. Microviscosities were calculated according to the Einstein–Stokes equation using TMSP with *r*_H_ = 3.4 Å contained in the respective sample. The hydrodynamic radius for TMSP was estimated by using dioxane with a known *r*_H_ = 2.12 Å as reported in^46^.

### Linear extrapolation of experimental data

For data regression, heights of cross-peaks were normalized to the highest value found for the respective resonance and were reported as normalized values. The normalized values were set equal to fraction native of *Bs*CspB present in solution and the derived Δ*G* values were plotted over denaturant concentration. Linear regression was performed in the transition region, including all data points in between 2 and 4 M urea, setting the cooperativity *m* as a residue specific parameter shared for the respective signal using the three conditions “dilute”, *c* = 120 g/L PEG1 and *c* = 120 g/L Dex20.

### Reporting summary

Further information on research design is available in the Nature Research Reporting Summary linked to this article.

## Supplementary information

Supplementary Information

Peer Review

Reporting Summary

## Data Availability

The data that support the findings of this study are available from the corresponding author upon reasonable request. Source data are provided with this paper.

## Source data

Source Data

## References

[1] Cayley S, Lewis BA, Guttman HJ, Record MT (1991). Characterization of the cytoplasm of Escherichia coli K-12 as a function of external osmolarity: implications for protein-DNA interactions in vivo. J. Mol. Biol..

[2] Zimmerman SB, Trach SO (1991). Estimation of macromolecule concentrations and excluded volume effects for the cytoplasm of Escherichia coli. J. Mol. Biol..

[3] Zhou HX, Rivas G, Minton AP (2008). Macromolecular crowding and confinement: biochemical, biophysical, and potential physiological consequences. Annu. Rev. Biophys..

[4] Schnell S, Turner TE (2004). Reaction kinetics in intracellular environments with macromolecular crowding: simulations and rate laws. Prog. Biophys. Mol. Biol..

[5] Wang Q, Zhuravleva A, Gierasch LM (2011). Exploring weak, transient protein–protein interactions in crowded in vivo environments by in-cell nuclear magnetic resonance spectroscopy. Biochemistry.

[6] Han J, Herzfeld J (1993). Macromolecular diffusion in crowded solutions. Biophys. J..

[7] Ellis RJ, Minton AP (2003). Cell biology: join the crowd. Nature.

[8] Sarkar M, Li C, Pielak GJ (2013). Soft interactions and crowding. Biophys. Rev..

[9] Smith AE, Zhou LZ, Gorensek AH, Senske M, Pielak GJ (2016). In-cell thermodynamics and a new role for protein surfaces. Proc. Natl Acad. Sci. USA.

[10] Dedmon MM, Patel CN, Young GB, Pielak GJ (2002). FlgM gains structure in living cells. Proc. Natl Acad. Sci. USA.

[11] Luh LM (2013). Molecular crowding drives active Pin1 into nonspecific complexes with endogenous proteins prior to substrate recognition. J. Am. Chem. Soc..

[12] Chen E (2012). Effects of macromolecular crowding on burst phase kinetics of cytochrome c folding. Biochemistry.

[13] Echeverria C, Kapral R (2012). Molecular crowding and protein enzymatic dynamics. Phys. Chem. Chem. Phys..

[14] Bismuto E (2002). Effect of molecular confinement on internal enzyme dynamics: Frequency domain fluorometry and molecular dynamics simulation studies. Biopolymers.

[15] Akabayov B, Akabayov SR, Lee S-J, Wagner G, Richardson CC (2013). Impact of macromolecular crowding on DNA replication. Nat. Commun..

[16] Dupuis NF, Holmstrom ED, Nesbitt DJ (2014). Molecular-crowding effects on single-molecule RNA folding/unfolding thermodynamics and kinetics. Proc. Natl Acad. Sci. USA.

[17] Desai R, Kilburn D, Lee H-T, Woodson SA (2014). Increased ribozyme activity in crowded solutions. J. Biol. Chem..

[18] Kulp DT, Herzfeld J (1995). Crowding-induced organization of cytoskeletal elements. III. Spontaneous bundling and sorting of self-assembled filaments with different flexibilities. Biophys. Chem..

[19] Breydo L (2014). The crowd you’re in with: effects of different types of crowding agents on protein aggregation. Biochim. Biophys. Acta.

[20] Shtilerman MD, Ding TT, Lansbury PT (2002). Molecular crowding accelerates fibrillization of α-synuclein: could an increase in the cytoplasmic protein concentration induce Parkinson’s disease?. Biochemistry.

[21] Luo X-D, Kong F-L, Dang H-B, Chen J, Liang Y (2016). Macromolecular crowding favors the fibrillization of β2-microglobulin by accelerating the nucleation step and inhibiting fibril disassembly. Biochim. Biophys. Acta.

[22] Stagg L, Christiansen A, Wittung-Stafshede P (2011). Macromolecular crowding tunes folding landscape of parallel alpha/beta protein, apoflavodoxin. J. Am. Chem. Soc..

[23] Köhn B, Kovermann M (2019). Macromolecular crowding tunes protein stability by manipulating solvent accessibility. ChemBioChem.

[24] Soranno A (2014). Single-molecule spectroscopy reveals polymer effects of disordered proteins in crowded environments. Proc. Natl Acad. Sci. USA.

[25] Mikaelsson T, Ådén J, Johansson LBÅ, Wittung-Stafshede P (2013). Direct observation of protein unfolded state compaction in the presence of macromolecular crowding. Biophys. J..

[26] Danielsson J (2015). Thermodynamics of protein destabilization in live cells. Proc. Natl Acad. Sci. USA.

[27] Rivas G, Minton AP (2016). Macromolecular crowding in vitro, in vivo, and in between. Trends Biochem. Sci..

[28] Schindelin H, Marahiel MA, Heinemann U (1993). Universal nucleic acid-binding domain revealed by crystal structure of the B. subtilis major cold-shock protein. Nature.

[29] Schnuchel A (1993). Structure in solution of the major cold-shock protein from Bacillus subtilis. Nature.

[30] Schindler T, Herrler M, Marahiel MA, Schmid FX (1995). Extremely rapid protein folding in the absence of intermediates. Nat. Struct. Biol..

[31] Schindler T, Schmid FX (1996). Thermodynamic properties of an extremely rapid protein folding reaction. Biochemistry.

[32] Jacob M (1999). Microsecond folding of the cold shock protein measured by a pressure-jump technique. Biochemistry.

[33] Jacob M, Schindler T, Balbach J, Schmid FX (1997). Diffusion control in an elementary protein folding reaction. Proc. Natl Acad. Sci. USA.

[34] Minton AP, Wilf J (1981). Effect of macromolecular crowding upon the structure and function of an enzyme: glyceraldehyde-3-phosphate dehydrogenase. Biochemistry.

[35] Christiansen A, Wang Q, Cheung MS, Wittung-Stafshede P (2013). Effects of macromolecular crowding agents on protein folding in vitro and in silico. Biophys. Rev..

[36] Pace, C. N. *Methods in Enzymology* Vol. 131, 266–280 (Academic Press, Cambridge, 1986).

[37] Greene RF, Pace CN (1974). Urea and guanidine hydrochloride denaturation of ribonuclease, lysozyme, α-chymotrypsin, and β-lactoglobulin. J. Biol. Chem..

[38] Santoro MM, Bolen DW (1988). Unfolding free energy changes determined by the linear extrapolation method. 1. Unfolding of phenylmethanesulfonyl alpha-chymotrypsin using different denaturants. Biochemistry.

[39] Haupt C, Weininger U, Kovermann M, Balbach J (2011). Local and coupled thermodynamic stability of the two-domain and bifunctional enzyme SlyD from *Escherichia coli*. Biochemistry.

[40] Hofmann H (2009). Fast amide proton exchange reveals close relation between native-state dynamics and unfolding kinetics. J. Am. Chem. Soc..

[41] Hofmann H, Golbik RP, Ott M, Hübner CG, Ulbrich-Hofmann R (2008). Coulomb forces control the density of the collapsed unfolded State of Barstar. J. Mol. Biol..

[42] Jiao M, Li H-T, Chen J, Minton AP, Liang Y (2010). Attractive protein-polymer interactions markedly alter the effect of macromolecular crowding on protein association equilibria. Biophys. J..

[43] Shkel IA, Knowles DB, Record MT (2015). Separating chemical and excluded volume interactions of polyethylene glycols with native proteins: comparison with PEG effects on DNA helix formation. Biopolymers.

[44] Lee JC, Lee LL (1981). Preferential solvent interactions between proteins and polyethylene glycols. J. Biol. Chem..

[45] Crowley PB, Keith B, Jimmy M (2008). NMR spectroscopy reveals cytochrome c-poly(ethylene glycol) interactions. ChemBioChem.

[46] Wilkins DK (1999). Hydrodynamic radii of native and denatured proteins measured by pulse field gradient NMR techniques. Biochemistry.

[47] Minton AP (2005). Models for excluded volume interaction between an unfolded protein and rigid macromolecular cosolutes: macromolecular crowding and protein stability revisited. Biophys. J..

[48] Minton AP (2000). Effect of a concentrated inert macromolecular cosolute on the stability of a globular protein with respect to denaturation by heat and by chaotropes: a statistical-thermodynamic model. Biophys. J..

[49] Minton AP (1981). Excluded volume as a determinant of macromolecular structure and reactivity. Biopolymers.

[50] Rivas G, Minton AP (2018). Toward an understanding of biochemical equilibria within living cells. Biophys. Rev..

[51] Arakawa T, Bhat R, Timasheff SN (1990). Why preferential hydration does not always stabilize the native structure of globular proteins. Biochemistry.

[52] Lee LLY, Lee JC (1987). Thermal stability of proteins in the presence of poly(ethylene glycols). Biochemistry.

[53] Reddy, M. K., Weitzel, S. E., Daube, S. S., Jarvis, T. C. & von Hippel, P. H. *Methods in Enzymology* Vol. 262 466–476 (Academic Press, Cambridge, 1995). 10.1016/0076-6879(95)62038-9 8594371

[54] Jarvis TC, Ring DM, Daube SS, von Hippel PH (1990). “Macromolecular crowding”: thermodynamic consequences for protein–protein interactions within the T4 DNA replication complex. J. Biol. Chem..

[55] Tellam RL, Sculley MJ, Nichol LW, Wills PR (1983). The influence of poly(ethylene glycol) 6000 on the properties of skeletal-muscle actin. Biochem. J..

[56] Mukherjee SK, Gautam S, Biswas S, Kundu J, Chowdhury PK (2015). Do macromolecular crowding agents exert only an excluded volume effect? A protein solvation study. J. Phys. Chem. B.

[57] Knowles DB (2015). Chemical interactions of polyethylene glycols (PEGs) and glycerol with protein functional groups: applications to effects of PEG and glycerol on protein processes. Biochemistry.

[58] Zimmerman SB, Minton AP (1993). Macromolecular crowding: biochemical, biophysical, and physiological consequences. Annu. Rev. Biophys. Biomol. Struct..

[59] Eggers DK, Valentine JS (2001). Crowding and hydration effects on protein conformation: a study with sol-gel encapsulated proteins. J. Mol. Biol..

[60] Panuszko A, Bruździak P, Kaczkowska E, Stangret J (2016). General mechanism of Osmolytes’ influence on protein stability irrespective of the type of osmolyte cosolvent. J. Phys. Chem. B.

[61] Holthauzen LMF, Bolen DW (2007). Mixed osmolytes: the degree to which one osmolyte affects the protein stabilizing ability of another. Protein Sci..

[62] Bennion BJ, Daggett V (2003). The molecular basis for the chemical denaturation of proteins by urea. Proc. Natl Acad. Sci. USA.

[63] Lim WK, Rösgen J, Englander SW (2009). Urea, but not guanidinium, destabilizes proteins by forming hydrogen bonds to the peptide group. Proc. Natl Acad. Sci. USA.

[64] Yancey PH, Clark ME, Hand SC, Bowlus RD, Somero GN (1982). Living with water stress: evolution of osmolyte systems. Science.

[65] Manning MC, Chou DK, Murphy BM, Payne RW, Katayama DS (2010). Stability of protein pharmaceuticals: an update. Pharm. Res..

[66] Liu Y, Bolen DW (1995). The peptide backbone plays a dominant role in protein stabilization by naturally occurring osmolytes. Biochemistry.

[67] Wang Y, Sarkar M, Smith AE, Krois AS, Pielak GJ (2012). Macromolecular crowding and protein stability. J. Am. Chem. Soc..

[68] Sachs R, Max KE, Heinemann U, Balbach J (2012). RNA single strands bind to a conserved surface of the major cold shock protein in crystals and solution. RNA.

[69] Piotto M, Saudek V, Sklenář V (1992). Gradient-tailored excitation for single-quantum NMR spectroscopy of aqueous solutions. J. Biomol. NMR.

[70] Szyperski T, Mills JL, Perl D, Balbach J (2006). Combined NMR-observation of cold denaturation in supercooled water and heat denaturation enables accurate measurement of ΔC p of protein unfolding. Eur. Biophys. J..

[71] Delaglio F (1995). NMRPipe: a multidimensional spectral processing system based on UNIX pipes. J. Biomol. NMR.

[72] Kirby NI, DeRose EF, London RE, Mueller GA (2004). NvAssign: protein NMR spectral assignment with NMRView. Bioinformatics.

[73] Grzesiek S, Stahl SJ, Wingfield PT, Bax A (1996). The CD4 determinant for downregulation by HIV-1 nef directly binds to nef. mapping of the nef binding surface by NMR. Biochemistry.

[74] Jones JA, Wilkins DK, Smith LJ, Dobson CM (1997). Characterisation of protein unfolding by NMR diffusion measurements. J. Biomol. NMR.

[75] Berger, S. & Braun, S. *200 and more NMR Experiments. A Practical Course*. (Wiley, Hoboken, 2004).

[76] Kyte J, Doolittle RF (1982). A simple method for displaying the hydropathic character of a protein. J. Mol. Biol..

[77] Gasteiger, E. et al. *Protein Identification and Analysis Tools on the ExPASy Server*. 571–607 (Humana Press, Totowa, 2005). 10.1385/1-59259-584-7:531 10027275

