# Supplementary material for: All atom insights into the impact of crowded environments on protein stability by NMR spectroscopy
Source: Nat Commun. 2020 Nov 13;11:5760. doi: 10.1038/s41467-020-19616-w (PMC7666220; doi:10.1038/s41467-020-19616-w)
Supplement: Supplementary file 1 — Supplementary Information [file 41467_2020_19616_MOESM1_ESM.pdf]

Supplementary Information

All atom insights into the impact of crowded environments on protein stability by NMR spectroscopy

Birgit Köhn[1,2] and Michael Kovermann[1,2,*]

[1]Department of Chemistry, University of Konstanz, Universitätsstrasse. 10, 78457 Konstanz, Germany

[2]Konstanz Research School Chemical Biology KoRS-CB, University of Konstanz, Universitätsstrasse. 10, 78457 Konstanz, Germany

[*]Michael Kovermann, Department of Chemistry, University of Konstanz, Universitätsstrasse. 10, 78457 Konstanz, Germany, Phone number: +49 7531 88 3801, Email: michael.kovermann@uni-konstanz.de.

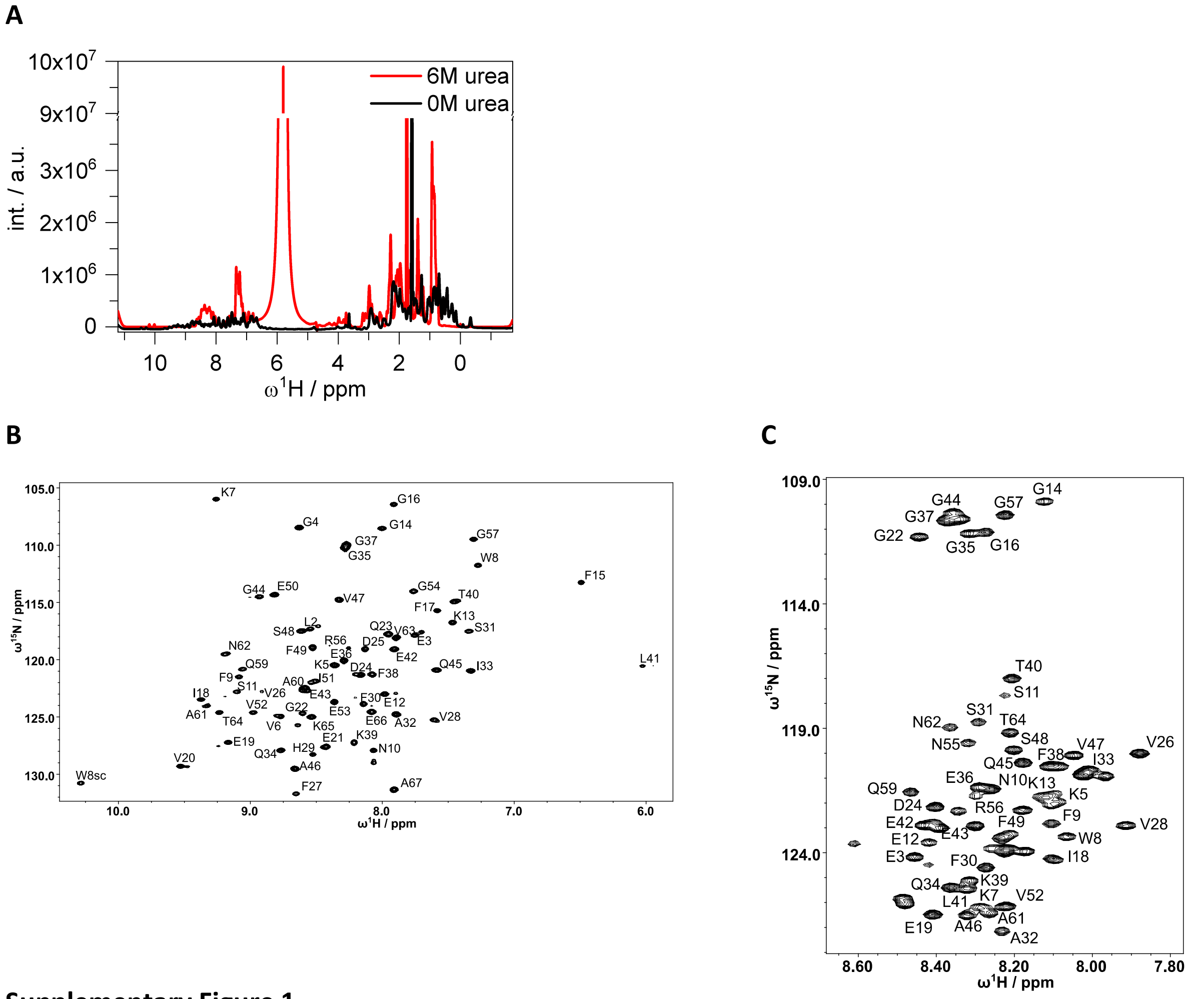

Supplementary Figure 1

One- and two-dimensional NMR spectra of native and chemically unfolded *Bs*CspB. (*A*) One-dimensional proton NMR spectrum of *Bs*CspB in the absence (colored in black) and in presence of $c = 6.05$ M urea (colored in red). Additional one-dimensional proton NMR spectra of *Bs*CspB acquired at other concentrations of urea than $c = 0$ M and $c = 6.05$ M are shown in Supplementary Figure 2. (*B*) Two-dimensional ^{1}H-^{15}N HSQC backbone assignment comprising 63 resonances representing the native state of *Bs*CspB in absence of urea using the one letter code of amino acids followed by the position in the primary sequence. (*C*) For the unfolded ensemble of *Bs*CspB (presence of $c = 6.05$ M urea), 44 cross-peaks have been unambiguously assigned in the two-dimensional ^{1}H-^{15}N HSQC spectrum. All NMR spectra have been acquired at $T = 298$ K.

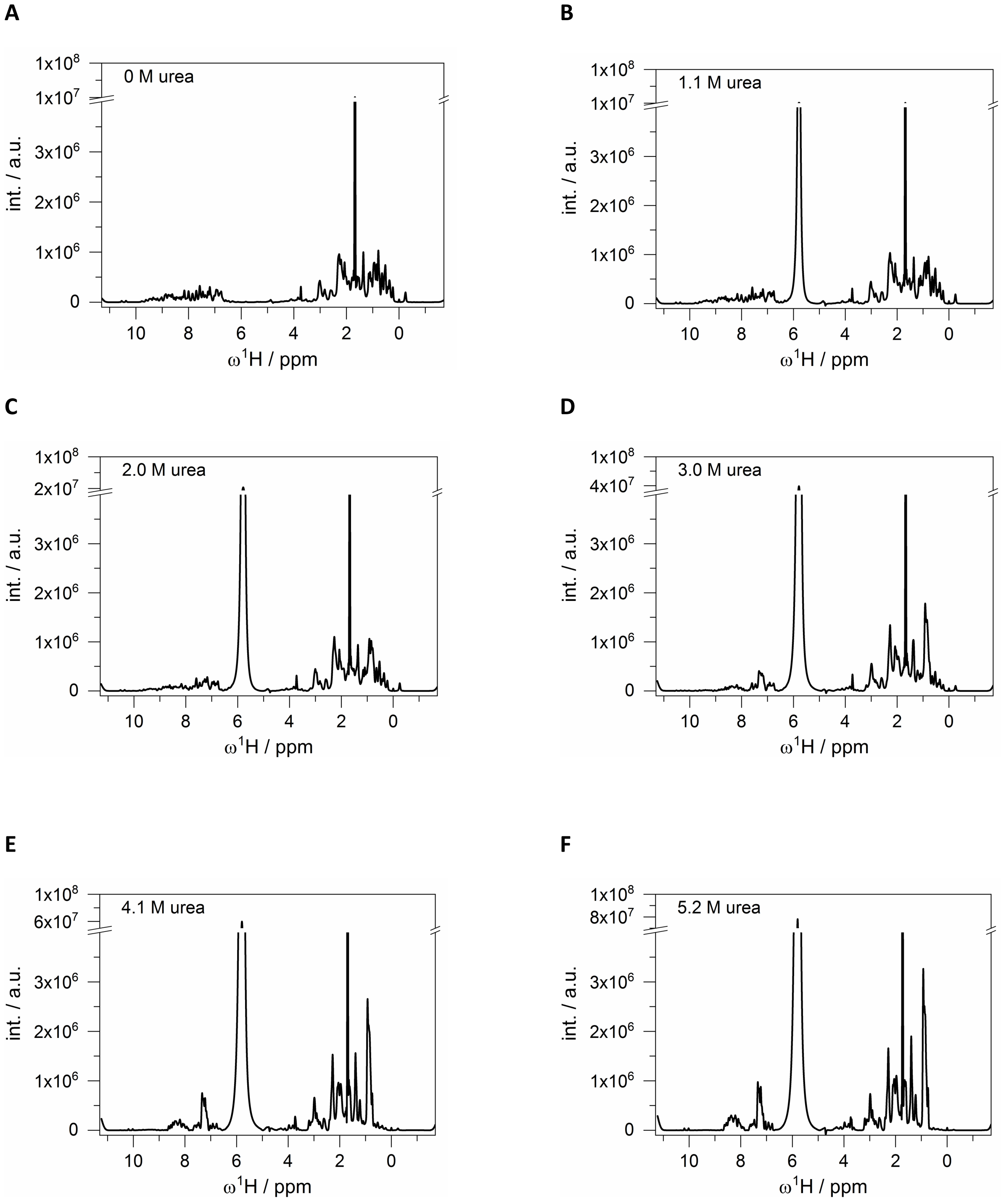
A
B
C
D
E
F
0 M urea
1.1 M urea
2.0 M urea
3.0 M urea
4.1 M urea
5.2 M urea
int. / a.u.
ω1H / ppm
1x10^8
1x10^7
2x10^7
4x10^7
6x10^7
8x10^7
3x10^6
2x10^6
1x10^6
0
10
8
6
4
2

G

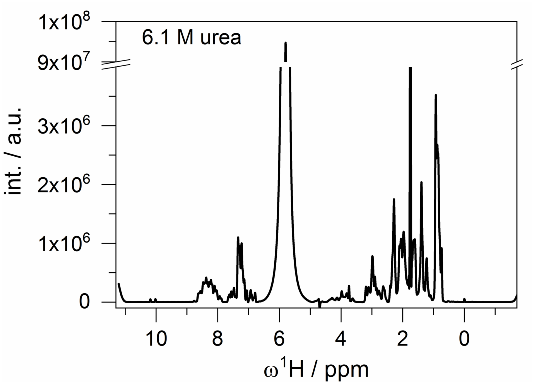

Supplementary Figure 2

One-dimensional proton NMR spectra of *Bs*CspB acquired under dilute conditions in presence of varying amounts of urea: c^{urea} = 0 M (*A*), c^{urea} = 1.1 M urea (*B*), c^{urea} = 2.0 M urea (*C*), c^{urea} = 3.0 M urea (*D*), c^{urea} = 4.1 M urea (*E*), c^{urea} = 5.2 M urea (*F*) and c^{urea} = 6.1 M urea (*G*). All spectra are referenced using the proton resonance signal of TMSP. Resonance signals comprising the range of chemical shifts 0.14 ppm $\leq \omega^{1H} \leq$ 0.59 ppm report on the native state of *Bs*CspB whereas 0.697 ppm $\leq \omega^{1H} \leq$ 1.064 ppm reports on both the native state as well as the unfolded protein ensemble. Thus, the fraction of native protein, f_n, can be reliably calculated as presented in Figure 2*A*, *B* and reported in Supplementary Table 1.

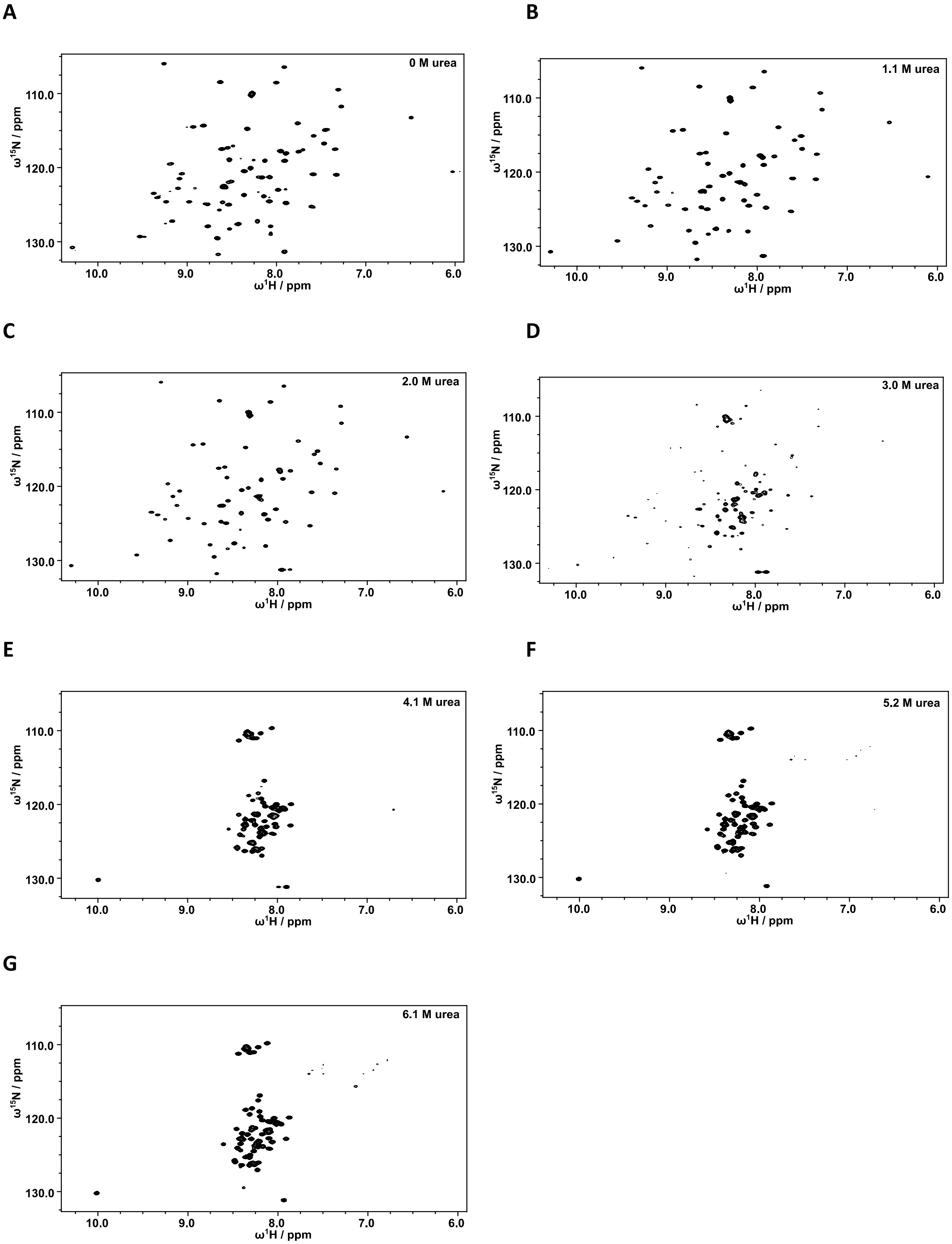

Supplementary Figure 3

Two-dimensional ^{1}H-^{15}N TROSY HSQC spectra of *Bs*CspB acquired under dilute conditions in presence of varying amounts of urea: c^{urea} = 0 M (*A*), c^{urea} = 1.1 M urea (*B*), c^{urea} = 2.0 M urea (*C*), c^{urea} = 3.0 M urea (*D*), c^{urea} = 4.1 M urea (*E*), c^{urea} = 5.2 M urea (*F*) and c^{urea} = 6.1 M urea (*G*). The decrease in peak height of NH cross-peaks comprising the native state of *Bs*CspB upon increasing concentration of urea reports on the decrease of the fraction of native protein and has subsequently been used to determine the overall thermodynamic stability of *Bs*CspB.

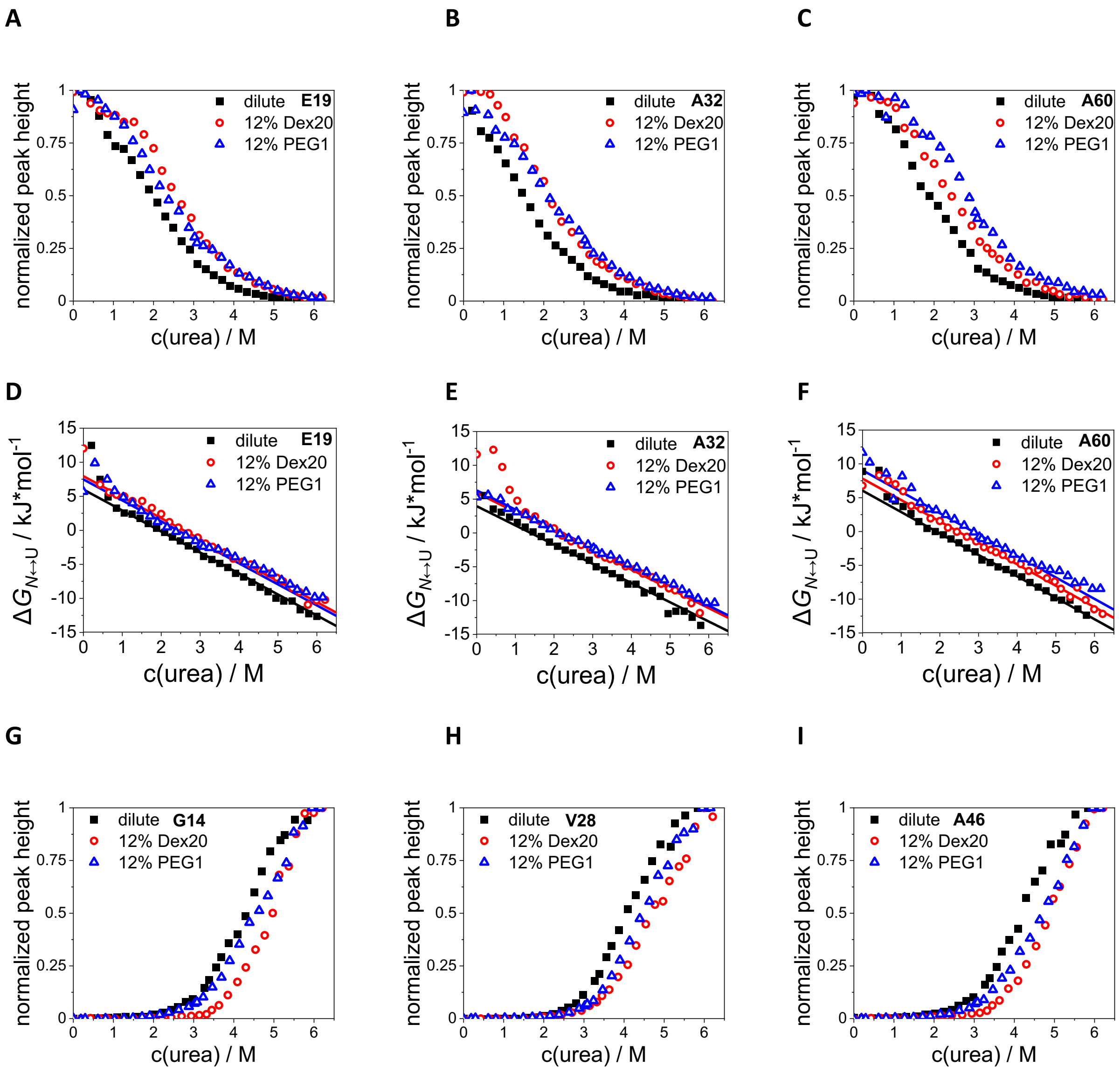

Supplementary Figure 4

Monitoring folding-to-unfolding transitions of *Bs*CspB at a residue-by-residue basis observed at T = 298 K. Chemically induced unfolding transitions of *Bs*CspB backbone amide protons of residues E19 (*A*), A32 (*B*), and A60 (*C*) applying dilute conditions (colored in black, filled rectangles), c = 120 g/L Dex20 (colored in red, open circles) or c = 120 g/L PEG1 (colored in blue, open triangles). Panels shown in (*D-F*) represent data analysis using linear extrapolation as described by Pace [1]. Direct observation of the formation of the unfolded state ensemble monitored by an increase in height of cross-peaks for G14 (*G*), V28 (*H*) and A46 (*I*). Color coding and markers have been used in the same manner as in (*A*) to (*F*).

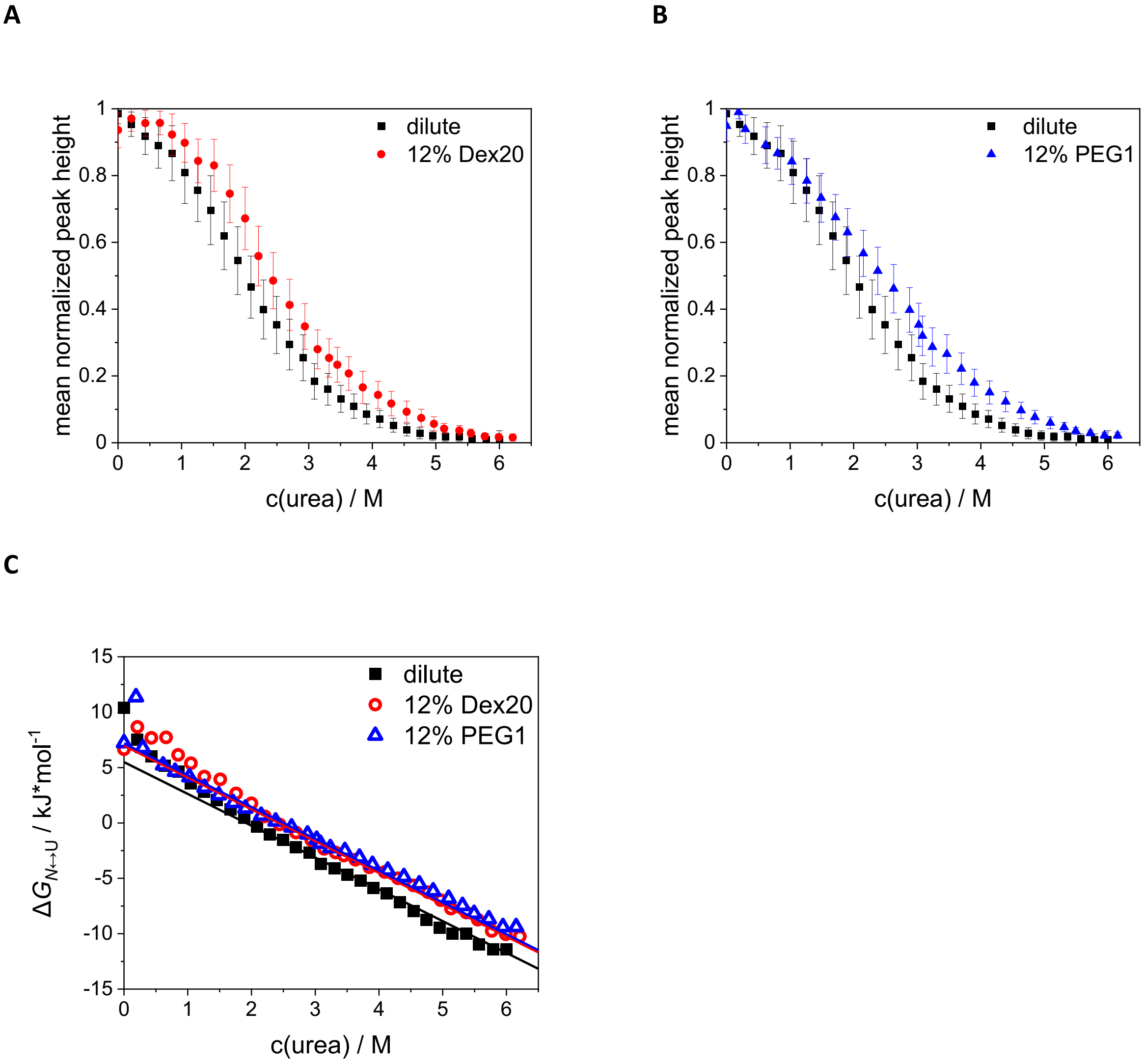

Supplementary Figure 5

Folding-to-unfolding transitions of *Bs*CspB induced by urea obtained by applying two-dimensional heteronuclear ^1H-^{15}N HSQC NMR spectroscopy. Panels in (*A*) and (*B*) represent the mean of 48 normalized individual folding-to-unfolding transitions comprising *Bs*CspB in absence (colored in black, filled rectangles) and in presence of 120 g/L Dex20 (colored in red, filled circles, *A*) or 120 g/L PEG1 (colored in blue, filled triangles, *B*). The mean normalized peak height has been computed for 48 folding-to-unfolding transitions which are distributed over the entire primary sequence of *Bs*CspB. The error bars represent one standard deviation of the mean taking all 48 normalized individual folding-to-unfolding transitions into account (n = 48). (*C*) Applying linear extrapolation to the folding-to-unfolding transitions shown in (*A*) and (*B*) using a global value for the cooperativity of folding, m. Results for this data fitting are found in Supplementary Table 1.

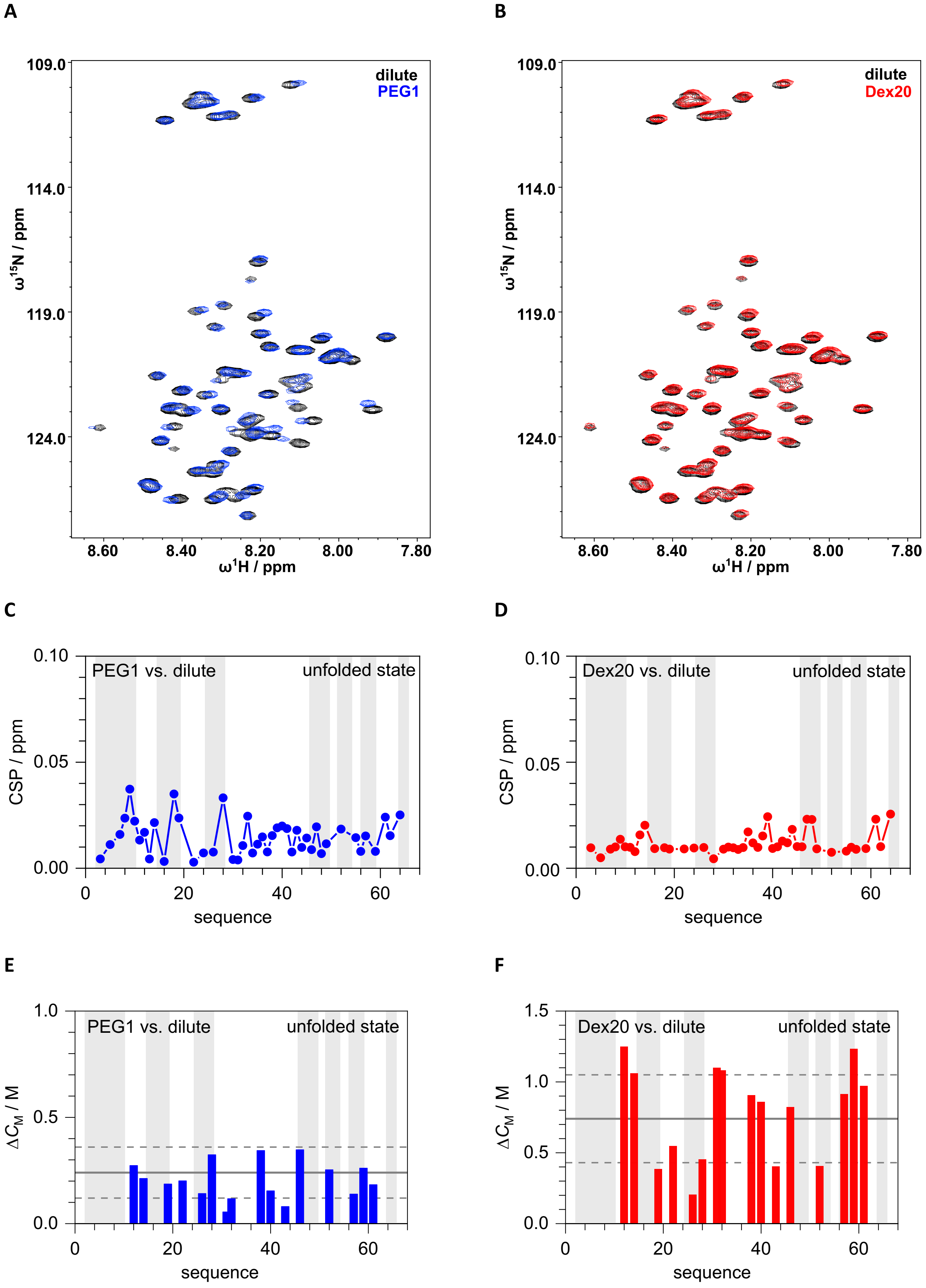
A
B
dilute
PEG1
dilute
Dex20
109.0
114.0
119.0
124.0
ω15N / ppm
8.60
8.40
8.20
8.00
7.80
ω1H / ppm
C
D
PEG1 vs. dilute
Dex20 vs. dilute
unfolded state
CSP / ppm
0.10
0.05
0.00
0
20
40
60
sequence
E
F
ΔCM / M
1.5
1.0
0.5
0.0

Supplementary Figure 6

Characterizing the potential structural impact of macromolecular crowding on the unfolded protein ensemble of *Bs*CspB. (*A*), (*B*) Overlay of two-dimensional heteronuclear ^{1}H-^{15}N HSQC spectra acquired for *Bs*CspB in the absence (colored in black) and in presence of c = 120 g/L MC: PEG1 (*A*, colored in blue) or Dex20 (*B*, colored in red). (*C*), (*D*) Chemical shift perturbations of ^{1}H-^{15}N cross-peaks comprising the unfolded protein ensemble of *Bs*CspB comparing absence with presence of MC, c = 120 g/L PEG1 (*C*, colored in blue) or Dex20 (*D*, colored in red). (*E*, *F*) Difference in the transition midpoint, ΔC_{M}, of amide protons observed in two-dimensional ^{1}H-^{15}N HSQC NMR spectra comparing dilute conditions with the presence of c = 120 g/L PEG1 (colored in blue, *E*) or c = 120 g/L Dex20 (colored in red, *F*) analyzing the unfolded protein ensemble. The mean of ΔC_{M} is shown by a horizontal line (continuous mode) whereas the mean plus or minus one standard deviation of ΔC_{M} is represented by two additional horizontal lines (dotted mode). Experimental conditions which have been used: 20 mM sodium cacodylate, pH = 7, T = 298 K, c = 6.15 M urea and c = 120 g/L PEG1; c = 6.21 M urea and c = 120 g/L Dex20; c = 6.05 M urea (dilute conditions). (*C*-*F*) Beta sheet regions according to PDB ID 1NMG are indicated by using a background colored in gray.

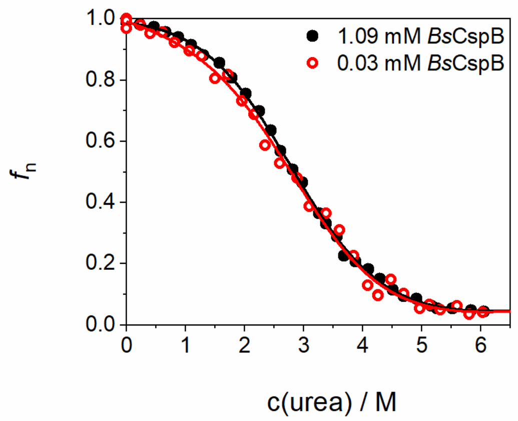

Supplementary Figure 7

Folding-to-unfolding transition of *Bs*CspB present in 20 mM sodium cacodylate at pH 7, monitored by applying one-dimensional proton NMR spectroscopy at two different protein concentrations. Closed circles (colored in black) represent a protein concentration of 1.09 mM whereas open circles (colored in red) present a protein concentration of 30 µM. Data regression according to [3] occurred with m = -3.1±0.1 kJ/mol, resulting in $\Delta G^0_{N\leftrightarrow U}$ = 8.6±0.2 kJ*mol^{-1} and 9.2±0.3 kJ*mol^{-1} for c^{BsCspB} = 1.09 mM and c^{BsCspB} = 30 µM, respectively. This leads to a transition midpoint, C_M, of C_M = 2.79±0.08 M seen for c^{BsCspB} = 1.09 mM and C_M = 2.97±0.09 M for c^{BsCspB} = 30 µM, respectively.

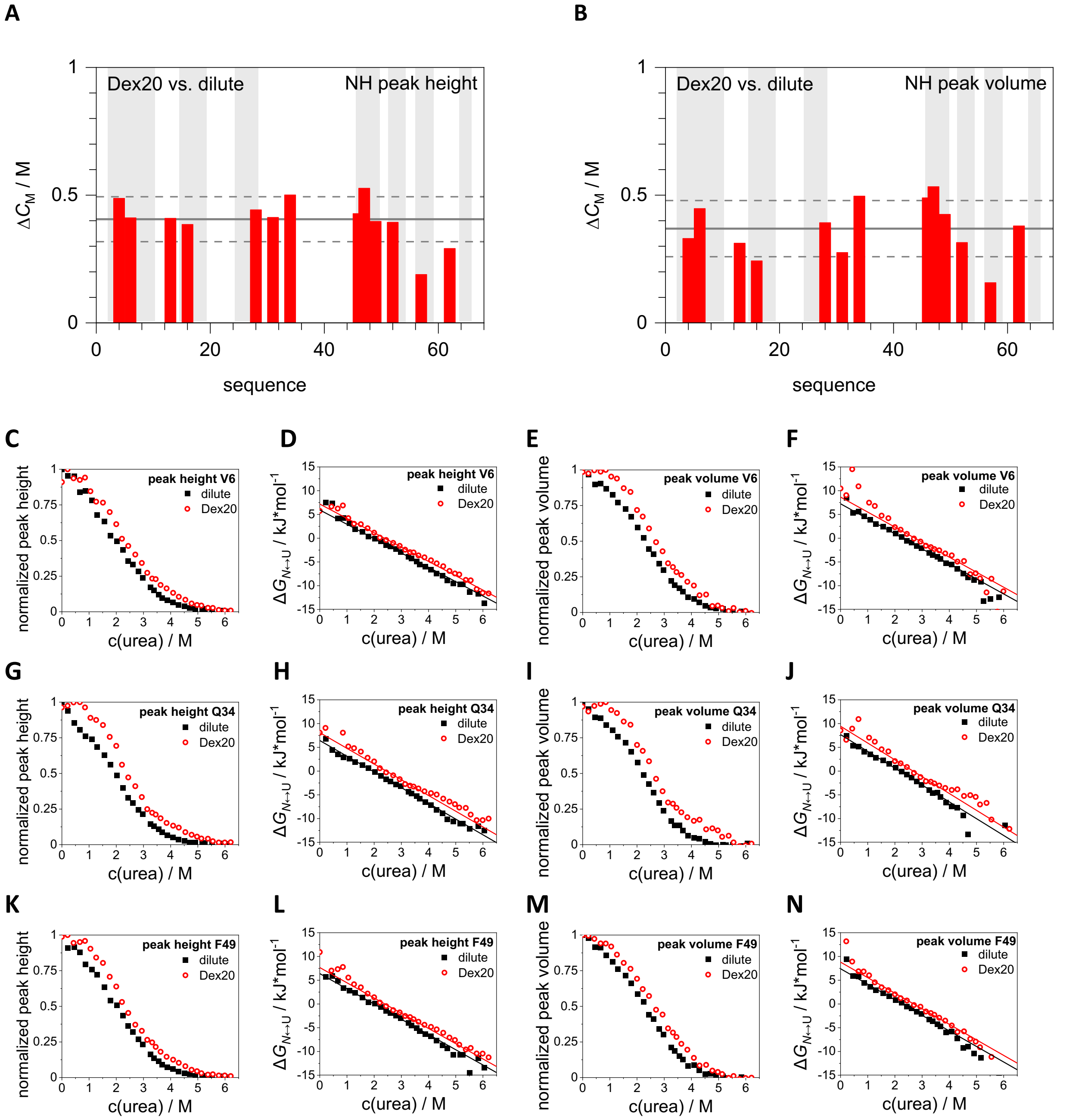

Supplementary Figure 8

Determining overall thermodynamic stability of *Bs*CspB monitored via following folding-to-unfolding transitions induced by urea using peak height or peak volume in two-dimensional NMR spectra focusing on 13 structurally well separated cross-peaks. Increase in the transition midpoint, ΔC_M, of amide protons observed in two-dimensional ^{1}H-^{15}N HSQC NMR spectra comparing dilute conditions with the presence of *c* = 120 g/L Dex20 analyzing peak heights (*A*) or peak volumes (*B*). Beta sheet regions comprising *Bs*CspB according to PDB ID 1NMG are indicated by using a background colored in gray. The mean of ΔC_M is shown

by the horizontal line (continuous mode) whereas the mean plus or minus one standard deviation of ΔC_{M} is represented by two additional horizontal lines (dotted mode). Chemically induced folding-to-unfolding transitions of *Bs*CspB backbone amide protons of residues E19 (*C*-*F*), A32 (*G*-*J*), and A60 (*K*-*N*) applying dilute conditions (colored in black, filled rectangles) or c = 120 g/L Dex20 (colored in red, open circles), using peak volume (*E*-*F*, *I*-*J*, *M*-*N*) or peak height (*C*-*D*, *G*-*H*, *K*-*L*). Panels shown in (*D*, *F*, *H*, *J*, *L*, *M*) represent data analysis using linear extrapolation as described by Pace [1].

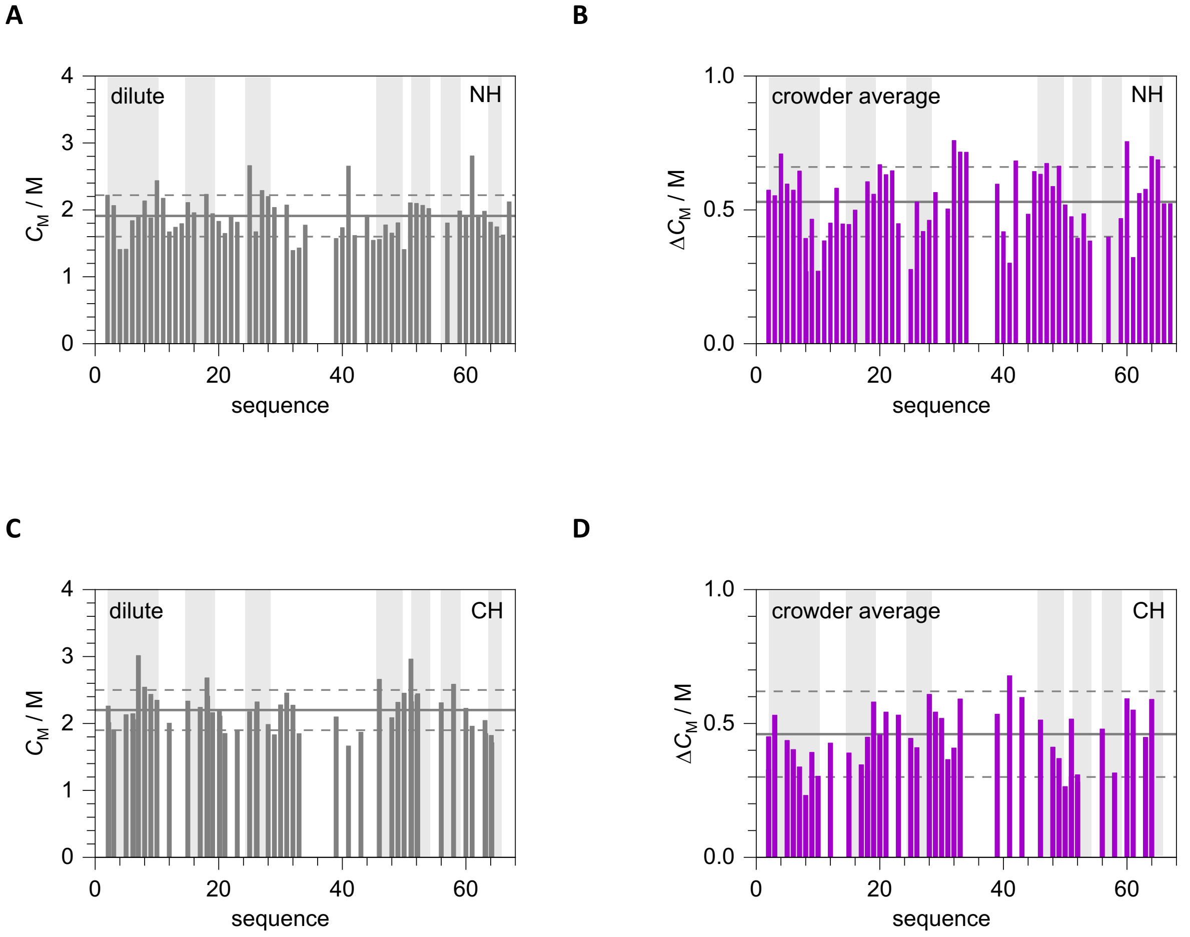

Supplementary Figure 9

Thermodynamic stability for amide protons and methine protons comprising *Bs*CspB at a residue-by-residue basis. The midpoint for folding-to-unfolding transitions, C_M, has been determined by applying two-dimensional ^{1}H-^{15}N HSQC spectroscopy (*A, B*) as well as ^{1}H-^{13}C HSQC spectroscopy (*C, D*). Error bars result from data regression by linear extrapolation. The increase in thermodynamic stability, ΔC_M, is shown in (*B*) and (*D*) based on the average obtained for the addition of c = 120 g/L PEG1 or Dex20, respectively. Beta sheet regions according to PDB ID 1NMG are indicated by using a background colored in gray. The mean of C_M (*A*, *C*) and ΔC_M (*B*, *D*) is shown by a horizontal line (continuous mode) whereas the mean plus or minus one standard deviation of C_M (*A, C*) or ΔC_M (*B, D*) is represented by two additional horizontal lines (dotted mode).

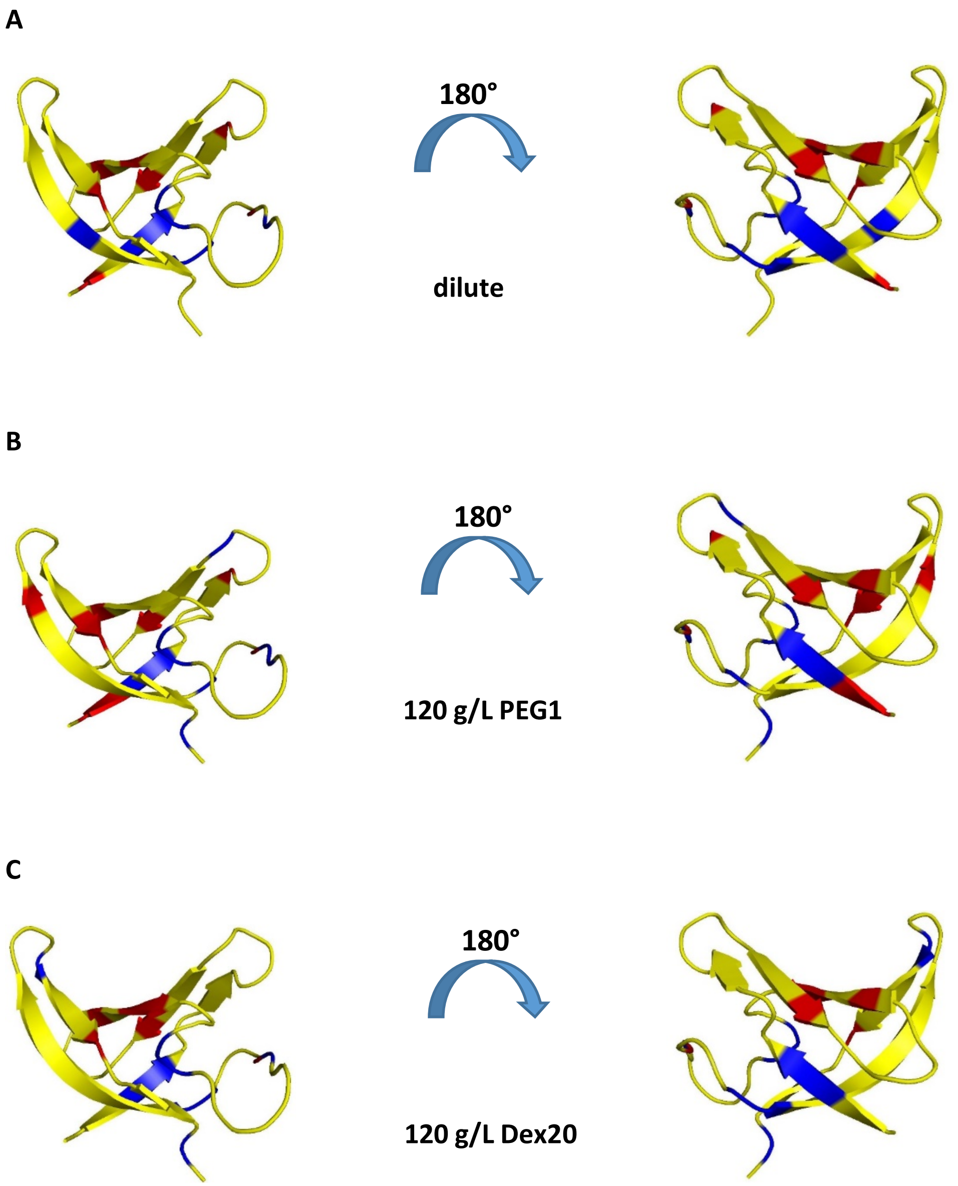

Supplementary Figure 10

Illustrating thermodynamic stability of residues comprising BsCspB which deviate from the mean. The NMR solution structure of *Bs*CspB (PDB ID: 1NMG) has been used to illustrate values of C_M obtained for NH resonance signals which are below the mean and one standard deviation of C_M (colored in blue) or above the mean plus one standard deviation of C_M (colored in red). Residues fulfilling these constraints are shown for dilute (*A*), c = 120 g/L PEG1 (*B*), and c = 120 g/L Dex20 (*C*) conditions. Corresponding numerical values of C_M used to prepare these figures are presented in Supplementary Table 2, the mean and one standard deviation are found in Supplementary Table 1.

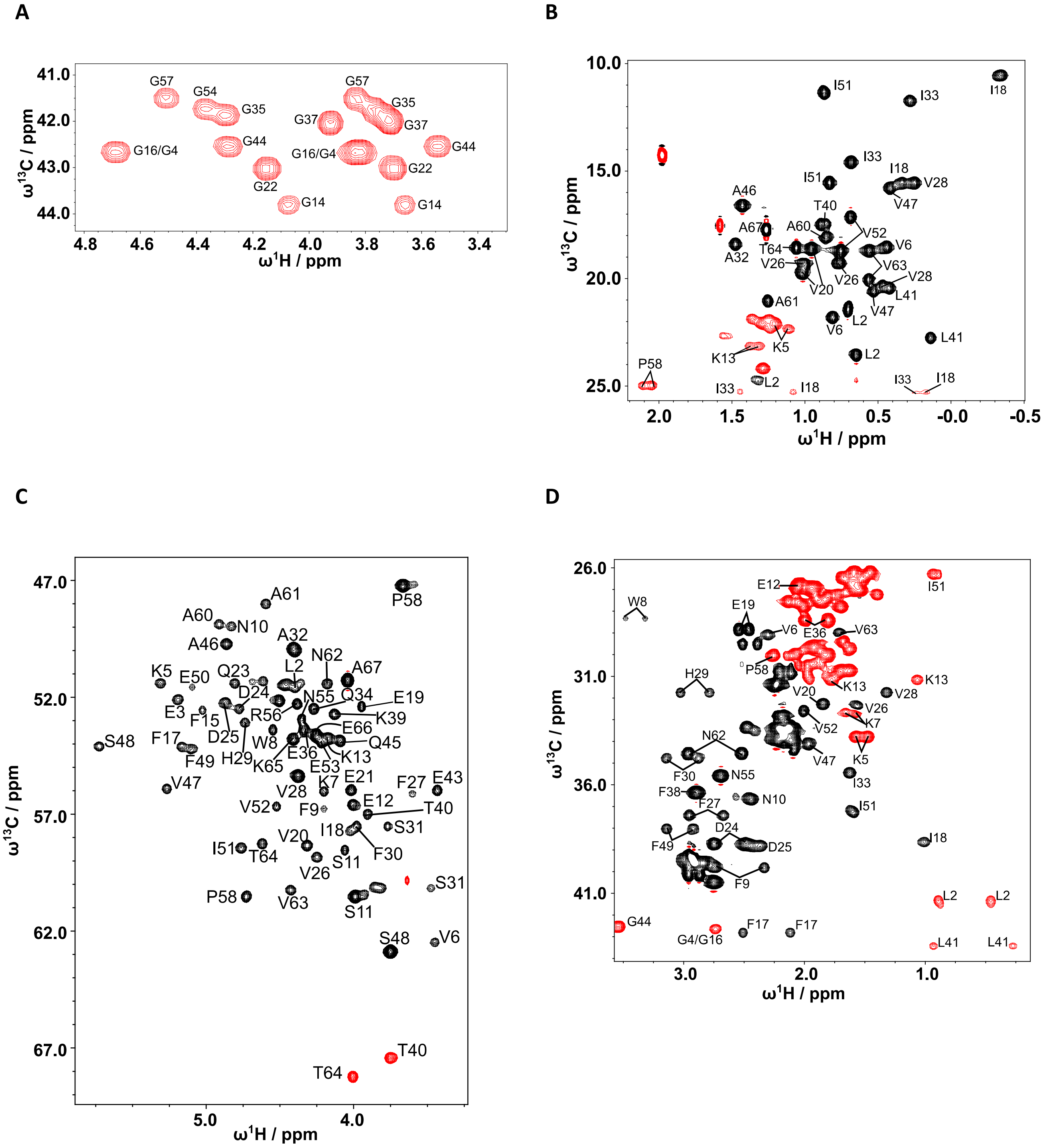

Supplementary Figure 11

Assignment of ^{1}H-^{13}C resonance signals representing the native state of *Bs*CspB using the one letter code of amino acids followed by the position in the primary sequence. Note that the two-dimensional ^{1}H-^{13}C HSQC NMR spectra have been acquired by using the constant time approach resulting in positive (colored in black) and negative peak heights (colored in red). (*A*) C_{α}-H_{α} cross-peaks corresponding to backbone CH_2

groups of glycine residues. (*B*) Cross-peaks comprising side chains showing ^{13}C resonances ranging from 10 to 25 ppm. (*C*) Cross-peaks comprising backbone CH signals showing ^{13}C resonances ranging from 47 to 69 ppm. (*D*) Cross-peaks comprising side chains showing ^{13}C resonances ranging from 26 to 44 ppm.

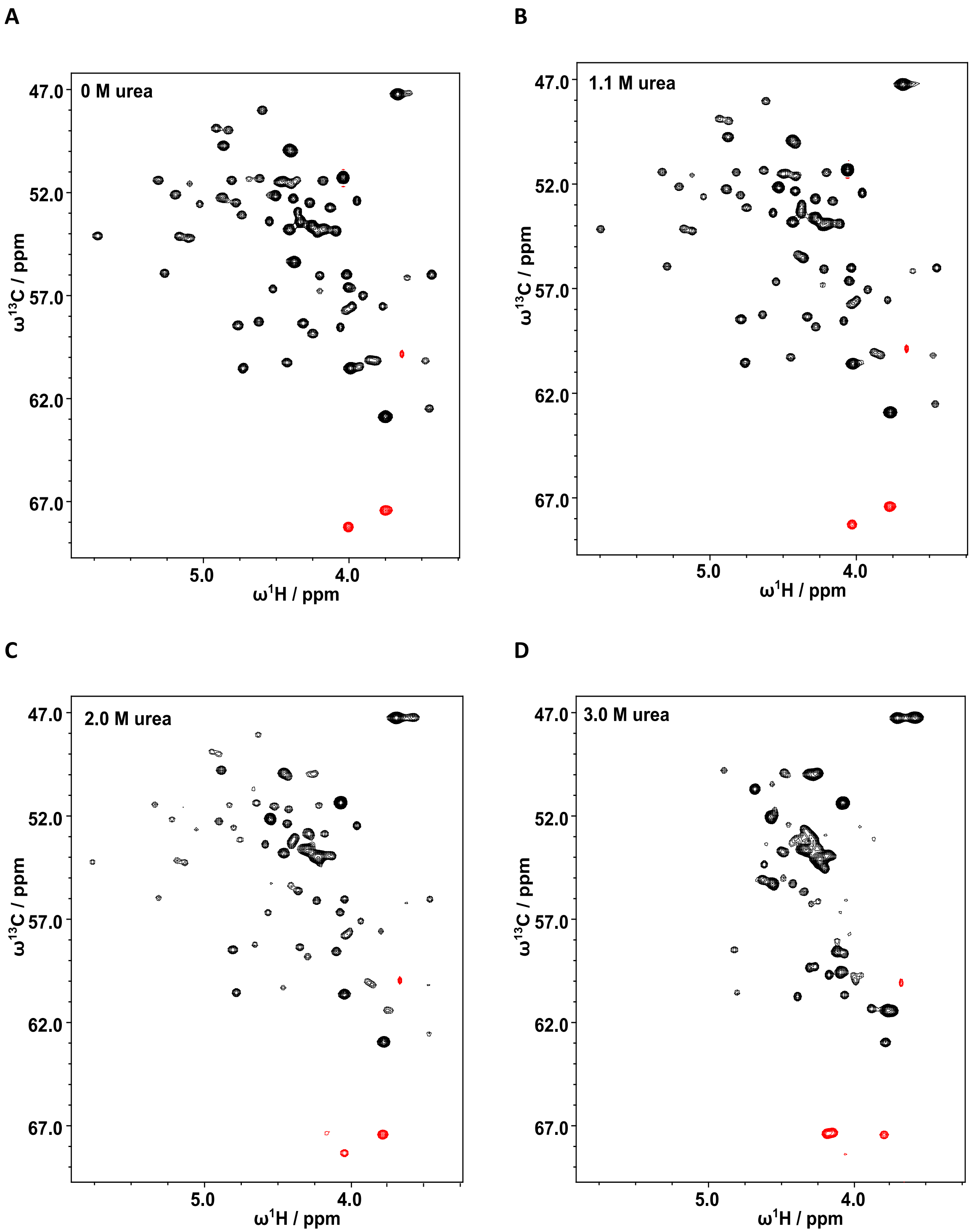

A
0 M urea
B
1.1 M urea
C
2.0 M urea
D
3.0 M urea
47.0
52.0
57.0
62.0
67.0
5.0
4.0
ω13C / ppm
ω1H / ppm

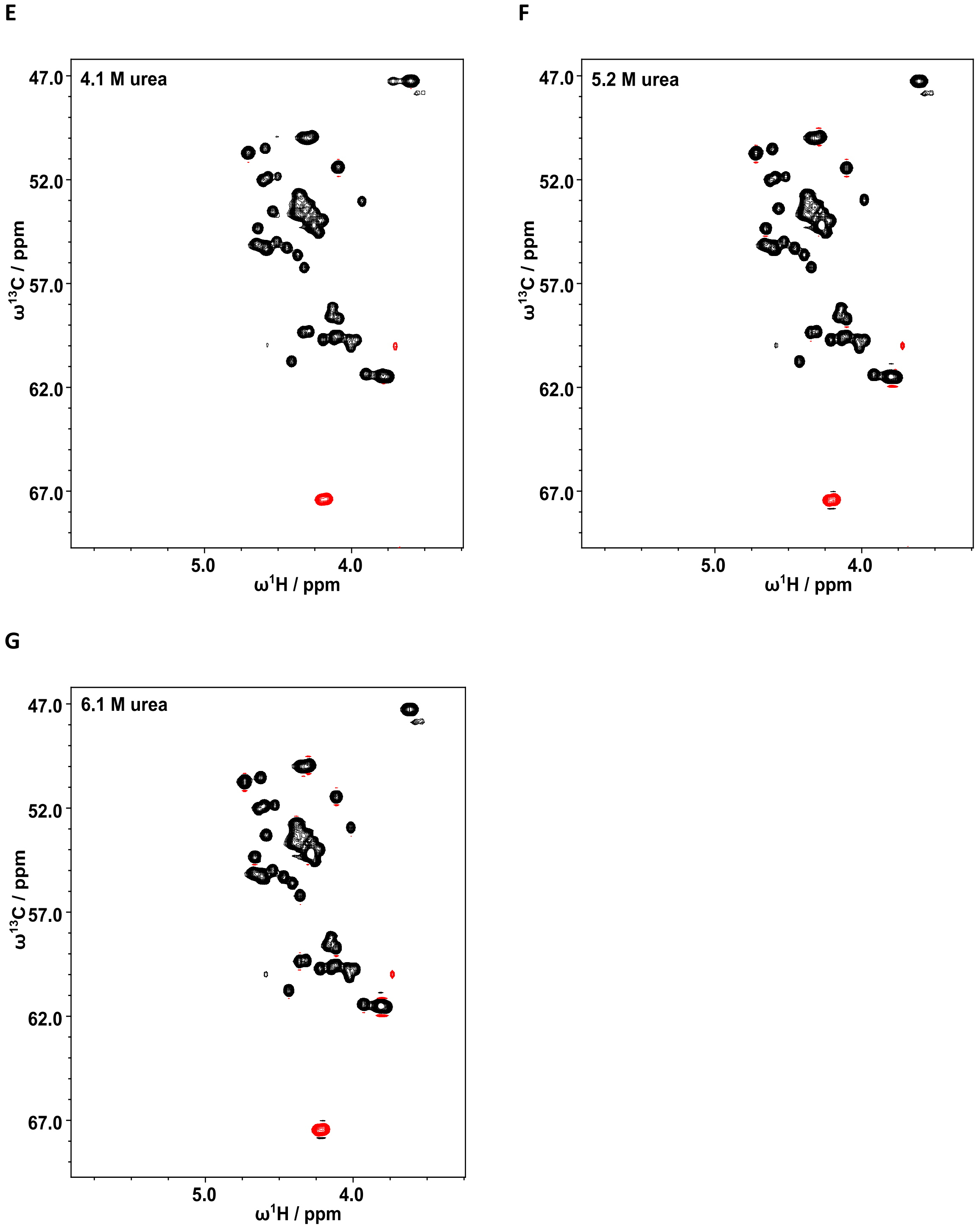

Supplementary Figure 12

Two-dimensional ^{1}H-^{13}C HSQC spectra of *Bs*CspB showing ^{13}C resonances ranging from 46 to 70 ppm acquired under dilute conditions in presence of varying amounts of urea: c^{urea} = 0 M (*A*), c^{urea} = 1.1 M urea (*B*), c^{urea} = 2.0 M urea (*C*), c^{urea} = 3.0 M urea (*D*), c^{urea} = 4.1 M urea (*E*), c^{urea} = 5.2 M urea (*F*) and c^{urea} = 6.1 M urea (*G*). The decrease in peak height of cross-peaks comprising the native state of *Bs*CspB upon increasing

concentration of urea reports on the decrease of the fraction of native protein and has subsequently been used to determine the overall thermodynamic stability of *Bs*CspB. The two-dimensional ^{1}H-^{13}C HSQC NMR spectra have been acquired by using the constant time approach resulting in positive (colored in black) and negative peak heights (colored in red).

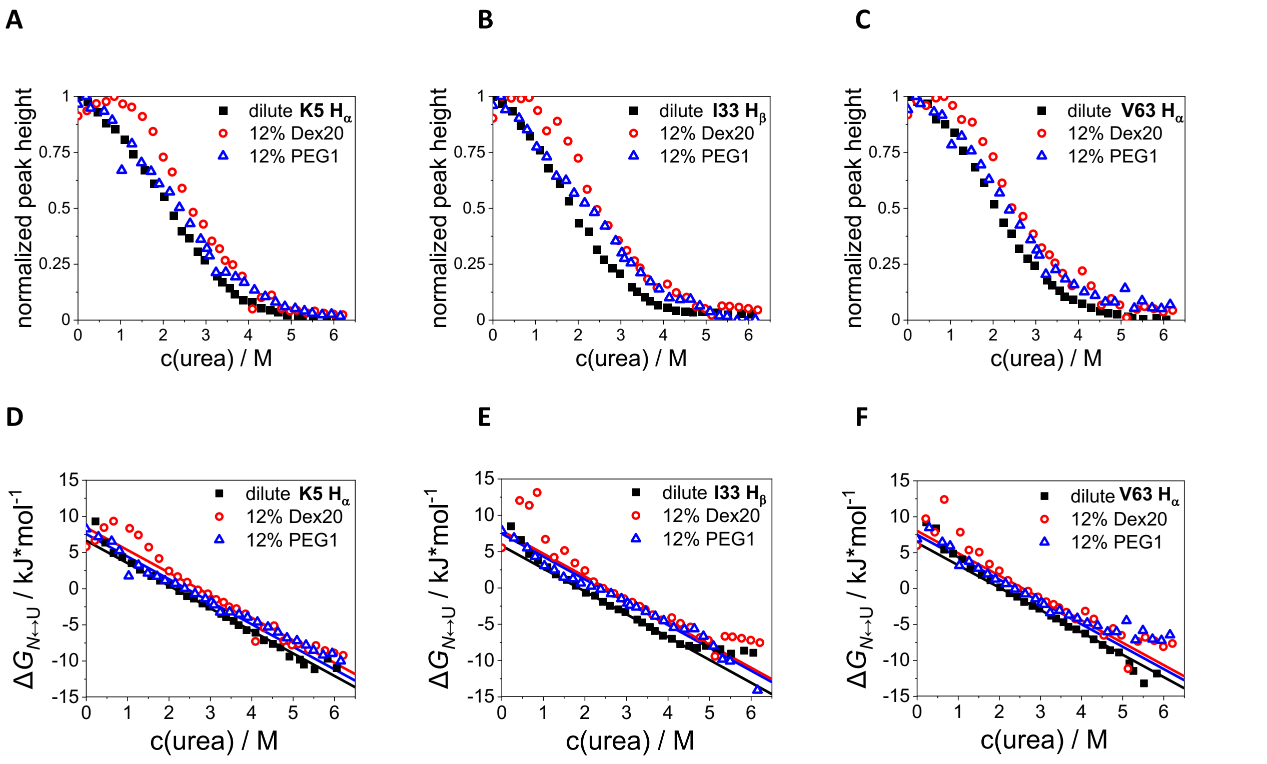

Supplementary Figure 13

Monitoring folding-to-unfolding transitions of *Bs*CspB at a residue-by-residue basis. (*A*-*C*) Chemically induced folding-to-unfolding transitions of protons comprising a methine group (CH) of residues K5 (*A*), I33 (*B*), V63 (*C*) derived from two-dimensional heteronuclear ^{1}H-^{15}N HSQC spectroscopy of samples containing no MC agents (colored in black, filled rectangles), *c* = 120 g/L Dex20 (colored in red, open circles) or *c* = 120 g/L PEG1 (colored in blue, open triangles). Panels shown in (*D*-*F*) represent data analysis using linear extrapolation. Color coding and markers have been used in the same manner as in (*A*-*C*). Numerical results for this data fitting are provided in Supplementary Table 3.

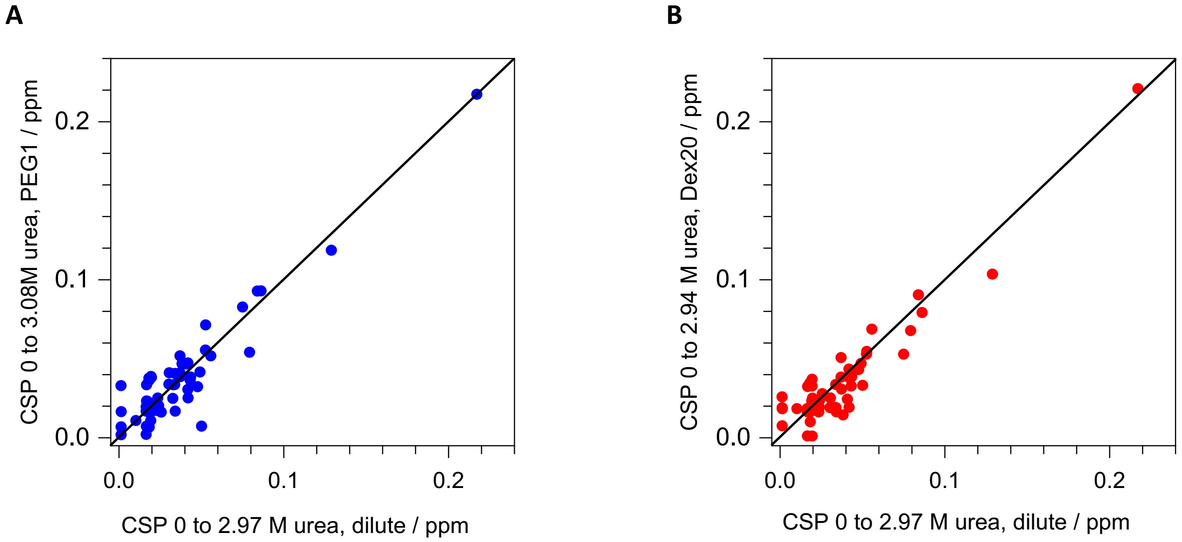

Supplementary Figure 14

Comparing chemical shift perturbations of NH cross-peaks comprising *Bs*CspB observed in two-dimensional ^{1}H-^{15}N HSQC NMR spectra induced by addition of $c^{urea} = 2.97$ M under dilute conditions (x-axis) with chemical shift perturbations of *Bs*CspB induced by addition of $c^{urea} = 3.08$ M in presence of $c = 120$ g/L PEG1 (y-axis) (*A*) or induced by addition of $c^{urea} = 2.94$ M in presence of $c = 120$ g/L Dex20 (y-axis) (*B*). Values for chemical shift perturbations induced by adding urea obtained under dilute, $c^{PEG1} = 120$ g/L PEG1 and $c^{Dex20} = 120$ g/L conditions are individually shown in Figure 4B.

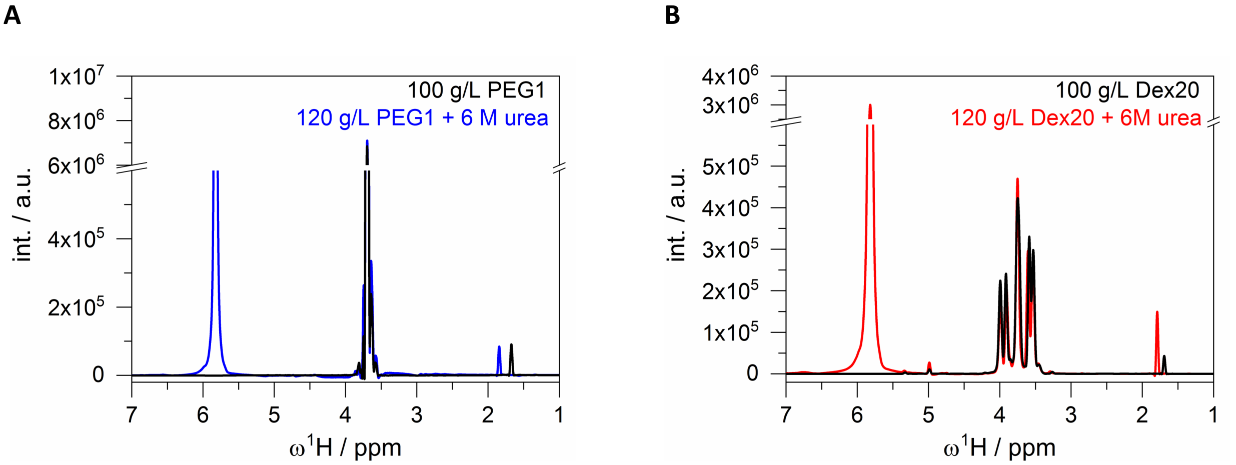

Supplementary Figure 15

One-dimensional proton NMR spectra of MC agents in absence and presence of urea. (*A*) Proton NMR spectrum of PEG1 in the absence (colored in black) and in presence of c^{urea} = 6 M urea (colored in blue). (B) One-dimensional proton NMR spectrum of Dex20 in the absence (colored in black) and in presence of c^{urea} = 6 M urea (colored in red). All samples contain sodium cacodylate as buffering agent (resonance signal at about ω^{1H} = 1.8 ppm) and the spectra are referenced using the proton resonance signal of TMSP.

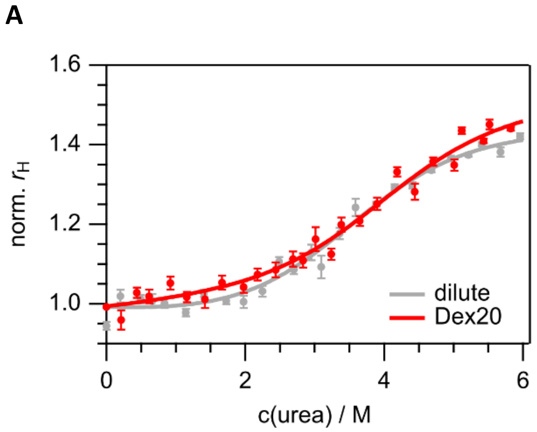

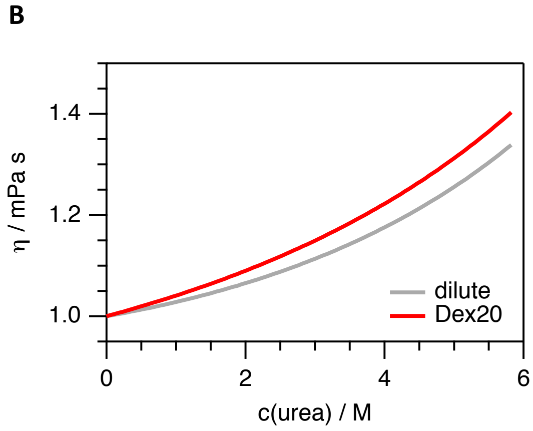

Supplementary Figure 16

Diffusion properties of *Bs*CspB under dilute and macromolecular crowding conditions. (*A*) Folding-to-unfolding transition of *Bs*CspB applying dilute conditions (colored in gray) and c = 120 g/L Dex20 (colored in red) following the change in hydrodynamic dimension. The diffusion coefficient of *Bs*CspB was determined by integrating the range of chemical shifts between 6.5 and 9.5 ppm for signals representing the native state and between 6.8 and 8.6 ppm for signals representing the unfolded protein ensemble monitoring one-dimensional proton NMR spectra. The fitting of the Stejskal and Tanner equation $I(G) = I(0)\exp(-G^2\gamma^2\delta^2 D(\Delta-\delta/3))$ to the experimentally acquired data by applying 21 different gradient strengths (n = 30 in total as three gradient strengths have been repeated) reports on an error for the diffusion coefficient D and, consequently, by using Einsteins relation $D = k_B T/(6r_H\pi\eta)$ on an error for the hydrodynamic radius, r_H, which is shown as error bar for every concentration of urea which has been applied. Results for data fitting applying a two-state folding model are found in Supplementary Table 1. (*B*) Dependence of the microviscosity of the buffer on the concentration of urea using TMSP as internal reference under dilute (colored in gray) and MC conditions, c = 120 g/L Dex20 (colored in red).

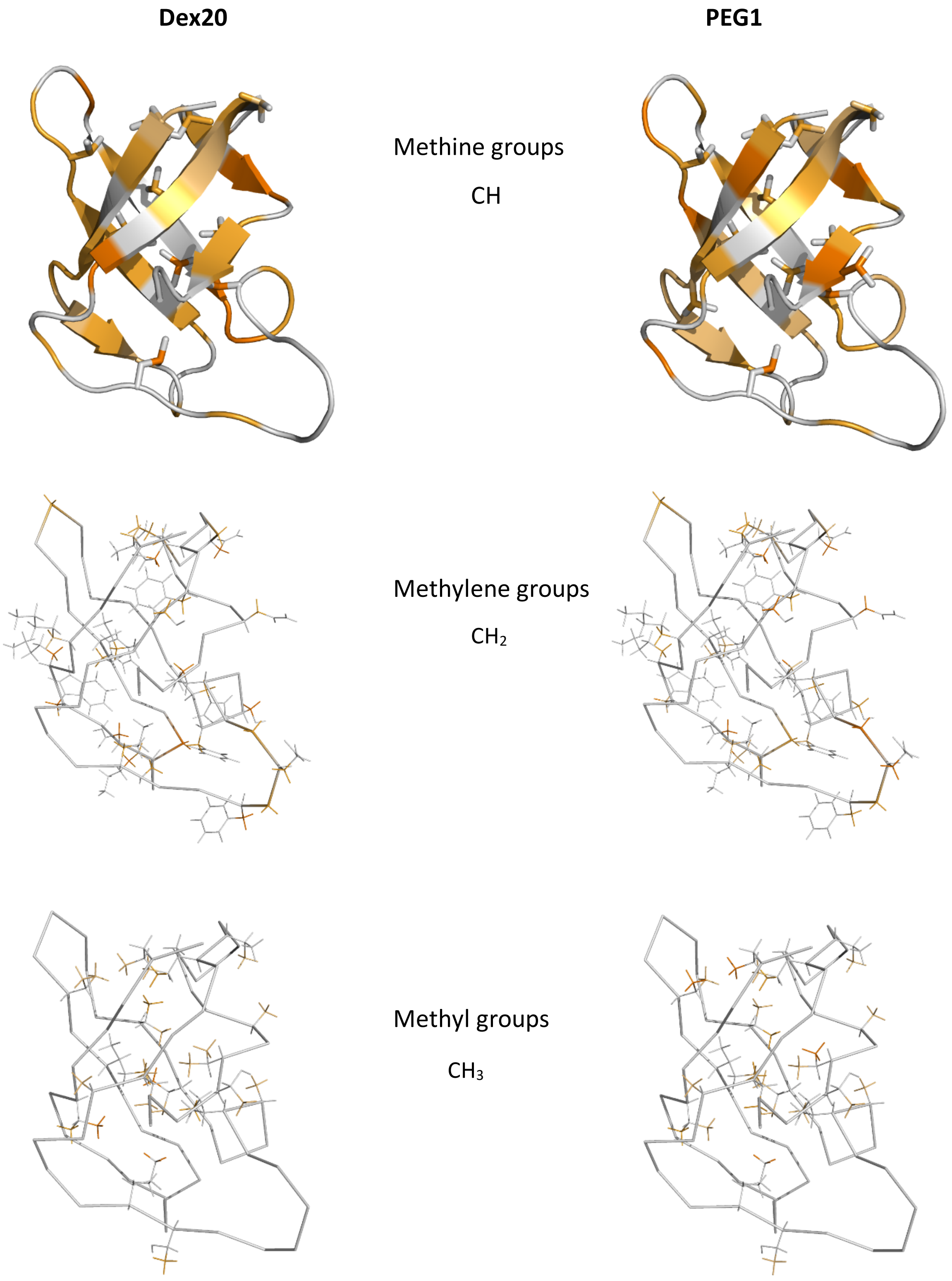
Dex20
PEG1
Methine groups
CH
Methylene groups
CH2
Methyl groups
CH3

Supplementary Figure 17

Illustrating the increase in thermodynamic stability due to the presence of MC agents Dex20 (left) or PEG1 (right) on the three-dimensional structure of *Bs*CspB. The increase in the transition midpoint, ΔC_M, obtained for carbon bound protons is highlighted by using the following color coding:

Methine groups, Dex20: 0.3 M ≤ ΔC_M ≤ 0.4 M in light orange, 0.5 M ≤ ΔC_M ≤ 0.6 M in orange and 0.7 M ≤ ΔC_M ≤ 0.8 M in bright orange; PEG1: 0.0 M ≤ ΔC_M ≤ 0.2 M in light orange, 0.3 M ≤ ΔC_M ≤ 0.4 M in orange and 0.5 M ≤ ΔC_M ≤ 0.7 M in bright orange. Note that carbon atoms present in side chain CH groups have been highlighted, no protons shown. Numerical values for ΔC_M obtained under these experimental conditions can be found in Figure 3*A*, *B* and Supplementary Table 3.

Methylene groups, Dex20: 0.3 M ≤ ΔC_M ≤ 0.4 M in light orange, 0.5 M ≤ ΔC_M ≤ 0.6 M in orange and 0.7 M ≤ ΔC_M ≤ 1.5 M in bright orange; PEG1: -0.1 M ≤ ΔC_M ≤ 0.1 M in light orange, 0.2 M ≤ ΔC_M ≤ 0.3 M in orange and 0.4 M ≤ ΔC_M ≤ 0.7 M in bright orange. Note that both carbon atoms and protons comprising side chain CH_2 groups have been highlighted. Numerical values for ΔC_M obtained under these experimental conditions can be found in Figure 3*C*, *D* and Supplementary Table 4.

Methyl groups, Dex20: ΔC_M = 0.6 M in light orange, ΔC_M = 0.7 M in orange and 0.8 M ≤ ΔC_M ≤ 1 M in bright orange; PEG1: -0.2 M ≤ ΔC_M ≤ 0.4 M in light orange, 0.5 M ≤ ΔC_M ≤ 0.6 M in orange and 0.7 M ≤ ΔC_M ≤ 1.1 M in bright orange. Note that both carbon atoms and protons comprising side chain CH_3 groups have been highlighted. Numerical values for ΔC_M obtained under these experimental conditions can be found in Fig. 3*E*, *F* and Supplementary Table 5.

Supplementary Table 1

Analysis of one-dimensional ^{1}H NMR spectroscopically detected folding-to-unfolding transitions shown in Figure 2*A*, *B* and mean values for two-dimensional heteronuclear ^{1}H-^{15}N HSQC detected folding-to-unfolding transitions shown in Supplementary Figure 4. Data analysis occurred for the transition derived from the integration of chemical shifts representing folded and unfolded signal regions in one-dimensional ^{1}H NMR spectra using the approach introduced by Santoro and Bolen [2,3] highlighted by $^{+}$. Folding-to-unfolding transitions of the mean normalized peak heights of cross-peaks acquired in ^{1}H-^{15}N and ^{1}H-^{13}C HSQC spectra representing the native state of *Bs*CspB (^{nat}) or in ^{1}H-^{15}N HSQC spectra representing the unfolded protein ensemble (^{unf}) have been fitted using the transition region ranging from *c* = 2 to 4 M urea applying linear extrapolation introduced by Greene et *al.* [4] due to the heterogeneous slopes present in native and unfolded baselines and using a global value for the folding cooperativity, *m*.

Method	condition	$\Delta G^{0}_{N\leftrightarrow U}$ / kJ*mol^{-1}	m / kJ*(mol*M)$^{-1}$	C_M / M	ΔC_M / M
^{1}H spectra^{+}	dil	8.8 ± 0.9	-3.2 ± 0.2	2.7 ± 0.3	
^{1}H spectra^{+}	Dex20	10.3 ± 0.5	-3.2 ± 0.2	3.2 ± 0.3	0.5
^{1}H spectra^{+}	PEG1	10.7 ± 0.7	-3.2 ± 0.2	3.3 ± 0.3	0.6
^{1}H spectra	dil	8.4 ± 0.2	-3.0 ± 0.1	2.8 ± 0.1	
^{1}H spectra	Dex20	9.7 ± 0.2	-3.0 ± 0.1	3.3 ± 0.1	0.4
^{1}H spectra	PEG1	9.8 ± 0.2	-3.0 ± 0.1	3.3 ± 0.1	0.4
mean HN^{nat}	dil	5.5 ± 0.2	-2.9 ± 0.1	1.9 ± 0.1	
mean HN^{nat}	Dex20	7.0 ± 0.2	-2.9 ± 0.1	2.4 ± 0.1	0.5
mean HN^{nat}	PEG1	7.2 ± 0.2	-2.9 ± 0.1	2.5 ± 0.1	0.6
mean HN^{unf}	dil	18.5 ± 1.4	-4.3 ± 0.4	4.3 ± 0.2	
mean HN^{unf}	Dex20	21.9 ± 2.3	-4.3 ± 0.4	5.1 ± 0.4	0.7
mean HN^{unf}	PEG1	19.4 ± 1.4	-4.3 ± 0.4	4.5 ± 0.2	0.2
mean CH	dil	6.3 ± 0.4	-3.0 ± 0.1	2.1 ± 0.1	
mean CH	Dex20	8.0 ± 0.4	-3.0 ± 0.1	2.6 ± 0.2	0.6
mean CH	PEG1	7.2 ± 0.4	-3.0 ± 0.1	2.4 ± 0.2	0.3
mean CH_2	dil	6.2 ± 0.3	-3.0 ± 0.1	2.1 ± 0.1	
mean CH_2	Dex20	8.2 ± 0.3	-3.0 ± 0.1	2.7 ± 0.1	0.6
mean CH_2	PEG1	7.5 ± 0.3	-3.0 ± 0.1	2.5 ± 0.1	0.4
mean CH_3	dil	5.9 ± 0.2	-2.9 ± 0.1	2.0 ± 0.1	
mean CH_3	Dex20	7.7 ± 0.2	-2.9 ± 0.1	2.6 ± 0.1	0.6
mean CH_3	PEG1	7.1 ± 0.2	-2.9 ± 0.1	2.4 ± 0.1	0.4
diffusion	dil	9.4 ± 1.7	-3.0 ± 0.1		
diffusion	Dex20	11.1 ± 3.2	-3.0 ± 0.1		0.6

Supplementary Table 2

Results obtained for amide protons (NH) using linear extrapolation of the transition region ranging from c = 2 to 4 M urea in two-dimensional heteronuclear ^{1}H-^{15}N HSQC spectra. Folding-to-unfolding transitions have been acquired under three different conditions abbreviated using dil (dilute conditions), dex (c = 120 g/L Dex20) and peg (c = 120 g/L PEG1). Each single residue was fitted using a global value for the cooperativity of folding, m, applying all three different experimental conditions as discussed previously [5]. ^{sc}: side chain. Values for ΔC_M are shown in Figure 2*C*, *D* and are graphically highlighted in Figure 5.

residue		$\Delta G^0_{N\leftrightarrow U}$ / kJ*mol$^{-1.}$	m / kJ*(mol*M)$^{-1}$	C_M / M	ΔC_M / M
2	dil	7.2 ± 0.3	-3.2 ± 0.1	2.2 ± 0.1	
2	dex	8.7 ± 0.3	-3.2 ± 0.1	2.7 ± 0.1	0.5
2	peg	9.4 ± 0.3	-3.2 ± 0.1	2.9 ± 0.1	0.7
3	dil	7.3 ± 0.9	-3.5 ± 0.3	2.1 ± 0.3	
3	dex	8.2 ± 0.9	-3.5 ± 0.3	2.3 ± 0.3	0.3
3	peg	10.2 ± 0.9	-3.5 ± 0.3	2.9 ± 0.4	0.8
4	dil	4.0 ± 0.2	-2.8 ± 0.1	1.4 ± 0.1	
4	dex	5.8 ± 0.2	-2.8 ± 0.1	2.0 ± 0.1	0.6
4	peg	6.2 ± 0.2	-2.8 ± 0.1	2.2 ± 0.1	0.8
5	dil	3.8 ± 0.2	-2.7 ± 0.1	1.4 ± 0.1	
5	dex	5.1 ± 0.2	-2.7 ± 0.1	1.9 ± 0.1	0.5
5	peg	5.8 ± 0.2	-2.7 ± 0.1	2.1 ± 0.1	0.7
6	dil	5.3 ± 0.2	-2.9 ± 0.1	1.8 ± 0.1	
6	dex	6.8 ± 0.2	-2.9 ± 0.1	2.4 ± 0.1	0.5
6	peg	7.1 ± 0.2	-2.9 ± 0.1	2.5 ± 0.1	0.6
7	dil	5.8 ± 0.2	-3.0 ± 0.1	1.9 ± 0.1	
7	dex	7.6 ± 0.2	-3.0 ± 0.1	2.5 ± 0.1	0.6
7	peg	7.8 ± 0.2	-3.0 ± 0.1	2.6 ± 0.1	0.7
8	dil	6.4 ± 0.3	-3.0 ± 0.1	2.1 ± 0.1	
8	dex	7.5 ± 0.3	-3.0 ± 0.1	2.5 ± 0.1	0.4
8	peg	7.7 ± 0.3	-3.0 ± 0.1	2.6 ± 0.1	0.4
9	dil	5.8 ± 0.2	-3.1 ± 0.1	1.9 ± 0.1	
9	dex	7.2 ± 0.2	-3.1 ± 0.1	2.3 ± 0.1	0.4
9	peg	7.3 ± 0.2	-3.1 ± 0.1	2.4 ± 0.1	0.5
10	dil	8.0 ± 0.4	-3.3 ± 0.1	2.4 ± 0.1	
10	dex	8.6 ± 0.4	-3.3 ± 0.1	2.6 ± 0.1	0.2
10	peg	9.2 ± 0.4	-3.3 ± 0.1	2.8 ± 0.2	0.4
11	dil	6.3 ± 0.2	-2.9 ± 0.1	2.2 ± 0.1	
11	dex	7.8 ± 0.2	-2.9 ± 0.1	2.7 ± 0.1	0.5
11	peg	7.1 ± 0.2	-2.9 ± 0.1	2.4 ± 0.1	0.3
13	dil	5.2 ± 0.2	-3.0 ± 0.1	1.7 ± 0.1	
13	dex	6.8 ± 0.2	-3.0 ± 0.1	2.3 ± 0.1	0.5
13	peg	7.1 ± 0.2	-3.0 ± 0.1	2.4 ± 0.1	0.6

14	dil	5.3	±	0.3	-2.9 ±	0.1	1.8	±	0.1	
14	dex	6.7	±	0.3	-2.9 ±	0.1	2.3	±	0.1	0.5
14	peg	6.5	±	0.3	-2.9 ±	0.1	2.2	±	0.1	0.4
15	dil	6.0	±	0.2	-2.8 ±	0.1	2.1	±	0.1	
15	dex	6.9	±	0.2	-2.8 ±	0.1	2.4	±	0.1	0.3
15	peg	7.5	±	0.2	-2.8 ±	0.1	2.7	±	0.1	0.6
16	dil	6.1	±	0.2	-3.1 ±	0.1	2.0	±	0.1	
16	dex	7.7	±	0.2	-3.1 ±	0.1	2.5	±	0.1	0.5
16	peg	7.7	±	0.2	-3.1 ±	0.1	2.4	±	0.1	0.5
18	dil	7.2	±	0.5	-3.2 ±	0.2	2.2	±	0.2	
18	dex	9.1	±	0.5	-3.2 ±	0.2	2.8	±	0.2	0.6
18	peg	9.3	±	0.5	-3.2 ±	0.2	2.9	±	0.2	0.6
19	dil	6.0	±	0.3	-3.1 ±	0.1	1.9	±	0.1	
19	dex	7.9	±	0.3	-3.1 ±	0.1	2.6	±	0.1	0.6
19	peg	7.5	±	0.3	-3.1 ±	0.1	2.4	±	0.1	0.5
20	dil	5.0	±	0.2	-2.7 ±	0.1	1.8	±	0.1	
20	dex	7.0	±	0.2	-2.7 ±	0.1	2.5	±	0.1	0.7
20	peg	6.7	±	0.2	-2.7 ±	0.1	2.5	±	0.1	0.6
21	dil	5.0	±	0.3	-3.0 ±	0.1	1.6	±	0.1	
21	dex	6.7	±	0.3	-3.0 ±	0.1	2.2	±	0.1	0.6
21	peg	7.1	±	0.3	-3.0 ±	0.1	2.3	±	0.1	0.7
22	dil	5.2	±	0.5	-2.7 ±	0.2	1.9	±	0.2	
22	dex	6.6	±	0.6	-2.7 ±	0.2	2.4	±	0.3	0.5
22	peg	7.2	±	0.6	-2.7 ±	0.2	2.7	±	0.3	0.8
23	dil	5.5	±	0.2	-3.0 ±	0.1	1.8	±	0.1	
23	dex	6.7	±	0.2	-3.0 ±	0.1	2.2	±	0.1	0.4
23	peg	7.0	±	0.3	-3.0 ±	0.1	2.3	±	0.1	0.5
25	dil	8.0	±	0.4	-3.0 ±	0.1	2.7	±	0.2	
25	dex	9.2	±	0.5	-3.0 ±	0.1	3.1	±	0.2	0.4
25	peg	8.4	±	0.5	-3.0 ±	0.1	2.8	±	0.2	0.2
27	dil	7.5	±	0.4	-3.3 ±	0.1	2.3	±	0.2	
27	dex	9.5	±	0.4	-3.3 ±	0.1	2.9	±	0.2	0.6
27	peg	8.2	±	0.4	-3.3 ±	0.1	2.5	±	0.2	0.2
28	dil	6.5	±	0.3	-3.0 ±	0.1	2.2	±	0.1	
28	dex	8.3	±	0.3	-3.0 ±	0.1	2.8	±	0.1	0.6
28	peg	7.5	±	0.3	-3.0 ±	0.1	2.5	±	0.1	0.3
29	dil	5.8	±	0.3	-2.9 ±	0.1	2.0	±	0.1	
29	dex	7.7	±	0.3	-2.9 ±	0.1	2.7	±	0.1	0.7
29	peg	7.2	±	0.3	-2.9 ±	0.1	2.5	±	0.1	0.5
31	dil	6.6	±	0.3	-3.2 ±	0.1	2.1	±	0.1	
31	dex	8.2	±	0.3	-3.2 ±	0.1	2.6	±	0.1	0.5
31	peg	8.0	±	0.3	-3.2 ±	0.1	2.5	±	0.1	0.5
32	dil	4.0	±	0.2	-2.8 ±	0.1	1.4	±	0.1	
32	dex	5.9	±	0.2	-2.8 ±	0.1	2.1	±	0.1	0.7
32	peg	6.3	±	0.2	-2.8 ±	0.1	2.2	±	0.1	0.8
33	dil	3.7	±	0.2	-2.6 ±	0.1	1.4	±	0.1	
33	dex	5.5	±	0.2	-2.6 ±	0.1	2.1	±	0.1	0.7
33	peg	5.5	±	0.2	-2.6 ±	0.1	2.1	±	0.1	0.7

34	dil	5.4 ± 0.2	-3.1 ± 0.1	1.8 ± 0.1	
34	dex	7.3 ± 0.2	-3.1 ± 0.1	2.4 ± 0.1	0.6
34	peg	7.9 ± 0.2	-3.1 ± 0.1	2.6 ± 0.1	0.8
39	dil	3.5 ± 0.5	-2.3 ± 0.2	1.6 ± 0.3	
39	peg	4.9 ± 0.5	-2.3 ± 0.2	2.2 ± 0.3	0.6
40	dil	4.8 ± 0.2	-2.8 ± 0.1	1.7 ± 0.1	
40	dex	5.8 ± 0.2	-2.8 ± 0.1	2.1 ± 0.1	0.4
40	peg	6.1 ± 0.2	-2.8 ± 0.1	2.2 ± 0.1	0.5
41	dil	9.3 ± 0.5	-3.5 ± 0.1	2.7 ± 0.2	
41	dex	10.6 ± 0.5	-3.5 ± 0.1	3.0 ± 0.2	0.4
41	peg	10.1 ± 0.5	-3.5 ± 0.1	2.9 ± 0.2	0.2
42	dil	4.9 ± 0.3	-3.0 ± 0.1	1.6 ± 0.1	
42	dex	7.0 ± 0.3	-3.0 ± 0.1	2.3 ± 0.1	0.7
42	peg	7.0 ± 0.3	-3.0 ± 0.1	2.3 ± 0.1	0.7
44	dil	5.7 ± 0.2	-3.0 ± 0.1	1.9 ± 0.1	
44	dex	7.2 ± 0.2	-3.0 ± 0.1	2.4 ± 0.1	0.5
44	peg	7.2 ± 0.2	-3.0 ± 0.1	2.4 ± 0.1	0.5
45	dil	4.5 ± 0.2	-2.9 ± 0.1	1.5 ± 0.1	
45	dex	6.2 ± 0.2	-2.9 ± 0.1	2.1 ± 0.1	0.6
45	peg	6.6 ± 0.2	-2.9 ± 0.1	2.3 ± 0.1	0.7
46	dil	4.6 ± 0.2	-2.9 ± 0.1	1.6 ± 0.1	
46	dex	6.2 ± 0.2	-2.9 ± 0.1	2.1 ± 0.1	0.6
46	peg	6.7 ± 0.2	-2.9 ± 0.1	2.3 ± 0.1	0.7
47	dil	5.3 ± 0.2	-3.0 ± 0.1	1.8 ± 0.1	
47	dex	7.2 ± 0.2	-3.0 ± 0.1	2.4 ± 0.1	0.7
47	peg	7.3 ± 0.2	-3.0 ± 0.1	2.5 ± 0.1	0.7
48	dil	4.7 ± 0.2	-2.9 ± 0.1	1.7 ± 0.1	
48	dex	6.2 ± 0.2	-2.9 ± 0.1	2.2 ± 0.1	0.5
48	peg	6.6 ± 0.2	-2.9 ± 0.1	2.3 ± 0.1	0.7
49	dil	5.3 ± 0.2	-2.9 ± 0.1	1.8 ± 0.1	
49	dex	6.8 ± 0.2	-2.9 ± 0.1	2.3 ± 0.1	0.5
49	peg	7.6 ± 0.2	-2.9 ± 0.1	2.6 ± 0.1	0.8
50	dil	3.8 ± 0.3	-2.7 ± 0.1	1.4 ± 0.1	
50	peg	6.2 ± 0.3	-2.7 ± 0.1	2.3 ± 0.1	0.9
51	dil	6.3 ± 0.4	-3.0 ± 0.1	2.1 ± 0.1	
51	dex	7.7 ± 0.4	-3.0 ± 0.1	2.6 ± 0.2	0.5
51	peg	7.8 ± 0.4	-3.0 ± 0.1	2.6 ± 0.2	0.5
52	dil	6.1 ± 0.2	-2.9 ± 0.1	2.1 ± 0.1	
52	dex	7.6 ± 0.2	-2.9 ± 0.1	2.6 ± 0.1	0.5
52	peg	6.8 ± 0.2	-2.9 ± 0.1	2.4 ± 0.1	0.3
53	dil	5.4 ± 0.2	-2.6 ± 0.1	2.1 ± 0.1	
53	dex	6.1 ± 0.2	-2.6 ± 0.1	2.3 ± 0.1	0.2
53	peg	7.4 ± 0.2	-2.6 ± 0.1	2.8 ± 0.1	0.7
54	dil	5.6 ± 0.3	-2.8 ± 0.1	2.0 ± 0.1	
54	dex	6.2 ± 0.3	-2.8 ± 0.1	2.2 ± 0.1	0.2
54	peg	7.1 ± 0.3	-2.8 ± 0.1	2.6 ± 0.1	0.5

57	dil	5.1	±	0.3	-2.8	±	0.1	1.8	±	0.1	
57	dex	5.9	±	0.3	-2.8	±	0.1	2.1	±	0.1	0.3
57	peg	6.5	±	0.3	-2.8	±	0.1	2.3	±	0.1	0.5
59	dil	6.2	±	0.2	-3.1	±	0.1	2.0	±	0.1	
59	dex	7.7	±	0.2	-3.1	±	0.1	2.4	±	0.1	0.5
59	peg	7.7	±	0.2	-3.1	±	0.1	2.5	±	0.1	0.5
60	dil	6.0	±	0.2	-3.2	±	0.1	1.9	±	0.1	
60	dex	7.8	±	0.2	-3.2	±	0.1	2.5	±	0.1	0.6
60	peg	9.0	±	0.2	-3.2	±	0.1	2.9	±	0.1	0.9
61	dil	9.8	±	0.6	-3.5	±	0.2	2.8	±	0.2	
61	dex	10.8	±	0.6	-3.5	±	0.2	3.1	±	0.2	0.3
61	peg	11.1	±	0.6	-3.5	±	0.2	3.2	±	0.2	0.4
62	dil	5.9	±	0.2	-3.1	±	0.1	1.9	±	0.1	
62	dex	7.2	±	0.2	-3.1	±	0.1	2.3	±	0.1	0.4
62	peg	8.1	±	0.2	-3.1	±	0.1	2.6	±	0.1	0.7
63	dil	6.0	±	0.7	-3.0	±	0.2	2.0	±	0.3	
63	peg	7.8	±	0.8	-3.0	±	0.2	2.6	±	0.3	0.6
64	dil	5.2	±	0.2	-2.9	±	0.1	1.8	±	0.1	
64	dex	7.1	±	0.2	-2.9	±	0.1	2.5	±	0.1	0.6
64	peg	7.4	±	0.2	-2.9	±	0.1	2.6	±	0.1	0.8
65	dil	4.6	±	0.3	-2.6	±	0.1	1.7	±	0.1	
65	dex	6.0	±	0.3	-2.6	±	0.1	2.3	±	0.1	0.5
65	peg	6.8	±	0.3	-2.6	±	0.1	2.6	±	0.2	0.8
66	dil	4.4	±	0.3	-2.7	±	0.1	1.6	±	0.1	
66	dex	5.7	±	0.3	-2.7	±	0.1	2.1	±	0.1	0.5
66	peg	5.8	±	0.3	-2.7	±	0.1	2.2	±	0.1	0.5
67	dil	5.0	±	0.2	-2.4	±	0.1	2.1	±	0.1	
67	dex	6.1	±	0.3	-2.4	±	0.1	2.6	±	0.1	0.4
67	peg	6.5	±	0.3	-2.4	±	0.1	2.7	±	0.1	0.6

Supplementary Table 3

Results obtained for methine group protons (CH, labeled as HA, HB, HG) using linear extrapolation of the transition region ranging from c = 2 to 4 M urea in two-dimensional heteronuclear ^{1}H-^{13}C HSQC spectra. Folding-to-unfolding transitions have been acquired under three different conditions abbreviated using dil (dilute conditions), dex (c = for 120 g/L Dex20) and peg (c = for 120 g/L PEG1). Each proton group being present in a single residue was fitted using a global value for the cooperativity of folding, m, applying all three different experimental conditions as discussed previously [5]. Values for ΔC_M are shown in Figure 3*A, B* and are graphically highlighted in Supplementary Figure 17.

residue		$\Delta G^0_{N \leftrightarrow U}$ / kJ*mol^{-1}	m / kJ*(mol*M)$^{-1}$	C_M / M	ΔC_M / M
L2 HA	dil	6.7 ± 0.3	-3.0 ± 0.1	2.3 ± 0.1	
L2 HA	dex	7.7 ± 0.3	-3.0 ± 0.1	2.6 ± 0.1	0.3
L2 HA	peg	7.6 ± 0.3	-3.0 ± 0.1	2.6 ± 0.1	0.3
L2 HG	dil	9.6 ± 0.6	-4.8 ± 0.2	2.0 ± 0.1	
L2 HG	dex	11.9 ± 0.6	-4.8 ± 0.2	2.5 ± 0.2	0.5
L2 HG	peg	11.6 ± 0.6	-4.8 ± 0.2	2.4 ± 0.2	0.4
E3 HA	dil	5.5 ± 0.2	-2.9 ± 0.1	1.9 ± 0.1	
E3 HA	dex	7.3 ± 0.2	-2.9 ± 0.1	2.5 ± 0.1	0.6
E3 HA	peg	6.9 ± 0.2	-2.9 ± 0.1	2.4 ± 0.1	0.5
K5 HA	dil	6.6 ± 0.2	-3.1 ± 0.1	2.1 ± 0.1	
K5 HA	dex	8.5 ± 0.2	-3.1 ± 0.1	2.7 ± 0.1	0.6
K5 HA	peg	7.6 ± 0.2	-3.1 ± 0.1	2.4 ± 0.1	0.3
V6 HA	dil	6.4 ± 0.3	-3.0 ± 0.1	2.1 ± 0.1	
V6 HA	dex	8.1 ± 0.3	-3.0 ± 0.1	2.7 ± 0.1	0.6
V6 HA	peg	7.0 ± 0.3	-3.0 ± 0.1	2.4 ± 0.1	0.2
V6 HB	dil	5.0 ± 0.6	-2.4 ± 0.2	2.1 ± 0.3	
V6 HB	peg	6.0 ± 0.6	-2.4 ± 0.2	2.5 ± 0.3	0.4
K7 HA	dil	9.3 ± 0.3	-3.1 ± 0.1	3.0 ± 0.1	
K7 HA	dex	11.1 ± 0.3	-3.1 ± 0.1	3.6 ± 0.1	0.6
K7 HA	peg	9.5 ± 0.3	-3.1 ± 0.1	3.1 ± 0.1	0.1
W8 HA	dil	7.5 ± 0.4	-2.9 ± 0.1	2.5 ± 0.2	
W8 HA	dex	8.7 ± 0.4	-2.9 ± 0.1	3.0 ± 0.2	0.4
W8 HA	peg	7.6 ± 0.4	-2.9 ± 0.1	2.6 ± 0.2	0.0
F9 HA	dil	6.3 ± 0.3	-2.6 ± 0.1	2.4 ± 0.1	
F9 HA	dex	7.9 ± 0.3	-2.6 ± 0.1	3.1 ± 0.2	0.6
F9 HA	peg	6.7 ± 0.3	-2.6 ± 0.1	2.6 ± 0.2	0.2
N10 HA	dil	7.3 ± 0.4	-3.1 ± 0.1	2.3 ± 0.1	
N10 HA	dex	8.4 ± 0.4	-3.1 ± 0.1	2.7 ± 0.2	0.3
N10 HA	peg	8.1 ± 0.4	-3.1 ± 0.1	2.6 ± 0.2	0.3
E12 HA	dil	6.1 ± 0.2	-3.0 ± 0.1	2.0 ± 0.1	
E12 HA	dex	7.9 ± 0.2	-3.0 ± 0.1	2.6 ± 0.1	0.6
E12 HA	peg	6.8 ± 0.3	-3.0 ± 0.1	2.3 ± 0.1	0.2

F15 HA	dil	7.2	±	0.3	-3.1	±	0.1	2.3	±	0.1	
F15 HA	dex	8.9	±	0.3	-3.1	±	0.1	2.9	±	0.1	0.6
F15 HA	peg	7.8	±	0.3	-3.1	±	0.1	2.5	±	0.1	0.2
F17 HA	dil	6.4	±	0.3	-2.9	±	0.1	2.2	±	0.1	
F17 HA	dex	7.8	±	0.3	-2.9	±	0.1	2.7	±	0.1	0.5
F17 HA	peg	7.0	±	0.3	-2.9	±	0.1	2.5	±	0.1	0.2
I18 HA	dil	10.0	±	0.4	-3.7	±	0.1	2.7	±	0.1	
I18 HA	dex	11.8	±	0.4	-3.7	±	0.1	3.2	±	0.1	0.5
I18 HA	peg	10.5	±	0.4	-3.7	±	0.1	2.8	±	0.1	0.1
I18 HB	dil	7.6	±	0.2	-3.2	±	0.1	2.4	±	0.1	
I18 HB	dex	9.4	±	0.2	-3.2	±	0.1	3.0	±	0.1	0.6
I18 HB	peg	8.6	±	0.3	-3.2	±	0.1	2.7	±	0.1	0.3
E19 HA	dil	7.1	±	0.4	-3.3	±	0.1	2.2	±	0.1	
E19 HA	dex	8.8	±	0.4	-3.3	±	0.1	2.7	±	0.2	0.5
E19 HA	peg	9.1	±	0.4	-3.3	±	0.1	2.8	±	0.2	0.6
V20 HA	dil	6.6	±	0.2	-3.0	±	0.1	2.2	±	0.1	
V20 HA	dex	8.3	±	0.2	-3.0	±	0.1	2.8	±	0.1	0.6
V20 HA	peg	7.6	±	0.2	-3.0	±	0.1	2.5	±	0.1	0.3
V20 HB	dil	7.2	±	0.3	-3.4	±	0.1	2.1	±	0.1	
V20 HB	dex	8.9	±	0.3	-3.4	±	0.1	2.6	±	0.1	0.5
V20 HB	peg	8.5	±	0.3	-3.4	±	0.1	2.5	±	0.1	0.4
E21 HA	dil	4.9	±	0.2	-2.7	±	0.1	1.9	±	0.1	
E21 HA	dex	6.5	±	0.2	-2.7	±	0.1	2.4	±	0.1	0.6
E21 HA	peg	6.2	±	0.2	-2.7	±	0.1	2.3	±	0.1	0.5
Q23 HA	dil	5.6	±	0.3	-3.0	±	0.1	1.9	±	0.1	
Q23 HA	dex	7.6	±	0.3	-3.0	±	0.1	2.6	±	0.1	0.7
Q23 HA	peg	6.8	±	0.3	-3.0	±	0.1	2.3	±	0.1	0.4
D25 HA	dil	7.6	±	0.3	-3.5	±	0.1	2.2	±	0.1	
D25 HA	dex	9.6	±	0.3	-3.5	±	0.1	2.8	±	0.1	0.6
D25 HA	peg	8.6	±	0.3	-3.5	±	0.1	2.5	±	0.1	0.3
V26 HA	dil	4.9	±	0.4	-2.2	±	0.1	2.2	±	0.2	
V26 HA	peg	5.4	±	0.4	-2.2	±	0.1	2.4	±	0.2	0.2
V26 HB	dil	7.5	±	0.3	-3.2	±	0.1	2.3	±	0.1	
V26 HB	dex	9.2	±	0.3	-3.2	±	0.1	2.8	±	0.1	0.5
V26 HB	peg	8.5	±	0.3	-3.2	±	0.1	2.6	±	0.1	0.3
V28 HB	dil	5.6	±	0.3	-2.8	±	0.1	2.0	±	0.1	
V28 HB	dex	8.0	±	0.3	-2.8	±	0.1	2.8	±	0.1	0.8
V28 HB	peg	6.6	±	0.3	-2.8	±	0.1	2.4	±	0.1	0.4
H29 HA	dil	4.8	±	0.3	-2.6	±	0.1	1.8	±	0.1	
H29 HA	dex	6.6	±	0.3	-2.6	±	0.1	2.5	±	0.1	0.7
H29 HA	peg	5.7	±	0.3	-2.6	±	0.1	2.2	±	0.1	0.4
F30 HA	dil	8.0	±	0.5	-3.5	±	0.2	2.3	±	0.2	
F30 HA	dex	10.5	±	0.5	-3.5	±	0.2	3.0	±	0.2	0.7
F30 HA	peg	9.2	±	0.5	-3.5	±	0.2	2.6	±	0.2	0.3
S31 HA	dil	7.9	±	0.4	-3.2	±	0.1	2.5	±	0.2	
S31 HA	dex	9.9	±	0.4	-3.2	±	0.1	3.1	±	0.2	0.6
S31 HA	peg	8.3	±	0.4	-3.2	±	0.1	2.6	±	0.2	0.1

A32 HA	dil	5.5	±	0.3	-2.4 ±	0.1	2.3	±	0.2	
A32 HA	dex	7.1	±	0.3	-2.4 ±	0.1	2.9	±	0.2	0.6
A32 HA	peg	5.9	±	0.3	-2.4 ±	0.1	2.5	±	0.2	0.2
I33 HB	dil	5.8	±	0.2	-3.1 ±	0.1	1.8	±	0.1	
I33 HB	dex	7.9	±	0.2	-3.1 ±	0.1	2.5	±	0.1	0.7
I33 HB	peg	7.5	±	0.2	-3.1 ±	0.1	2.4	±	0.1	0.5
K39 HA	dil	4.7	±	0.3	-2.2 ±	0.1	2.1	±	0.2	
K39 HA	dex	6.1	±	0.3	-2.2 ±	0.1	2.7	±	0.2	0.6
K39 HA	peg	5.7	±	0.3	-2.2 ±	0.1	2.5	±	0.2	0.4
L41 HG	dil	5.0	±	0.2	-3.0 ±	0.1	1.7	±	0.1	
L41 HG	dex	7.2	±	0.2	-3.0 ±	0.1	2.4	±	0.1	0.7
L41 HG	peg	6.8	±	0.2	-3.0 ±	0.1	2.3	±	0.1	0.6
E43 HA	dil	5.5	±	0.2	-2.9 ±	0.1	1.9	±	0.1	
E43 HA	dex	7.3	±	0.2	-2.9 ±	0.1	2.5	±	0.1	0.6
E43 HA	peg	7.2	±	0.3	-2.9 ±	0.1	2.4	±	0.1	0.6
A46 HA	dil	8.0	±	0.4	-3.0 ±	0.1	2.7	±	0.2	
A46 HA	dex	10.1	±	0.4	-3.0 ±	0.1	3.4	±	0.2	0.7
A46 HA	peg	8.9	±	0.4	-3.0 ±	0.1	3.0	±	0.2	0.3
S48 HA	dil	6.0	±	0.3	-2.9 ±	0.1	2.1	±	0.1	
S48 HA	dex	7.4	±	0.3	-2.9 ±	0.1	2.6	±	0.1	0.5
S48 HA	peg	7.0	±	0.3	-2.9 ±	0.1	2.4	±	0.1	0.3
F49 HA	dil	6.8	±	0.3	-2.9 ±	0.1	2.3	±	0.1	
F49 HA	dex	8.1	±	0.3	-2.9 ±	0.1	2.7	±	0.1	0.4
F49 HA	peg	7.7	±	0.3	-2.9 ±	0.1	2.6	±	0.1	0.3
E50 HA	dil	8.1	±	0.2	-3.3 ±	0.1	2.5	±	0.1	
E50 HA	dex	9.4	±	0.2	-3.3 ±	0.1	2.9	±	0.1	0.4
E50 HA	peg	8.4	±	0.2	-3.3 ±	0.1	2.6	±	0.1	0.1
I51 HA	dil	8.8	±	0.3	-3.0 ±	0.1	3.0	±	0.1	
I51 HA	dex	10.4	±	0.3	-3.0 ±	0.1	3.5	±	0.2	0.5
I51 HA	peg	9.0	±	0.3	-3.0 ±	0.1	3.0	±	0.2	0.1
I51 HB	dil	7.5	±	0.3	-3.2 ±	0.1	2.3	±	0.1	
I51 HB	dex	9.4	±	0.3	-3.2 ±	0.1	2.9	±	0.1	0.6
I51 HB	peg	8.9	±	0.3	-3.2 ±	0.1	2.8	±	0.1	0.4
V52 HA	dil	8.1	±	0.3	-3.3 ±	0.1	2.4	±	0.1	
V52 HA	dex	9.7	±	0.3	-3.3 ±	0.1	2.9	±	0.1	0.5
V52 HA	peg	8.4	±	0.3	-3.3 ±	0.1	2.5	±	0.1	0.1
V52 HB	dil	7.6	±	0.3	-3.1 ±	0.1	2.4	±	0.1	
V52 HB	dex	8.7	±	0.3	-3.1 ±	0.1	2.8	±	0.1	0.4
V52 HB	peg	8.4	±	0.3	-3.1 ±	0.1	2.7	±	0.1	0.3
R56 HA	dil	7.0	±	0.1	-3.0 ±	0.0	2.3	±	0.1	
R56 HA	dex	9.0	±	0.1	-3.0 ±	0.0	3.0	±	0.1	0.7
R56 HA	peg	7.8	±	0.1	-3.0 ±	0.0	2.6	±	0.1	0.3
P58 HA	dil	7.0	±	0.5	-2.7 ±	0.2	2.6	±	0.2	
P58 HA	dex	8.6	±	0.5	-2.7 ±	0.2	3.2	±	0.3	0.6
P58 HA	peg	7.1	±	0.5	-2.7 ±	0.2	2.6	±	0.2	0.0
A60 HA	dil	6.5	±	0.4	-2.9 ±	0.1	2.2	±	0.2	
A60 HA	dex	8.7	±	0.4	-2.9 ±	0.1	2.9	±	0.2	0.7
A60 HA	peg	7.9	±	0.4	-2.9 ±	0.1	2.7	±	0.2	0.5

A61 HA	dil	5.7	±	0.3	-2.9	±	0.1	2.0	±	0.1	
A61 HA	dex	7.7	±	0.3	-2.9	±	0.1	2.7	±	0.1	0.7
A61 HA	peg	6.8	±	0.3	-2.9	±	0.1	2.4	±	0.1	0.4
V63 HA	dil	6.4	±	0.3	-3.1	±	0.1	2.0	±	0.1	
V63 HA	dex	8.0	±	0.3	-3.1	±	0.1	2.6	±	0.1	0.5
V63 HA	peg	7.5	±	0.3	-3.1	±	0.1	2.4	±	0.1	0.4
V63 HB	dil	5.4	±	0.4	-2.9	±	0.1	1.8	±	0.2	
V63 HB	dex	6.9	±	0.4	-2.9	±	0.1	2.4	±	0.2	0.5
V63 HB	peg	6.4	±	0.4	-2.9	±	0.1	2.2	±	0.2	0.4
T64 HA	dil	4.9	±	0.4	-2.7	±	0.1	1.8	±	0.2	
T64 HA	dex	6.6	±	0.4	-2.7	±	0.1	2.5	±	0.2	0.6
T64 HA	peg	6.3	±	0.4	-2.7	±	0.1	2.4	±	0.2	0.5
T64 HB	dil	4.4	±	0.3	-2.6	±	0.1	1.7	±	0.2	
T64 HB	peg	9.0	±	0.0	-2.6	±	0.1	2.4	±	0.2	0.7

Supplementary Table 4

Results obtained for methylene group protons (CH_2, labeled as HB, HB1, HB2, HA, HA1, HA2, HG1, HG2) using linear extrapolation of the transition region ranging from c = 2 to 4 M urea in two-dimensional heteronuclear ^{1}H-^{13}C HSQC spectra. Folding-to-unfolding transitions have been acquired under three different conditions abbreviated using dil (dilute conditions), dex (c = 120 g/L Dex20) and peg (c = 120 g/L PEG1). Each proton group being present in a single residue was fitted using a global value for the cooperativity of folding, m, applying all three different experimental conditions as discussed previously [5]. Values for ΔC_M are shown in Figure 3*C*, *D* and are graphically highlighted in Supplementary Figure 17.

residue		$\Delta G^0_{N\leftrightarrow U}$ / kJ*mol^{-1}			m / kJ*(mol*M)$^{-1.}$			C_M / M			ΔC_M / M
L2 HB1	dil	9.3	±	0.5	-3.5	±	0.2	2.6	±	0.2	
L2 HB1	dex	10.9	±	0.5	-3.5	±	0.2	3.1	±	0.2	0.4
L2 HB1	peg	10.2	±	0.5	-3.5	±	0.2	2.9	±	0.2	0.3
L2 HB2	dil	9.3	±	0.4	-3.6	±	0.1	2.6	±	0.2	
L2 HB2	dex	11.1	±	0.4	-3.6	±	0.1	3.1	±	0.2	0.5
L2 HB2	peg	10.4	±	0.5	-3.6	±	0.1	2.9	±	0.2	0.3
K5 HB1	dil	7.0	±	0.6	-3.2	±	0.2	2.2	±	0.2	
K5 HB1	dex	8.3	±	0.6	-3.2	±	0.2	2.6	±	0.2	0.4
K5 HB1	peg	7.6	±	0.6	-3.2	±	0.2	2.4	±	0.2	0.2
K5 HB2	dil	5.4	±	0.7	-2.8	±	0.2	1.9	±	0.3	
K5 HB2	dex	7.0	±	0.7	-2.8	±	0.2	2.5	±	0.3	0.6
K5 HB2	peg	6.1	±	0.7	-2.8	±	0.2	2.2	±	0.3	0.3
K7 HB1	dil	6.7	±	0.5	-2.9	±	0.2	2.3	±	0.2	
K7 HB1	dex	9.0	±	0.5	-2.9	±	0.2	3.2	±	0.2	0.8
K7 HB1	peg	7.5	±	0.5	-2.9	±	0.2	2.6	±	0.2	0.3
K7 HB2	dil	7.7	±	0.3	-3.1	±	0.1	2.5	±	0.1	
K7 HB2	dex	9.5	±	0.3	-3.1	±	0.1	3.0	±	0.1	0.6
K7 HB2	peg	8.1	±	0.3	-3.1	±	0.1	2.6	±	0.1	0.2
W8 HB1	dil	6.0	±	0.6	-2.8	±	0.2	2.2	±	0.3	
W8 HB1	dex	7.3	±	0.6	-2.8	±	0.2	2.7	±	0.3	0.5
W8 HB1	peg	5.9	±	0.6	-2.8	±	0.2	2.1	±	0.3	0.0
W8 HB2	dil	6.6	±	0.7	-3.0	±	0.2	2.2	±	0.3	
W8 HB2	dex	8.7	±	0.7	-3.0	±	0.2	2.9	±	0.3	0.7
W8 HB2	peg	7.8	±	0.7	-3.0	±	0.2	2.6	±	0.3	0.4
N10 HB	dil	5.5	±	0.4	-2.4	±	0.1	2.3	±	0.2	
N10 HB	dex	7.9	±	0.4	-2.4	±	0.1	3.2	±	0.2	1.0
N10 HB	peg	7.0	±	0.4	-2.4	±	0.1	2.9	±	0.2	0.6
S11 HB	dil	5.6	±	0.6	-2.8	±	0.2	2.0	±	0.3	
S11 HB	dex	7.3	±	0.6	-2.8	±	0.2	2.6	±	0.3	0.6
S11 HB	peg	6.4	±	0.7	-2.8	±	0.2	2.3	±	0.3	0.3
K13 HB	dil	6.8	±	0.3	-2.9	±	0.1	2.3	±	0.1	
K13 HB	dex	8.2	±	0.3	-2.9	±	0.1	2.8	±	0.1	0.5
K13 HB	peg	7.0	±	0.3	-2.9	±	0.1	2.4	±	0.1	0.1

K13 HG1	dil	7.4	±	0.3	-3.1	±	0.1	2.4	±	0.1	
K13 HG1	dex	9.3	±	0.3	-3.1	±	0.1	3.0	±	0.1	0.6
K13 HG1	peg	7.7	±	0.3	-3.1	±	0.1	2.5	±	0.1	0.1
K13 HG2	dil	7.5	±	0.3	-3.3	±	0.1	2.3	±	0.1	
K13 HG2	dex	9.7	±	0.3	-3.3	±	0.1	2.9	±	0.1	0.7
K13 HG2	peg	8.9	±	0.3	-3.3	±	0.1	2.7	±	0.1	0.4
G14 HA	dil	6.5	±	0.3	-2.9	±	0.1	2.2	±	0.1	
G14 HA	dex	8.5	±	0.3	-2.9	±	0.1	2.9	±	0.1	0.7
G14 HA	peg	7.4	±	0.3	-2.9	±	0.1	2.5	±	0.1	0.3
F17 HB1	dil	8.0	±	0.5	-3.2	±	0.2	2.5	±	0.2	
F17 HB1	dex	9.7	±	0.5	-3.2	±	0.2	3.0	±	0.2	0.5
F17 HB1	peg	7.5	±	0.5	-3.2	±	0.2	2.3	±	0.2	-0.1
F17 HB2	dil	7.9	±	0.4	-3.1	±	0.1	2.5	±	0.2	
F17 HB2	dex	9.6	±	0.4	-3.1	±	0.1	3.1	±	0.2	0.5
F17 HB2	peg	7.7	±	0.4	-3.1	±	0.1	2.5	±	0.2	-0.1
I18 HG12	dil	7.3	±	0.3	-3.2	±	0.1	2.3	±	0.1	
I18 HG12	dex	9.2	±	0.3	-3.2	±	0.1	2.9	±	0.1	0.6
I18 HG12	peg	8.1	±	0.3	-3.2	±	0.1	2.5	±	0.1	0.2
I18 HG13	dil	7.0	±	0.3	-3.3	±	0.1	2.1	±	0.1	
I18 HG13	dex	8.4	±	0.3	-3.3	±	0.1	2.5	±	0.1	0.4
I18 HG13	peg	7.3	±	0.3	-3.3	±	0.1	2.2	±	0.1	0.1
G22 HA	dil	7.7	±	0.5	-3.1	±	0.2	2.5	±	0.2	
G22 HA	dex	9.2	±	0.5	-3.1	±	0.2	2.9	±	0.2	0.5
G22 HA	peg	8.7	±	0.5	-3.1	±	0.2	2.8	±	0.2	0.3
H29 HB1	dil	7.0	±	0.2	-2.9	±	0.1	2.4	±	0.1	
H29 HB1	dex	8.7	±	0.2	-2.9	±	0.1	3.0	±	0.1	0.6
H29 HB1	peg	7.7	±	0.2	-2.9	±	0.1	2.7	±	0.1	0.3
H29 HB2	dil	6.7	±	0.3	-2.8	±	0.1	2.4	±	0.1	
H29 HB2	dex	8.7	±	0.3	-2.8	±	0.1	3.1	±	0.1	0.7
H29 HB2	peg	7.6	±	0.3	-2.8	±	0.1	2.7	±	0.1	0.3
F30 HB1	dil	7.9	±	0.5	-3.1	±	0.2	2.6	±	0.2	
F30 HB1	dex	9.6	±	0.5	-3.1	±	0.2	3.1	±	0.2	0.6
F30 HB1	peg	7.9	±	0.5	-3.1	±	0.2	2.6	±	0.2	0.0
F30 HB2	dil	8.0	±	0.4	-3.2	±	0.1	2.5	±	0.2	
F30 HB2	dex	9.1	±	0.4	-3.2	±	0.1	2.9	±	0.2	0.3
F30 HB2	peg	7.8	±	0.4	-3.2	±	0.1	2.5	±	0.2	-0.1
S31 HB	dil	7.0	±	0.5	-2.9	±	0.1	2.4	±	0.2	
S31 HB	dex	9.1	±	0.5	-2.9	±	0.1	3.1	±	0.2	0.8
S31 HB	peg	7.4	±	0.5	-2.9	±	0.1	2.5	±	0.2	0.2
I33 HG12	dil	7.0	±	0.3	-3.2	±	0.1	2.2	±	0.1	
I33 HG12	dex	9.6	±	0.3	-3.2	±	0.1	3.0	±	0.1	0.8
I33 HG12	peg	8.3	±	0.3	-3.2	±	0.1	2.6	±	0.1	0.4
I33 HG13	dil	7.5	±	0.9	-3.5	±	0.3	2.2	±	0.3	
I33 HG13	dex	9.6	±	0.9	-3.5	±	0.3	2.8	±	0.4	0.6
I33 HG13	peg	7.9	±	0.9	-3.5	±	0.3	2.3	±	0.3	0.1
G35 HA	dil	5.7	±	0.2	-2.6	±	0.1	2.2	±	0.1	
G35 HA	dex	7.3	±	0.2	-2.6	±	0.1	2.8	±	0.1	0.6
G35 HA	peg	6.7	±	0.2	-2.6	±	0.1	2.5	±	0.1	0.4

E36 HB1	dil	5.6	±	0.2	-2.8	±	0.1	2.0	±	0.1	
E36 HB1	dex	7.3	±	0.2	-2.8	±	0.1	2.6	±	0.1	0.6
E36 HB1	peg	7.1	±	0.2	-2.8	±	0.1	2.5	±	0.1	0.5
E36 HB2	dil	5.5	±	0.2	-2.7	±	0.1	2.0	±	0.1	
E36 HB2	dex	7.0	±	0.2	-2.7	±	0.1	2.6	±	0.1	0.6
E36 HB2	peg	6.6	±	0.2	-2.7	±	0.1	2.4	±	0.1	0.4
G37 HA	dil	4.6	±	0.2	-2.3	±	0.1	2.0	±	0.1	
G37 HA	dex	6.1	±	0.2	-2.3	±	0.1	2.7	±	0.1	0.6
G37 HA	peg	5.2	±	0.2	-2.3	±	0.1	2.3	±	0.1	0.3
F38 HB	dil	1.6	±	0.2	-1.5	±	0.1	1.0	±	0.2	
F38 HB	dex	3.9	±	0.2	-1.5	±	0.1	2.6	±	0.2	1.5
F38 HB	peg	1.8	±	0.3	-1.5	±	0.1	1.2	±	0.2	0.2
L41 HB1	dil	6.5	±	0.3	-2.8	±	0.1	2.3	±	0.1	
L41 HB1	dex	8.2	±	0.3	-2.8	±	0.1	2.9	±	0.1	0.6
L41 HB1	peg	7.0	±	0.3	-2.8	±	0.1	2.5	±	0.1	0.2
L41 HB2	dil	8.2	±	0.4	-3.2	±	0.1	2.5	±	0.1	
L41 HB2	dex	9.8	±	0.4	-3.2	±	0.1	3.0	±	0.2	0.5
L41 HB2	peg	8.7	±	0.4	-3.2	±	0.1	2.7	±	0.2	0.1
S48 HB	dil	4.8	±	0.3	-2.6	±	0.1	1.9	±	0.1	
S48 HB	dex	6.5	±	0.3	-2.6	±	0.1	2.5	±	0.1	0.6
S48 HB	peg	5.8	±	0.3	-2.6	±	0.1	2.2	±	0.1	0.4
F49 HB	dil	6.9	±	0.2	-3.1	±	0.1	2.2	±	0.1	
F49 HB	dex	8.6	±	0.2	-3.1	±	0.1	2.8	±	0.1	0.5
F49 HB	peg	7.5	±	0.3	-3.1	±	0.1	2.4	±	0.1	0.2
I51 HG13	dil	5.9	±	0.3	-2.8	±	0.1	2.1	±	0.1	
I51 HG13	dex	8.0	±	0.3	-2.8	±	0.1	2.8	±	0.1	0.8
I51 HG13	peg	7.8	±	0.3	-2.8	±	0.1	2.7	±	0.1	0.7
G54 HA	dil	8.0	±	0.3	-3.1	±	0.1	2.6	±	0.1	
G54 HA	dex	9.6	±	0.3	-3.1	±	0.1	3.1	±	0.1	0.5
G54 HA	peg	8.5	±	0.3	-3.1	±	0.1	2.7	±	0.1	0.1
N55 HB	dil	5.6	±	0.1	-2.5	±	0.0	2.2	±	0.1	
N55 HB	dex	7.7	±	0.1	-2.5	±	0.0	3.1	±	0.1	0.9
N55 HB	peg	6.8	±	0.1	-2.5	±	0.0	2.7	±	0.1	0.5
G57 HA1	dil	8.0	±	0.4	-3.2	±	0.1	2.5	±	0.2	
G57 HA1	dex	9.6	±	0.4	-3.2	±	0.1	3.0	±	0.2	0.5
G57 HA1	peg	8.2	±	0.4	-3.2	±	0.1	2.5	±	0.2	0.1
G57 HA2	dil	7.9	±	0.4	-2.9	±	0.1	2.7	±	0.2	
G57 HA2	dex	9.0	±	0.4	-2.9	±	0.1	3.1	±	0.2	0.4
G57 HA2	peg	8.0	±	0.4	-2.9	±	0.1	2.7	±	0.2	0.0
P58 HB	dil	6.9	±	0.3	-3.1	±	0.1	2.2	±	0.1	
P58 HB	dex	8.4	±	0.3	-3.1	±	0.1	2.7	±	0.1	0.5
P58 HB	peg	7.0	±	0.3	-3.1	±	0.1	2.3	±	0.1	0.1
P58 HG1	dil	7.5	±	0.3	-3.4	±	0.1	2.2	±	0.1	
P58 HG1	dex	9.6	±	0.3	-3.4	±	0.1	2.8	±	0.1	0.6
P58 HG1	peg	8.2	±	0.3	-3.4	±	0.1	2.4	±	0.1	0.2
P58 HG2	dil	6.8	±	0.4	-3.1	±	0.1	2.2	±	0.1	
P58 HG2	dex	8.6	±	0.4	-3.1	±	0.1	2.8	±	0.2	0.6
P58 HG2	peg	8.4	±	0.4	-3.1	±	0.1	2.7	±	0.2	0.5

N62 HB1	dil	6.5	±	0.2	-2.9	±	0.1	2.2	±	0.1	
N62 HB1	dex	8.2	±	0.2	-2.9	±	0.1	2.8	±	0.1	0.6
N62 HB1	peg	7.6	±	0.2	-2.9	±	0.1	2.6	±	0.1	0.4
N62 HB2	dil	6.1	±	0.2	-2.9	±	0.1	2.1	±	0.1	
N62 HB2	dex	7.8	±	0.2	-2.9	±	0.1	2.7	±	0.1	0.6
N62 HB2	peg	7.3	±	0.2	-2.9	±	0.1	2.6	±	0.1	0.4

Supplementary Table 5.

Results obtained for methyl group protons (CH_3, labeled as HB, HD, HD1, HD2, HG, HG1, HG2) using linear extrapolation of the transition region ranging from c = 2 to 4 M urea in two-dimensional heteronuclear ^{1}H-^{13}C HSQC spectra. Folding-to-unfolding transitions have been acquired under three different conditions abbreviated using dil (dilute conditions), dex (c = for 120 g/L Dex20) and peg (c = for 120 g/L PEG1). Each proton group being present in a single residue was fitted using a global value for the cooperativity of folding, m, applying all three different experimental conditions as discussed previously [5]. Values for ΔC_M are shown in Figure 3*E*, *F* and are graphically highlighted in Supplementary Figure 17.

residue		$\Delta G^0_{N \leftrightarrow U}$ / kJ*mol^{-1}			m / kJ*(mol*M)$^{-1.}$			C_M / M			ΔC_M / M
L2 HD1	dil	5.5	±	0.3	-3.1	±	0.1	1.8	±	0.1	
L2 HD1	dex	7.5	±	0.3	-3.1	±	0.1	2.4	±	0.1	0.6
L2 HD1	peg	7.1	±	0.3	-3.1	±	0.1	2.3	±	0.1	0.5
L2 HD2	dil	4.7	±	0.2	-2.7	±	0.1	1.7	±	0.1	
L2 HD2	dex	6.5	±	0.2	-2.7	±	0.1	2.4	±	0.1	0.7
L2 HD2	peg	7.0	±	0.2	-2.7	±	0.1	2.6	±	0.1	0.9
V6 HG1	dil	8.4	±	0.6	-4.0	±	0.2	2.1	±	0.2	
V6 HG1	dex	11.1	±	0.6	-4.0	±	0.2	2.8	±	0.2	0.7
V6 HG1	peg	10.6	±	0.6	-4.0	±	0.2	2.6	±	0.2	0.5
V6 HG2	dil	7.8	±	0.4	-2.6	±	0.1	3.0	±	0.2	
V6 HG2	dex	9.8	±	0.4	-2.6	±	0.1	3.7	±	0.2	0.8
V6 HG2	peg	8.4	±	0.4	-2.6	±	0.1	3.2	±	0.2	0.2
I18 HD	dil	8.0	±	0.3	-2.9	±	0.1	2.7	±	0.1	
I18 HD	dex	10.1	±	0.3	-2.9	±	0.1	3.4	±	0.1	0.7
I18 HD	peg	9.0	±	0.3	-2.9	±	0.1	3.0	±	0.1	0.3
V20 HG1	dil	8.8	±	0.2	-3.2	±	0.1	2.8	±	0.1	
V20 HG1	dex	10.7	±	0.2	-3.2	±	0.1	3.4	±	0.1	0.6
V20 HG1	peg	8.2	±	0.2	-3.2	±	0.1	2.6	±	0.1	-0.2
V20 HG2	dil	6.1	±	0.4	-3.0	±	0.1	2.0	±	0.1	
V20 HG2	dex	7.9	±	0.4	-3.0	±	0.1	2.6	±	0.2	0.6
V20 HG2	peg	9.5	±	0.4	-3.0	±	0.1	3.2	±	0.2	1.1
V26 HG1	dil	4.9	±	0.2	-2.6	±	0.1	1.9	±	0.1	
V26 HG1	dex	6.4	±	0.2	-2.6	±	0.1	2.5	±	0.1	0.6
V26 HG1	peg	6.1	±	0.2	-2.6	±	0.1	2.4	±	0.1	0.5
V26 HG2	dil	6.1	±	0.2	-2.9	±	0.1	2.1	±	0.1	
V26 HG2	dex	8.0	±	0.2	-2.9	±	0.1	2.8	±	0.1	0.7
V26 HG2	peg	7.1	±	0.2	-2.9	±	0.1	2.5	±	0.1	0.3
V28 HG1	dil	6.8	±	0.2	-3.0	±	0.1	2.3	±	0.1	
V28 HG1	dex	9.1	±	0.2	-3.0	±	0.1	3.0	±	0.1	0.8
V28 HG1	peg	8.0	±	0.2	-3.0	±	0.1	2.7	±	0.1	0.4
V28 HG2	dil	7.5	±	0.3	-3.1	±	0.1	2.4	±	0.1	
V28 HG2	dex	9.3	±	0.3	-3.1	±	0.1	3.0	±	0.1	0.6
V28 HG2	peg	8.2	±	0.3	-3.1	±	0.1	2.6	±	0.1	0.2

A32 HB	dil	5.9	±	0.3	-3.0	±	0.1	2.0	±	0.1	
A32 HB	dex	7.9	±	0.3	-3.0	±	0.1	2.6	±	0.1	0.6
A32 HB	peg	7.0	±	0.3	-3.0	±	0.1	2.3	±	0.1	0.4
I33 HD	dil	5.7	±	0.3	-2.9	±	0.1	2.0	±	0.1	
I33 HD	dex	7.7	±	0.3	-2.9	±	0.1	2.6	±	0.1	0.7
I33 HD	peg	7.0	±	0.3	-2.9	±	0.1	2.4	±	0.1	0.4
T40 HG	dil	6.3	±	0.3	-3.0	±	0.1	2.1	±	0.1	
T40 HG	dex	8.4	±	0.3	-3.0	±	0.1	2.8	±	0.1	0.7
T40 HG	peg	7.1	±	0.3	-3.0	±	0.1	2.4	±	0.1	0.3
L41 HD	dil	6.0	±	0.5	-3.3	±	0.2	1.8	±	0.2	
L41 HD	dex	9.4	±	0.5	-3.3	±	0.2	2.9	±	0.2	1.0
L41 HD	peg	8.8	±	0.5	-3.3	±	0.2	2.7	±	0.2	0.9
A46 HB	dil	8.0	±	0.4	-3.0	±	0.1	2.6	±	0.2	
A46 HB	dex	9.9	±	0.3	-3.0	±	0.1	3.3	±	0.2	0.6
A46 HB	peg	9.1	±	0.4	-3.0	±	0.1	3.0	±	0.2	0.4
V47 HG1	dil	5.4	±	0.2	-2.8	±	0.1	1.9	±	0.1	
V47 HG1	dex	7.0	±	0.2	-2.8	±	0.1	2.5	±	0.1	0.6
V47 HG1	peg	6.8	±	0.2	-2.8	±	0.1	2.5	±	0.1	0.5
V47 HG2	dil	5.1	±	0.2	-2.8	±	0.1	1.8	±	0.1	
V47 HG2	dex	6.8	±	0.2	-2.8	±	0.1	2.5	±	0.1	0.6
V47 HG2	peg	6.8	±	0.2	-2.8	±	0.1	2.4	±	0.1	0.6
I51 HD	dil	5.7	±	0.3	-2.9	±	0.1	1.9	±	0.1	
I51 HD	dex	7.5	±	0.3	-2.9	±	0.1	2.5	±	0.1	0.6
I51 HD	peg	6.9	±	0.3	-2.9	±	0.1	2.3	±	0.1	0.4
V52 HG	dil	5.2	±	0.2	-2.8	±	0.1	1.9	±	0.1	
V52 HG	dex	6.7	±	0.2	-2.8	±	0.1	2.4	±	0.1	0.6
V52 HG	peg	6.3	±	0.2	-2.8	±	0.1	2.3	±	0.1	0.4
A61 HB	dil	6.1	±	0.2	-2.9	±	0.1	2.1	±	0.1	
A61 HB	dex	7.9	±	0.2	-2.9	±	0.1	2.7	±	0.1	0.6
A61 HB	peg	7.2	±	0.2	-2.9	±	0.1	2.4	±	0.1	0.4
V63 HG1	dil	6.6	±	0.3	-2.9	±	0.1	2.3	±	0.1	
V63 HG1	dex	8.2	±	0.3	-2.9	±	0.1	2.9	±	0.1	0.6
V63 HG1	peg	7.5	±	0.3	-2.9	±	0.1	2.6	±	0.1	0.3
V63 HG2	dil	6.5	±	0.5	-2.3	±	0.2	2.8	±	0.3	
V63 HG2	dex	7.8	±	0.5	-2.3	±	0.2	3.4	±	0.3	0.6
V63 HG2	peg	8.0	±	0.5	-2.3	±	0.2	3.5	±	0.3	0.7
T64 HG	dil	5.1	±	0.3	-3.0	±	0.1	1.7	±	0.1	
T64 HG	dex	6.9	±	0.3	-3.0	±	0.1	2.3	±	0.1	0.6
T64 HG	peg	6.3	±	0.3	-3.0	±	0.1	2.1	±	0.1	0.4

Discussion of potential enthalpic and entropic contributions regarding crowding effects on the thermodynamic stability of proteins determined in presence of urea

Chemical unfolding of proteins as induced by urea occurs by a combined mechanism of direct interaction of urea molecules with the protein backbone and its side chains as well as by indirect changes in the hydrogen-bonding network of the hydration sphere of the protein [6]. Both of these effects favor the more expanded unfolded protein ensemble over the compact, folded conformation. Whereas chemical interactions are generally considered as enthalpic contributions, the steric expansion of the protein can be regarded as an entropic cost of the reaction.

In presence of crowding agents, potential enthalpic as well as entropic changes in the free energy of unfolding can be anticipated. On the one hand, entropic changes due to the steric hindrance of the protein's expansion in the course of the unfolding reaction can occur as a certain volume of the solution is not available for the protein anymore ("excluded volume effect"). On the other hand, enthalpic contributions due to direct stabilizing or destabilizing contact interactions of crowding molecules may arise with either the unfolded protein ensemble or the native state or both. It can be derived that for large polymeric crowders like PEG and dextran, entropic effects dominate over enthalpic effects in general [7], mitigated in some cases by enthalpic effects while summarizing reported studies on proteins which have been conducted in presence of crowding agents. Systematic investigations on potential enthalpic interactions of PEG could show that this crowding agent shows similarities to a weak organic solvent as it preferentially solubilizes in general not aliphatic side chains but aromatic side chains comprising tryptophan (Trp) as well as phenylalanine (Phe) [8]. Thus, for proteins bearing those side chains in the hydrophobic core of their native state, interactions with PEG could potentially favor the unfolded ensemble if the enthalpic gain of Trp/Phe side chain exposure energetically outweighs the enthalpically disfavored exposure of all aliphatic, polar and charged side chains as well as the entropic penalty of unfolding. Knowles et *al.* have shown that this scenario is the case for the unfolding reaction of the DNA-binding domain of lac repressor [9]. The enthalpic contribution due to preferential interaction of PEG with aromatic side chains could be quantified as a perturbation of the *m* value (cooperativity of unfolding) which corresponds to significant changes in the exposed surface area upon unfolding [9]. In the case of *Bs*CspB here, we found that the *m* value is not perturbed in the

presence of 120 g/L PEG1 compared to dilute solution (and also not for 120 g/L Dex20). The aromatic residues comprising *Bs*CspB are not contributing to the hydrophobic core of this protein but they are exposed both in the native state as well as in the unfolded ensemble.

To sum up, we cannot exclude potential enthalpic interactions of PEG1 with *Bs*CspB and corresponding aromatic side chains but we note that there is no change in *m* value detectable in presence of 120 g/L PEG1 which would be one prominent parameter of an existing enthalpic interaction. Note that we discuss other parameters for potential enthalpic interactions possibly existing between PEG1/Dex20 and *Bs*CspB in the main part of our manuscript.

Thus, the gain in free energy of unfolding leads to a significant increase in thermodynamic stability of *Bs*CspB upon addition of 120 g/L PEG1 or 120 g/L Dex20 which cannot be attributed to favorable aromatic interactions [8]. Further, the gain in thermodynamic stabilization seen for PEG1 is of same extent as in the presence of the macromolecular crowder Dex20 which equals the expected gain in stability due to excluded volume theory.

References

1 Pace, C. N. in *Methods in Enzymology* Vol. 131 266-280 (Academic Press, 1986).

2 Bolen, D. W. & Santoro, M. M. Unfolding free energy changes determined by the linear extrapolation method. 2. Incorporation of .DELTA.G.degree.N-U values in a thermodynamic cycle. *Biochemistry* **27**, 8069-8074, doi:10.1021/bi00421a015 (1988).

3 Santoro, M. M. & Bolen, D. W. Unfolding free energy changes determined by the linear extrapolation method. 1. Unfolding of phenylmethanesulfonyl alpha-chymotrypsin using different denaturants. *Biochemistry* **27**, 8063-8068, doi:10.1021/bi00421a014 (1988).

4 Greene, R. F. & Pace, C. N. Urea and Guanidine Hydrochloride Denaturation of Ribonuclease, Lysozyme, α-Chymotrypsin, and β-Lactoglobulin. *Journal of Biological Chemistry* **249**, 5388-5393 (1974).

5 Köhn, B. & Kovermann, M. Macromolecular Crowding Tunes Protein Stability by Manipulating Solvent Accessibility. *ChemBioChem* **20**, 759-763, doi:10.1002/cbic.201800679 (2019).

6 Bennion, B. J. & Daggett, V. The molecular basis for the chemical denaturation of proteins by urea. *Proc Natl Acad Sci USA* **100**, 5142, doi:10.1073/pnas.0930122100 (2003).

7 Sukenik, S., Sapir, L. & Harries, D. Balance of enthalpy and entropy in depletion forces. **18**, 495-501, doi:10.1016/j.cocis.2013.10.002 (2013).

8 Hirano, A., Shiraki, K. & Arakawa, T. Polyethylene glycol behaves like weak organic solvent. *Biopolymers* **97**, 117-122, doi:10.1002/bip.21708 (2012).

9 Knowles, D. B. *et al.* Chemical Interactions of Polyethylene Glycols (PEGs) and Glycerol with Protein Functional Groups: Applications to Effects of PEG and Glycerol on Protein Processes. *Biochemistry* **54**, 3528-3542, doi:10.1021/acs.biochem.5b00246 (2015).
