# Supplementary material for: All atom insights into the impact of crowded environments on protein stability by NMR spectroscopy
Source: Nat Commun. 2020 Nov 13;11:5760. doi: 10.1038/s41467-020-19616-w (PMC7666220; doi:10.1038/s41467-020-19616-w)
Supplement: Supplementary file 3 — Reporting Summary [file 41467_2020_19616_MOESM3_ESM.pdf]

nature research

Corresponding author(s): Michael Kovermann

Last updated by author(s): Oct 12, 2020

Reporting Summary

Nature Research wishes to improve the reproducibility of the work that we publish. This form provides structure for consistency and transparency in reporting. For further information on Nature Research policies, see our Editorial Policies and the Editorial Policy Checklist.

Statistics

For all statistical analyses, confirm that the following items are present in the figure legend, table legend, main text, or Methods section.

n/a	Confirmed	
☒	☐	The exact sample size (*n*) for each experimental group/condition, given as a discrete number and unit of measurement
☐	☒	A statement on whether measurements were taken from distinct samples or whether the same sample was measured repeatedly
☒	☐	The statistical test(s) used AND whether they are one- or two-sided<br>*Only common tests should be described solely by name; describe more complex techniques in the Methods section.*
☒	☐	A description of all covariates tested
☒	☐	A description of any assumptions or corrections, such as tests of normality and adjustment for multiple comparisons
☐	☒	A full description of the statistical parameters including central tendency (e.g. means) or other basic estimates (e.g. regression coefficient) AND variation (e.g. standard deviation) or associated estimates of uncertainty (e.g. confidence intervals)
☒	☐	For null hypothesis testing, the test statistic (e.g. F, t, r) with confidence intervals, effect sizes, degrees of freedom and P value noted<br>*Give P values as exact values whenever suitable.*
☒	☐	For Bayesian analysis, information on the choice of priors and Markov chain Monte Carlo settings
☒	☐	For hierarchical and complex designs, identification of the appropriate level for tests and full reporting of outcomes
☒	☐	Estimates of effect sizes (e.g. Cohen's *d*, Pearson's *r*), indicating how they were calculated

Our web collection on statistics for biologists contains articles on many of the points above.

Software and code

Policy information about availability of computer code

Data collection	TopSpin 3.2.5 (Bruker BioSpin)
Data analysis	TopSpin 3.2.5: Used for analysis of integrals of 1D 1H data; Igor 6.37 (Wavemetrics): Used for determination of diffusion coefficient; Origin 2019b (OriginLab): Plotting of experimental data and analysis of thermodynamic parameters; NMRPipe 64-bit Mac OS X version: processing 2D NMR data; NMRView 8.0.a27: Plotting 2D NMR data including data analysis by obtaining height of cross-peaks; ProtScale (Bioinformatics Resource Portal ExPASy, https://web.expasy.org/protscale): Determination of hydropathy score; Mestrenova 14 (Mestrelab Research): baseline correction and integration of one-dimensional proton NMR data used for diffusion analysis

For manuscripts utilizing custom algorithms or software that are central to the research but not yet described in published literature, software must be made available to editors and reviewers. We strongly encourage code deposition in a community repository (e.g. GitHub). See the Nature Research guidelines for submitting code & software for further information.

Data

Policy information about availability of data

All manuscripts must include a data availability statement. This statement should provide the following information, where applicable:

- Accession codes, unique identifiers, or web links for publicly available datasets
- A list of figures that have associated raw data
- A description of any restrictions on data availability

All data are available on reasonable request from the corresponding author.

Field-specific reporting

Please select the one below that is the best fit for your research. If you are not sure, read the appropriate sections before making your selection.

[x] Life sciences   [ ] Behavioural & social sciences   [ ] Ecological, evolutionary & environmental sciences

For a reference copy of the document with all sections, see nature.com/documents/nr-reporting-summary-flat.pdf

Life sciences study design

All studies must disclose on these points even when the disclosure is negative.

Sample size	The study presented in this manuscript does not comprise sample sizes. All molecules present in the NMR sample tube have been collectively probed. The analysis of folding-to-unfolding transitions observed in this study has been comprehensively done. No data has been excluded from data analysis. Thus the analysis of folding-to-unfolding transitions of amino acids comprising the protein under study has been done without any bias (see below).
Data exclusions	No experimental data were excluded from data analysis.
Replication	The design of the experimental setup was done in such a way that each protein sample which has been used for monitoring folding-to-unfolding transitions has been probed 30 times in total (permitting different concentrations of urea). For diffusion measurements, three different gradient strengths have been repeated three times each. Both protein samples used for diffusion measurements have been probed 30 times in total covering low to high numbers of magnetic field gradients. The replication of acquired data was successful.
Randomization	The experimental data acquired for this manuscript have been obtained by high-resolution NMR spectroscopy. The number of protein molecules probed by this experimental technique is in the order of 10^17 based on the concentration used. Thus no randomization can be inherently conducted.
Blinding	The experiments conducted in this study were not blinded.

Reporting for specific materials, systems and methods

We require information from authors about some types of materials, experimental systems and methods used in many studies. Here, indicate whether each material, system or method listed is relevant to your study. If you are not sure if a list item applies to your research, read the appropriate section before selecting a response.

Materials & experimental systems

n/a	Involved in the study
[x]	[ ] Antibodies
[x]	[ ] Eukaryotic cell lines
[x]	[ ] Palaeontology and archaeology
[x]	[ ] Animals and other organisms
[x]	[ ] Human research participants
[x]	[ ] Clinical data
[x]	[ ] Dual use research of concern

Methods

n/a	Involved in the study
[x]	[ ] ChIP-seq
[x]	[ ] Flow cytometry
[x]	[ ] MRI-based neuroimaging
