# Supplementary material for: All atom insights into the impact of crowded environments on protein stability by NMR spectroscopy
Source: Nat Commun. 2020 Nov 13;11:5760. doi: 10.1038/s41467-020-19616-w (PMC7666220; doi:10.1038/s41467-020-19616-w)
Supplement: Supplementary file 2 — Peer Review [file 41467_2020_19616_MOESM2_ESM.pdf]

Reviewers' comments:

Reviewer #1 (Remarks to the Author):

The authors have evaluated the impact of macromolecular crowding (MC) on protein (cold shock protein) stability at an atomic level with multidimensional NMR. The authors have compared the impact of MC on the ensemble of unfolded conformations at chemically denaturing conditions with those of folded conformations. The results have shown that the impacts are greater in the former than the latter. It would be great if the authors can present these data on a protein structure in the main text by moving Fig S7 there. Since the authors claimed their results are at atomistic levels in terms of side chain or backbones, figure S7 should show the impact of MC atomistically, instead of ribbon structures. It is not clear why Dex 20 and PEG1 were chosen for this investigation. The work of crowding on folding stability by the Schuler group was based on a systematic investigation with crowders in a wide range of sizes and types. As the authors wrote on the manuscript, the MC can be crowder-dependent. Authors should state clearly about why only these two crowders are chosen and why they think these results can be generalized to other studies.

Reviewer #2 (Remarks to the Author):

Manuscript titled “All atom insights into the impact of macromolecular crowding on protein stability by NMR spectroscopy” by Birgit Kohn and Michael Kovermann describes an attempt to measure protein stability in different crowding conditions at atomic level by means of NMR spectroscopy.

In contrast to studies in vivo, that according to the authors lack generality, they choose a systematic in vitro approach that they baptize as a bottom-up approach.

Protein unfolding in this study is induced by addition of urea, a well-known destabilizing agent. Stability was gauged by means of 1D and 2D NMR spectroscopy.

The amount of data presented and their quality is really impressive. The authors are very thorough in designing sophisticated NMR experiments and analyzing the data.

One major reservation is that using chemical destabilization (instead of thermal destabilization) makes it very difficult to discriminate between entropic effects and chemical interactions.
The authors chose chemical unfolding to judge the possible entropic effect of volume exclusion in crowding conditions. This choice is intrinsically wrong. The main effects that they are monitoring are probably chemical interactions, a situation too far from physiological conditions. To make things worse they use, as a macromolecular crowder, a 1 kDa PEG. This is hardly a macromolecule. Suffice it to say that it is almost one order of magnitude smaller than the protein under study whose molecular weight is over 7 kDa.

Last but not least it appears that they assume that any residue can report correct unfolding.
They were possibly inspired by ref. #25 in which unfolding in cell was monitored by using, as the only residue, the C terminal one (!). This choice was not even wrong.

A minor point: Supplementary Figure 1A is absent, I can only see Figures 1B and 1C.

Reviewer #3 (Remarks to the Author):

While these results are potentially interesting I personally don't find them all too convincing.

I'm not sure how they follow the disappearance of the folded state from 1D spectra, especially in the

aliphatic region where there is considerable overlap between folded and unfolded spectra. I'm also not sure how they following things from 2D spectra. For example, are they looking last changes in peak volume or peak height. This is important because extensive broadening owing to exchange processes can occur especially near the mid-point.

I think they need to show the 2D spectra as a function of urea concentration in the supplementary and indicate exactly what they are looking at.

In addition the manuscript is excessively long for the content and difficult to follow. Moreover, the conclusion section is loaded with speculation for which there is no evidence.

I think the data are probably solid but the paper needs extensive revision and a rewrite prior to acceptance.

Reviewers' comments

Reviewer #1

The authors have evaluated the impact of macromolecular crowding (MC) on protein (cold shock protein) stability at an atomic level with multidimensional NMR. The authors have compared the impact of MC on the ensemble of unfolded conformations at chemically denaturing conditions with those of folded conformations. The results have shown that the impacts are greater in the former than the latter. It would be great if the authors can present these data on a protein structure in the main text by moving Fig S7 there. Since the authors claimed their results are at atomistic levels in terms of side chain or backbones, figure S7 should show the impact of MC atomistically, instead of ribbon structures.

> *Response:* The reviewer is right that we have aimed to expand the impact of MC on the thermodynamic stability of a protein to an atomic scale taking both backbone and side chain atoms into account. Figure S7 highlights amino acids that possess values of C_M at a residue-by-residue level (individual midpoint of the folding-to-unfolding transition) which do not lie within the range covered by the mean and one standard deviation regarding all values of C_M acquired at dilute, 120 g/L Dex20 or 120 g/L PEG1 conditions.
> We recognize the valuable suggestion of the reviewer to present our results of the observed general increase in thermodynamic stability on the three-dimensional structure of *Bs*CspB in the main part of our manuscript. To do so, we have prepared the new Figure 5 showing the increase in thermodynamic stability of *Bs*CspB which has been obtained for NH signals when 120 g/L PEG1 or 120 g/L Dex20 are present. Thereby we visualize that the presence of either 120 g/L PEG1 or 120 g/L Dex20 affects ΔC_M comparably (as presented and discussed in our manuscript). This Figure 5 converts the numerical values presented in Supplementary Table 2 into a representation which is easier to read. We are thankful to the reviewer for this comment.
> Consequently, we present now *all* numerical values shown in Supplementary Table 3 (data obtained for CH signals), Supplementary Table 4 (data obtained for CH_2 signals) and Supplementary Table 5 (data obtained for CH_3 signals) on the three-dimensional structure of *Bs*CspB, too. Thus, we have prepared a new Supplementary Figure 17 which highlights ΔC_M values obtained for individual CH, CH_2 and CH_3 signals when 120 g/L PEG1 or 120 g/L Dex20 are present. This series of images explicitly highlights the impact of MC on the thermodynamic stability of *Bs*CspB which has been unraveled in our study at an atomic scale.

It is not clear why Dex 20 and PEG1 were chosen for this investigation. The work of crowding on folding stability by the Schuler group was based on a systematic investigation with crowders in a wide range of sizes and types. As the authors wrote on the manuscript, the MC can be crowder-dependent. Authors should state clearly about why only these two crowders are chosen and why they think these results can be generalized to other studies.

Response: We have made a strategic choice of the MC agents to be used in our study. Firstly, MC agents have been chosen in the way that they are smaller or larger (regarding molecular weight, M_W) as the protein under investigation. This is the case here as *Bs*CspB possesses M_W= 7.3 kDa compared to M_W = 1 kDa (PEG1) and M_W = 20 kDa (Dex20) enabling to contribute to the discussion of the potential dependence the M_W of MC agents has on the thermodynamic stability of the protein under study. Secondly, we have looked for MC agents which differ in intrinsic parameters like e.g. polarity. This is the case here, too, as Dex20 possesses a higher polarity compared to PEG1. Thirdly, we have looked for inert MC agents which do not directly interact neither to the protein nor to urea as the denaturant used in our study. Indeed, we have found no experimental evidence that PEG1 and Dex20 interact with urea (see new Supplementary Figure 15 and detailed discussion with Reviewer #2). In contrast, protein crowders would directly interact with urea changing their conformation during the unfolding process of *Bs*CspB, too. Please note additionally that we have recently shown that both PEG and Dex do not directly interact with *Bs*CspB by using high-resolution NMR spectroscopy (Köhn & Kovermann, ChemBioChem, 2019).

Accordingly, we have added three sentences on page 4 into our manuscript:
"This strategic choice is based on a recent finding obtained by applying a combination of fluorescence, circular dichroism and NMR spectroscopy showing that PEG and Dex20 molecules have a pronounced effect on the overall thermodynamic stability of *Bs*CspB (ref. 23). It could be shown that the presence of equally concentrated solutions containing PEG1, PEG8, PEG35 or Dex20 molecules impacts the overall thermodynamic stability of *Bs*CspB quantitatively in the same manner regardless of differences in molecular weight and polarity. Selecting PEG1 and Dex20 as done here enables to perform highly-resolved NMR spectroscopic experiments on a protein which is significantly larger than the crowding molecules regarding PEG1 on the one hand or smaller regarding Dex20 on the other hand."

We are aware about the important work regarding macromolecular crowding the Schuler group has performed. Consequently, we highlight one manuscript presenting experimental results which they have obtained by using a wide set of MC agents on four different IDPs (intrinsically disordered proteins) in the

revised version of our manuscript (ref. 24). In this respect we would like to emphasize that even though our study applied two different MC agents "only", we have been able to obtain experimental data for a huge set of probes comprising the protein under study (in the order of hundreds) in comparison to studies which rely on a single probe per experimental condition which has been used. We mention that Reviewer #2 states the amount of data presented in our manuscript as "really impressive" (see below). In addition, we have shown before that PEG1 impacts the overall thermodynamic stability of *Bs*CspB quantitatively in the same manner as PEG8 and PEG35 by acquiring and analyzing experimental data using fluorescence and circular dichroism spectroscopy (Köhn & Kovermann, ChemBioChem, 2019).

Why do we think we can generalize our results to other studies? We have chosen *Bs*CspB as a protein which represents a general two state folding reaction process that is simple and encountered for many proteins which have been studied in literature. Moreover, *Bs*CspB possesses a beta barrel fold which is a common, wide spread protein fold that many proteins possess. Thus, we believe that a generalization of the results we have obtained here to proteins showing folding characteristics which are comparable to *Bs*CspB is feasible. We are convinced that a further generalization to proteins possessing a different fold than beta barrel or exhibiting a more complex folding model than two-state is possible, too. This would need further investigations directed in the same manner as we have conducted here enabling a reliable conclusion regarding a possible cooperative increase in overall thermodynamic stability due to the presence of MC agents.

Accordingly, we have added the following sentence at the end of the Discussion section on page 16 into our manuscript:
"Finally, we believe that due to the systematic experimental setup and comprehensive data analysis applied here a generalization of the results to proteins showing folding characteristics which are comparable to *Bs*CspB is feasible."

Reviewer #2

Manuscript titled "All atom insights into the impact of macromolecular crowding on protein stability by NMR spectroscopy" by Birgit Kohn and Michael Kovermann describes an attempt to measure protein stability in different crowding conditions at atomic level by means of NMR spectroscopy. In contrast to studies in vivo, that according to the authors lack generality, they choose a systematic in vitro approach that they baptize as a bottom-up approach. Protein unfolding in this study is induced by addition of urea, a well-known destabilizing agent. Stability was gauged by means of 1D and 2D NMR spectroscopy. The amount of data presented and their quality is

really impressive. The authors are very thorough in designing sophisticated NMR experiments and analyzing the data.

> *Response:* We are very happy to read that the reviewer states the amount and quality of data presented in our manuscript “really impressive”. This claim has been the major driving force in the design of our study.

One major reservation is that using chemical destabilization (instead of thermal destabilization) makes it very difficult to discriminate between entropic effects and chemical interactions. The authors chose chemical unfolding to judge the possible entropic effect of volume exclusion in crowding conditions. This choice is intrinsically wrong. The main effects that they are monitoring are probably chemical interactions, a situation too far from physiological conditions.

> *Response:* We have used chemical denaturation instead of thermal denaturation here by considering three advantages of chemical compared to thermal denaturation of proteins:
>
> - Chemical denaturation as used here leads to a fully reversible folding-to-unfolding reaction of *Bs*CspB enabling a highly precise quantitative thermodynamic analysis of data. Note that the acquisition of NMR data representing 30 different urea concentrations has been achieved by mutual mixing of two NMR tubes containing the same amount of protein but differing in urea concentration (specifications at start using e.g. dilute conditions: $c^{\text{urea, tube1}}$ = 0 M, $c^{\text{urea, tube2}}$ = 6.05 M). Finally, after conducting 14 mixing steps), both tubes contain a comparable concentration of urea. Focusing on this last mixing step, the fraction of native protein, f_{n}, is comparable in both tubes underlying the full reversibility of the folding-to-unfolding reaction of *Bs*CspB during the entire course of the experiment.
> - Additionally, applying chemical instead of thermal denaturation enables to monitor the folding-to-unfolding reaction by using amide protons. Note that amide protons tend to increasingly exchange with water protons when the temperature is raised. Thus, applying chemical denaturation expands NMR detection from CH to NH correlations significantly avoiding loss of valuable spectral information.
> - Moreover, please note that the diffusion analysis presented in our manuscript utilizes resonance signals comprising aromatic protons. Consequently, our study makes full use in observing and analyzing the entire repertoire of protons that proteins inherently offer by applying chemical denaturation: (i) amide protons, (ii) aliphatic protons and (iii) aromatic protons.
>
> The analysis of thermally denatured *Bs*CspB has been done before by us by applying one-dimensional proton NMR spectroscopy (Köhn & Kovermann,

ChemBioChem, 2019) preventing atomic resolution. In this published study, the application of MC agents PEG1, PEG8, PEG35 and Dex20 leads to changes in ΔG^0 values of *Bs*CspB which are comparable among each other. These results strongly support the findings of the present study.

Now we respond to the discussion of "chemical interactions". First, we have entirely worked with synthetic MC agents which are supposed to be inert even in the presence of urea. To experimentally confirm such inertness of PEG1 and Dex20 in a protein-urea solution, we have performed several control experiments. In this respect, Figure 4B in the original submission presents chemical shift perturbations, CSPs, of NH cross signals comprising *Bs*CspB using two-dimensional ^{1}H-^{15}N HSQC spectra comparing c^{urea} = 0 M with c^{urea} = 2.97 M under dilute conditions and CSP values obtained when 120 g/L Dex20 or 120 g/L PEG1 are present (concentrations of urea are similar). The high reproducibility among the three conditions suggests that both Dex20 and PEG1 do not disturb the impact of urea on *Bs*CspB. We realize the importance of this discussion and have prepared the new Supplementary Figure 14 correlating CSP values obtained in presence of MC agents with CSP values obtained in absence of MC agents. These two plots visualize a strong correlation of CSP values obtained at different conditions displaying no experimental evidence that PEG1 or Dex20 modify the impact urea has on *Bs*CspB. Thus, we can conclude that the chemical environment of NH cross signals comprising *Bs*CspB is not changed when 120 g/L PEG1 or 120 g/L Dex20 are present using the applied conditions.
Moreover, we have now investigated a potential chemical interaction between PEG1 or Dex20 with urea. Thus, one-dimensional proton NMR spectra acquired for free PEG1 and PEG1 + c^{urea} = 6 M (new Supplementary Figure 15 A) as well as for free Dex20 and Dex20 + c^{urea} = 6 M (new Supplementary Figure 15 B) are compared. In both cases, no significant changes in chemical shifts, line widths or signal intensities have been observed indicating inertness of PEG1 and Dex20 regarding urea.

Accordingly, we have changed the section "Chemical shift perturbations in *Bs*CspB induced by urea under dilute and MC conditions" to "Analyzing potential interaction of urea with MC agents and with *Bs*CspB in presence of MC" on page 10 in the revised version and have added the following sentences on page 11 into our manuscript:
"Furthermore, one-dimensional proton NMR spectroscopy has been applied to monitor potential impact of urea on MC agents used in this study. Neither perturbations in chemical shifts nor modifications in line shape of resonance signals comprising PEG1 or Dex20 could be observed comparing c^{urea} = 0 M with c^{urea} = 6 M (Supplementary Fig. 15). This result indicates an inertness of PEG1 and Dex20 regarding urea even if present at a high concentration."

Why is the choice of urea as chemical denaturant sound in order to probe effects of MC on entropy? We cannot exclude that unspecific interactions among MC agents and *Bs*CspB may occur, but, if any present, they are too subtle to impact chemical shifts of resonance signals observed by NMR spectroscopy significantly. In fact, the chemical environment of resonance signals comprising *Bs*CspB applying MC does not differ from that observed for dilute conditions. Thus, we neither observe an enhancement of specific nor of unspecific chemical interactions in our experimental setup which already enables detection of protein signals at atomic resolution. We conclude that the chemical interaction of urea with *Bs*CspB is unchanged comparing absence and presence of MC agents as no interfering chemical interactions between the protein and the MC agent as well as urea and MC agent can be observed.

Applying a concentration of c = 120 g/L of MC agents PEG1 and Dex20 - which significantly differ in size and polarity - underline that the entropic effect of volume exclusion is indeed a valid explanation for the here observed increase in thermodynamic stability of *Bs*CspB. This observation is supported by the wealth of experimental data acquired in the present study.

To make things worse they use, as a macromolecular crowder, a 1 kDa PEG. This is hardly a macromolecule. Suffice it to say that it is almost one order of magnitude smaller than the protein under study whose molecular weight is over 7 kDa.

Response: We agree with the reviewer that PEG1 possesses a molecular weight, M_W, which is significantly smaller than the M_W of *Bs*CspB. We note that we have carefully designed our study in such a way that we have chosen MC agents which are highly diverse according to their inherent parameters like size, polarity, shape (see also response to Reviewer #1). Most importantly, we have shown before that PEG1 impacts the overall thermodynamic stability of *Bs*CspB quantitatively in the same manner as PEG8 and PEG35 (Köhn & Kovermann, ChemBioChem, 2019). In fact, monitoring folding-to-unfolding transitions of *Bs*CspB induced by an increasing concentration of urea by applying fluorescence and circular dichroism spectroscopy has shown that the presence of PEG1 increases ΔG^0 values of *Bs*CspB just as PEG8. Advantageously, the solubility and miscibility of PEG1 in combination with c^{urea} = 6 M is clearly superior to PEG8. This is an important issue for the high-resolution NMR experiments conducted here which rely on an accurate mixing of solutions present in tiny NMR tubes.

For the sake of completeness, using Dex20 as the second MC agent applied here enables us to draw conclusions about the impact of MC on the overall thermodynamic stability of *Bs*CspB in a highly universal manner. Dex20 possesses a higher polarity than PEG1 and is more than one order of magnitude larger than PEG1 depicting about three times the size of *Bs*CspB.

Last but not least it appears that they assume that any residue can report correct unfolding. They were possibly inspired by ref. #25 in which unfolding in cell was monitored by using, as the only residue, the C terminal one (!). This choice was not even wrong.

> *Response:* We agree with the reviewer that not every residue present in a folded protein can inherently report on the correct unfolding process of the entire protein. In our study, we do not claim that any NMR resonance signal can report on global thermodynamic stability of *Bs*CspB. In fact, we compare the loss of native structure dependent on the concentration of urea which has been monitored at every atom individually. In doing so we have monitored a cooperative increase in overall thermodynamic stability of *Bs*CspB induced by MC analysing all atoms individually providing a complete, atomic analysis. The analysis of individual folding-to-unfolding transitions was not aimed for monitoring global protein stability in these sites but rather illuminating potential local differences which may be evoked by the presence of MC agents.
> Thus, we absolutely agree with the reviewer that monitoring a urea dependent folding-to-unfolding transition of a single residue only may not reflect the thermodynamic stability of the entire protein. Therefore, we thoroughly investigated all available resonance signals comprising *Bs*CspB instead of focussing on only one experimental probe. Furthermore, we have not compared individual folding-to-unfolding transitions with the overall thermodynamic stability of *Bs*CspB but analysed changes in thermodynamic stability of individual atoms comparing dilute with MC conditions.
> For the comparison with "globally" determined thermodynamic stability of *Bs*CspB (as published in Köhn & Kovermann, ChemBioChem, 2019) we always refer to a mean and standard deviation taking all individual residues into account. We do not claim to monitor global protein stability by monitoring one single residue.

A minor point: Supplementary Figure 1A is absent, I can only see Figures 1B and 1C.

> *Response:* To the best of our knowledge, Supplementary Figure 1A (displaying one-dimensional proton NMR spectra of native (colored in black) and the unfolded protein ensemble of *Bs*CspB (colored in red) is present. We acknowledge that this Figure differs in dimension compared to the two two-dimensional NMR spectra shown in Supplementary Figure 1B, C.

Reviewer #3

While these results are potentially interesting I personally don't find them all too convincing. I'm not sure how they follow the disappearance of the folded state from 1D

spectra, especially in the aliphatic region where there is considerable overlap between folded and unfolded spectra.

> *Response:* Primarily, we have acquired one-dimensional proton NMR spectra to probe the overall thermodynamic stability of *Bs*CspB globally obtaining C_M values following chemically induced folding-to-unfolding transitions. No assignment of a substantial amount of individual residues can be made at this point. Accordingly, two-dimensional NMR spectra (^{1}H-^{15}N as well as ^{1}H-^{13}C HSQCs) have been acquired and analyzed subsequently.
> Concerning the type of analysis of one-dimensional proton NMR spectra: We now directly refer to ref. 23 in the Material & Methods part of our manuscript and, additionally, present seven one-dimensional NMR spectra of *Bs*CspB acquired in the range of 0 M ≤ c^{urea} ≤ 6.1 M in the Supplementary Information (new Supplementary Figure 2).

I'm also not sure how they following things from 2D spectra. For example, are they looking last changes in peak volume or peak height. This is important because extensive broadening owing to exchange processes can occur especially near the mid-point.

> *Response:* The reviewer is right, it is important to differentiate between peak volume and peak height. We have put a lot of efforts into the choice between peak height and peak volume. One rationale to work with peak height is based on the fact that cross peaks which are spectrally at close quarters cannot be reliably analyzed when peak volume is used due to adjacent boxes applied for determining the particular size of peak volumes.
> Being quantitatively, we have analyzed 13 cross signals which are spectrally well separated at all experimental conditions in two-dimensional ^{1}H-^{15}N HSQC NMR spectra of *Bs*CspB using both peak height and peak volume. We present this analysis as new Supplementary Figure 8. As a result, the overall increase in thermodynamic stability, ΔC_M, determined for these residues using peak height is comparable with ΔC_M values determined using peak volume. Thus, we conclude that using peak height for the analysis of the experimental data acquired in our study is permitted. This strategy enabled us to analyse folding-to-unfolding transitions of 53 (instead of 13) amide proton cross signals.
>
> Accordingly, we have added two sentences on page 8 into our manuscript:
> "In addition, another control has shown that using signal intensity or signal volume of cross signals used for the analysis of individual folding-to-unfolding transitions leads to similar results in ΔC_M (Supplementary Fig. 8). Therefore, all cross signals acquired in two-dimensional heteronuclear NMR spectra in this study have been analyzed using signal intensity enabling a reliable readout of data even if cross signals are in closely spectral proximity."

I think they need to show the 2D spectra as a function of urea concentration in the supplementary and indicate exactly what they are looking at.

Response: We are thankful to the Reviewer bringing up the idea of presenting the 2D spectra dependent on the concentration of urea. Now, we have included two new Supplementary Figures 3 and 12 into the revised version of our manuscript. Supplementary Figure 3 shows a series of two-dimensional ^{1}H-^{15}N HSQC spectra dependent on the concentration of urea whereas Supplementary Figure 12 presents a series of ^{1}H-^{13}C HSQC spectra dependent on the concentration of urea.

In addition, the manuscript is excessively long for the content and difficult to follow. Moreover, the conclusion section is loaded with speculation for which there is no evidence. I think the data are probably solid but the paper needs extensive revision and a rewrite prior to acceptance.

Response: We have worked hard to present a comprehensive set of experimental data in an appealing manner. We are aware that the manuscript comprises - for this reason - a wealth of spectral information. Thus, we present a new Figure 5 highlighting the cooperative increase of thermodynamic stability of *Bs*CspB caused by the addition of MC agents at a residue-by-residue level in an illustrative manner. This increase in thermodynamic stability of *Bs*CspB has been additionally highlighted by the new Supplementary Figure 17 visualizing carbon bound protons at an atomic scale in an even more delicate manner. Moreover, presentation of individual one-dimensional proton (new Supplementary Figure 2) as well as two-dimensional heteronuclear NMR spectra (new Supplementary Figures 3 and 12) improves – in our opinion - the readability of our manuscript significantly.
Concerning the conclusion section: We have put a lot of efforts to classify the results obtained in the present study with observations reported before related to the effect of MC in a highly systematic manner. Thus, we structured the discussion into four separated parts while citing the most relevant literature.

We are convinced that our revised manuscript profited significantly by including one new main Figure, seven new Supplementary Figures and editing the text presented.

Figure 5 has nicely summarized the results and successfully communicated the importance of this work.

Reviewer #2 (Remarks to the Author):

Revised version of manuscript titled “All atom insights into the impact of macromolecular crowding on protein stability by NMR spectroscopy” by Birgit Kohn and Michael Kovermann.

Answers from authors:

The answer to the objection on using chemical denaturation instead of thermal denaturation is in the style of their manuscript, i.e. very detailed and accurate but not to the point.
Essentially, the authors fail to give a clear answer on the intrinsic difficulty of using a mixture of chemicals to study unfolding and particularly the influence of purely entropic factors, possibly excluding ALL enthalpic factors.

The use of a low molecular weight PEG as a macromolecular crowder is oxymoronic. This kind of crowder behaves as an organic solvent.

In conclusion, I still think that the work is extensive and well conducted BUT the analysis of the data is not consistent with the goal.
The data cannot be interpreted as macromolecular crowding.

The study is valuable and ought to be published, but the interpretation of the results should be changed.

Reviewer #3 (Remarks to the Author):

I believe the authors have satisfactorily addressed the comments of the 3 reviewers.

However, I would recommend some minor changes in nomenclature prior to publication:

(a) Throughout the MS the authors refer to cross signals. For example NH cross signals, CH cross signals, etc..... Cross signals is NOT a term used in the NMR literature. The authors are actually referring to "cross-peaks". And when they are talking for example about "NH cross signals", what they are really talking about are "1H-15N cross-peaks". It's important to get this right, not because one can't figure out what the authors are talking about, but rather because their notation gives the impression that they don't know anything about NMR.

(b) The authors are confused in terms of equating peak height (either the height of a resonance in a 1D spectrum or that of a cross-peak in a 2D correlation spectrum) with intensity. This is incorrect. Intensity refers to the area under a one-dimensional signal and the volume of a cross-peak in a 2D (or higher dimensional) spectrum. Recall intensity in NMR refers to magnetization, and magnetization relates to the integral under a resonance or cross-peak, NOT to peak height which is dictated by the transverse relaxation time(s). So keep it simple and refer explicitly to cross-peak (2D) height and cross-peak volumes/intensity. (And similar for the 1D case: signal height versus area under the signal which really can't be determined from 1D in the presence of any sort of significant overlap).

Reviewers' comments

Reviewer #1

Figure 5 has nicely summarized the results and successfully communicated the importance of this work.

> *Response:* We are very happy to read that the reviewer appreciates the work which we present in our manuscript.

Reviewer #2

Revised version of manuscript titled "All atom insights into the impact of macromolecular crowding on protein stability by NMR spectroscopy" by Birgit Kohn and Michael Kovermann.

Answers from authors: The answer to the objection on using chemical denaturation instead of thermal denaturation is in the style of their manuscript, i.e. very detailed and accurate but not to the point. Essentially, the authors fail to give a clear answer on the intrinsic difficulty of using a mixture of chemicals to study unfolding and particularly the influence of purely entropic factors, possibly excluding ALL enthalpic factors.

> *Response:* We fully agree with the reviewer that a careful evaluation of both entropic and enthalpic factors contributing to the observed increase in thermodynamic stability of *Bs*CspB is essential while monitoring chemical denaturation. Thus, the discussion section of our manuscript carefully addresses this issue.
>
> In detail, on page 13 of our manuscript we state "Thus, it has been discussed that **enthalpic contributions may add on to or counteract the entropic stabilization of the native state** [27,47,49,50]". Going along these lines, we explicitly state that "the effects reported for PEG are highly diverse" in this context (found also on page 13 of our manuscript) which is followed by a precise description of various observations reported in the literature (citations 3, 46, 51-56). Moreover, we state that "there have been attempts to classify proteins by their hydrophobicity or primary sequence in order to predict **negative or positive enthalpic contributions based on chemical interactions with the protein** [43,57] (found on page 13). We discuss potential **non-negligible enthalpic components reported for Dex20** [42] regarding the hydrogen bonding network and in context of the polarity of the crowding agents Dex20 and PEG1 used here (found on page 15). Consequently, we ask the following question to emphasize the important issue of potential enthalpic contributions regarding the observed increase in stability of *Bs*CspB (found on pages 15/16): "However,

what holds as a reason that **an enthalpic contribution (if existent) for *Bs*CspB can only be of negligible or equal extent for PEG1 and Dex20 as no hydrophobic interactions can be detected** whereas a couple of different proteins have shown such a contribution [67]?" We speculate that this feature is potentially based on the low hydrophobicity *Bs*CspB owns and its polyanionic net charge, estimated as -5.8 at pH 7 (found on page 16).

All in all, we explicitly address in our manuscript the importance of potential enthalpic contributions regarding the understanding of protein stability in a crowded environment. Please note that we put the results presented and discussed in our manuscript in the context of investigations performed on *Bs*CspB. We do not claim that potential enthalpic contributions do not exist while analyzing protein parameters in a crowded environment.

We have now also added one separated paragraph to the Supplementary Information acknowledging the importance of enthalpic and entropic contributions regarding crowding effects on the thermodynamic stability of proteins. This paragraph is entitled "Discussion of potential enthalpic and entropic contributions regarding crowding effects on the thermodynamic stability of proteins determined in presence of urea".

We would like to state that we have been careful regarding the use of urea as chemical denaturant in our study applying crowded environments. We have undertaken a series of highly-resolved experiments to evaluate a potential impact of urea on crowding agents. As a result, experimental data shown in Figure 4B, Supplementary Figure 14A and B, Supplementary Figure 15A and B give no evidence that a concentration of 120 g/L of crowding agents impacts urea.

Additionally, applying chemical instead of thermal denaturation enables to monitor the folding-to-unfolding reaction of proteins by using amide protons, too. Note that amide protons tend to increasingly exchange with water protons when the temperature is raised. Thus, applying chemical denaturation expands NMR detection from CH to NH correlations significantly avoiding loss of valuable spectral information.

The use of a low molecular weight PEG as a macromolecular crowder is oxymoronic. This kind of crowder behaves as an organic solvent.

Response: We thank the reviewer for this comment. We recognize that we have not been as precise as possible while referring to PEG1 in our manuscript. Thus, we modified the term *macromolecular crowder* in our manuscript whenever we refer to PEG1. E.g., we adapted the term "under dilute and **MC conditions**" to

"under dilute conditions and in **crowded environments**" appearing in three subheadings to emphasize the use of PEG1 possessing a relatively low molecular weight. Moreover, we adapted the last but one part of the discussion section of the manuscript summarizing the key findings of our study to (on page 16):

"Interestingly, the pronounced difference in molecular weight of crowding agents used here does not change the magnitude of the stabilization seen for *Bs*CspB. This demonstrates that a **crowded environment** which is composed of **molecules possessing a molecular weight of about 1 kDa only** procures an excluded volume effect regarding a protein of about 7 kDa in molecular weight."

In our opinion, this wording fully considers the molecular dimension of PEG1 and its impact on the thermodynamic stability of *Bs*CspB as probed at atomic resolution in our study. The observed increase in thermodynamic stability of *Bs*CspB as induced by either PEG1 or Dex20 is comparable. This holds for all sites which have been probed in our study.

Note, that we now separately outline the property of PEG1 potentially acting as organic solvent in the new paragraph "Discussion of potential enthalpic and entropic contributions regarding crowding effects on the thermodynamic stability of proteins determined in presence of urea" found in the Supplementary Information.

In conclusion, I still think that the work is extensive and well conducted BUT the analysis of the data is not consistent with the goal. The data cannot be interpreted as macromolecular crowding.

Response: We acknowledge that the two crowding agents which have been used in our study significantly differ in size and chemical properties. We fully agree with the reviewer that PEG1 does not possess a dimension which is generally used to term a molecule "macromolecule" - especially in comparison to the molecular dimension of Dex20. But even though molecular dimensions of these two crowding agents strongly differ (beside their differences in chemical properties) they lead to comparable results regarding protein stability which have been consistently obtained at atomic resolution in our study.

As outlined above, we have adapted our manuscript to consider the molecular dimension of PEG1. Thus, the wording "macromolecular crowding" has been changed e.g. to "crowded environment" or "crowding effects" whenever we refer to PEG1 in our manuscript.

Importantly, **we have adapted the title of our manuscript** to "All atom insights into the impact of crowded environments on protein stability by NMR spectroscopy" to account for the relatively low molecular weight of PEG1.

The study is valuable and ought to be published, but the interpretation of the results should be changed.

> *Response:* We are very happy to read that the reviewer supports the publication of our manuscript. We have changed the notation of PEG1 and have aimed to profoundly address potential enthalpic contributions arising from a crowded environment as outlined in detail above and in our manuscript including the Supplementary Information.

Reviewer #3

I believe the authors have satisfactorily addressed the comments of the 3 reviewers.

However, I would recommend some minor changes in nomenclature prior to publication:

(a) Throughout the MS the authors refer to cross signals. For example NH cross signals, CH cross signals, etc..... Cross signals is NOT a term used in the NMR literature. The authors are actually referring to "cross-peaks". And when they are talking for example about "NH cross signals", what they are really talking about are "1H-15N cross-peaks". It's important to get this right, not because one can't figure out what the authors are talking about, but rather because their notation gives the impression that they don't know anything about NMR.

> *Response:* We are very thankful to the reviewer for pointing out the importance of referring to “cross-peak” and not to “cross signal” when evaluating two-dimensional NMR data as done in our manuscript. Accordingly, we have continuously changed this terminology in the entire manuscript including Supplementary Information.

(b) The authors are confused in terms of equating peak height (either the height of a resonance in a 1D spectrum or that of a cross-peak in a 2D correlation spectrum) with intensity. This is incorrect. Intensity refers to the area under a one-dimensional signal and the volume of a cross-peak in a 2D (or higher dimensional) spectrum. Recall intensity in NMR refers to magnetization, and magnetization relates to the integral under a resonance or cross-peak, NOT to peak height which is dictated by the transverse relaxation time(s). So keep it simple and refer explicitly to cross-peak (2D) height and cross-peak volumes/intensity. (And similar for the 1D case: signal height versus area under the signal which really can't be determined from 1D in the presence of any sort of significant overlap).

Response: We fully agree with the reviewer that the height of a peak is not equal to the area (integral) under a one-dimensional NMR signal and the volume of a cross-peak in a multidimensional NMR spectrum, respectively. Thus, we have modified our manuscript on pages 6, 8, 17, and 18 to exclude potential confusion in this regard. Additionally, we have modified insets of all 14 panels comprising Supplementary Figure 8 and changed labeling on y-axis of Supplementary Figure 4A-C, G-I; Supplementary Figure 5A, B; Supplementary Figure S8C, G, K, E, I, M; Supplementary Figure 13A-C.

We would like to thank the reviewer for this valuable advice.

Please note that our manuscript contains detailed information regarding NMR data analysis which is based on either using the height or the volume of an individual cross-peak being present in a two-dimensional NMR spectrum (Supplementary Figure 8).
